# Dietary factors and risk of islet autoimmunity and type 1 diabetes: a systematic review and meta-analysis

**DOI:** 10.1016/j.ebiom.2021.103633

**Published:** 2021-10-14

**Authors:** Anna-Maria Lampousi, Sofia Carlsson, Josefin E. Löfvenborg

**Affiliations:** Institute of Environmental Medicine, Karolinska Institutet, Stockholm, Sweden

**Keywords:** Type 1 diabetes, Islet autoimmunity, Diet, Meta-analysis

## Abstract

**Background:**

Numerous dietary components have been linked to the development of islet autoimmunity (IA) and type 1 diabetes (T1D); however, no associations are firmly established. This systematic review and meta-analysis aimed to synthesize current knowledge on diet and incidence of IA and T1D.

**Methods:**

Literature search was performed in Medline, Embase, and Cochrane Library, from inception until October 2020. Eligible studies had IA or T1D as outcome; any dietary exposure; case-control, cohort, or randomized controlled trial design; and hazard, risk, or odds ratios as measures of association. Summary relative risks (RR) and 95% confidence intervals (CI) were estimated with random-effects models. Certainty of evidence was assessed with GRADE. PROSPERO registration number: CRD42020212505.

**Findings:**

Among 5935 identified records, 96 were eligible, and pooled estimates could be produced for 26 dietary factors. Evidence with moderate/high certainty indicated lower risk of T1D in relation to longer (≥6-12 vs <6-12 months, RR: 0⋅39, CI: 0⋅26-0⋅58, I^2^=43%) and exclusive (≥2-3 vs <2-3 months, RR: 0⋅68, CI: 0⋅58-0⋅80, I^2^=0%) breastfeeding, later introduction to gluten (3-6 vs <3-5 months, RR: 0⋅36, CI: 0⋅17-0⋅75, I^2^=0%), cow's milk (≥2-3 vs <2-3 months, RR: 0⋅69, CI: 0⋅59-0⋅81, I^2^=0%), and fruit (4-6 vs <4-5 months, RR: 0⋅47, CI: 0⋅25-0⋅86, I^2^=0%). Higher childhood intake of cow's milk was associated with increased risk of both IA (per 2-3 portions/day, RR: 1⋅25, CI: 1⋅06-1⋅47, I^2^=0%) and T1D (≥2-3 vs <2-3 glasses/day, RR: 1⋅81, CI: 1⋅12-2⋅91, I^2^=31%). For the remaining dietary factors investigated, there was no association, or the evidence was of low certainty.

**Interpretation:**

This study suggests that breastfeeding and late introduction of gluten, fruit, and cow's milk may reduce the risk of T1D, whereas high childhood cow's milk intake may increase it.

**Funding:**

Swedish Research Council, Swedish Research Council for Health, Working Life and Welfare (FORTE), Novo Nordisk Foundation, and Swedish Diabetes Foundation.

**Research in context**

**Evidence before this study**

Over the past decades, several dietary exposures at different developmental stages have been assessed in relation to type 1 diabetes (T1D), and many of those have been proposed as potential risk factors. Previous meta-analyses assess a limited number of dietary factors and ours is the first attempt to synthesize the totality of evidence regarding the influence of diet on T1D risk. We searched Medline (Ovid), Embase, and Cochrane Library (Wiley) for cohort and case-control studies, as well as randomized controlled trials (RCTs), published until October 15^th^, 2020, in English, that evaluated the relative risk of islet autoimmunity (IA) or T1D in relation to any dietary factor during the life course, and did not have critical risk of bias. Certainty of evidence was evaluated for each association.

**Added value of this study**

It was possible to synthesize data for 26 dietary exposures in utero, infancy, childhood, and/or adulthood in relation to IA and T1D from 96 observational studies. Evidence rated with moderate or high certainty suggested a reduced risk of T1D in relation to longer breastfeeding and later introduction to gluten, cow's milk, and fruit, and an increased risk of IA conferred by high childhood intake of cow's milk. Associations were also seen between T1D and childhood intakes of carbohydrates, sugar, sugar sweetened beverages, protein, meat, nitrite, vitamins A and C, as well as vitamin D supplementation in infancy. However, the certainty of this evidence was graded as low. The small number of identified RCTs has failed to find beneficial effects of dietary interventions.

**Implications of all the available evidence**

This study supports that diet may play a role in the development of T1D. However, an important conclusion is that although many dietary factors are associated with T1D, the certainty of this evidence is low. There is a clear need for future high quality observational studies, preferable with objectively assessed nutritional information and detailed adjustments for confounders, to further elucidate the influence of diet in the etiology of T1D. We also need clinical trials to evaluate if diet modification may indeed prevent T1D.

Alt-text: Unlabelled box

## 1 Introduction

Type 1 diabetes (T1D) is an autoimmune disease characterized by destruction of the insulin producing beta-cells that results in chronic dependence on exogenous insulin [1]. The term islet autoimmunity (IA) describes the presence of islet autoantibodies in serum, which precedes diagnosis and is indicative of the start of the autoimmune process that may lead to T1D [2]. The disease often occurs in childhood and its incidence has been increasing worldwide over the past decades [3], [4], [5]. Genetic factors are important in the development of T1D, especially genes in the human leukocyte antigen (HLA) region [6,7]. However, environmental factors may also play a role either as triggers or promotors of the autoimmune reaction, and the rising incidence of T1D provides strong support for this notion.

Despite decades of research, the role of environmental factors in the etiology of T1D remains unclear [8]. Dietary factors are in the spotlight since many years and multiple dietary exposures have been linked to the development of IA or T1D [9]. The observed associations are hypothetically explained by effects of diet on the maturation of gut microbiota, immune response, and prevention of oxidative stress [10,11]. Still, no firm associations are established and a systematic synthesis of the evidence remains to be conducted. Meta-analyses in this field are scarce and focus on individual dietary exposures such as breastfeeding [12], [13], [14], [15], cow's milk [14], [15], [16], vitamin D [17], [18], [19], [20], and fatty acids [21]. Distinguishing between fetal, infancy, and childhood exposures seems warranted, since any effects may differ between developmental stages. Our aim was therefore to clarify the relationship between diet and T1D by synthesizing current knowledge on the association between diet and incidence of IA and T1D, in a systematic review and meta-analysis. This is important since diet constitutes a modifiable exposure that could be a promising component in strategies for the prevention or delay of T1D.

## 2 Methods

### 2.1 Search strategy and selection criteria

A systematic review and meta-analysis was conducted after registration in the International Prospective Register for Systematic Reviews (PROSPERO) https://www.crd.york.ac.uk/prospero/display_record.php?ID=CRD42020212505. This study adheres to the PRISMA guidelines. Eligible studies were in English, had IA or T1D as outcome; nutrients, foods, or beverages, or nutritional biomarker levels as exposure; cohort, case-cohort, case-control, nested case-control, or randomized controlled trial (RCT) design; and hazard ratios, risk ratios, or odds ratios with 95% confidence intervals (CI), as measures of association. In the present paper, the term relative risk (RR) was used for all measures of association. Congress papers, editorials, interviews, letters, and animal studies were excluded.

Medline (Ovid), Embase, and Cochrane Library (Wiley) were searched from inception until October 15^th^, 2020, by librarians at Karolinska Institutet University Library (for complete search strategy see Supplementary Tables 1-3). Relevant studies were retrieved from the reference lists of eligible articles. Title and abstract of all identified studies were screened and articles that seemed likely to fulfill the eligibility criteria regarding the exposure and outcome definitions, as well as the study design, were fully examined. Study screening, data extraction, and risk of bias and certainty of evidence assessment were performed independently by AML and JEL, and disagreements were resolved by consultation with SC.

### 2.2 Data extraction

From eligible articles we extracted name of first author, publication year, country, cohort name (when applicable), study design, sample size, number of cases, sex, age at diagnosis, type and quantity of exposure, reference group, outcome, method of exposure and outcome assessment, presence of IA at baseline, risk genotypes, family history of T1D, follow-up time, RR with 95% CI, and included covariates. When multiple estimates were available, the most adjusted was extracted and when only separate estimates of different population strata (e.g., race, genetic background) were provided, they were pooled using fixed-effects models before inclusion in meta-analysis. Additionally, when several articles used the same data, only one of these was included (Supplementary Table 4). We contacted the corresponding author when vital information was missing and if no response was obtained, the study was excluded.

### 2.3 Risk of bias assessment

Risk of bias was assessed with the Risk of Bias in Non-randomized Studies of Interventions (ROBINS-I) [22] and the revised tool for Risk of Bias in Randomized Trials (RoB 2) [23]. ROBINS-I assesses confounding, selection of participants into the study, classification of interventions, deviations from intended interventions, missing data, outcome measurement, and selection of reported result. Each domain is graded with low, moderate, serious, or critical risk of bias. We considered a study as having critical risk of confounding if the estimates were not adjusted for age and at least one more potential confounder. Studies adjusting for age and at least one more potential confounder, but without controlling for maternal risk factors when relevant, genetic risk, or other dietary co-exposures, were rated as having serious risk of confounding. Otherwise, the study was rated as having moderate risk of confounding. RoB 2 assesses the same domains, but confounding, selection of participants, and classification of interventions are replaced by the randomization process. For both tools, the overall grade comes from the domain with the highest risk of bias. Studies with critical risk of bias were excluded from data synthesis.

### 2.4 Statistics

Statistical analyses were performed with Stata Statistical Software Release 16 (StataCorp). Random-effects models were used for estimating summary RR and 95% CI of IA and T1D in relation to the highest versus lowest or continuous exposure to each dietary factor, depending on the availability of estimates. The cut-offs of categorical exposures differed across studies and are therefore presented as ranges in the meta-analyses. Thus, the range represents the cut-offs used in the individual studies, e.g., the cut-off ≥2-3 vs <2-3 months means that some of the included studies used the cut-off of two months, and others used three months. Exposure definitions in individual studies are presented in Supplementary Table 6. If the exposure categorizations were too diverse to pool, we present the RR from the meta-analysis based on the largest number of studies, in Figs. 2 and 3. For some dietary factors, the available estimates were corresponding to both continuous and categorical exposure. In such cases, we derived dose-response RR for categorical exposures with at least three categories, before combining them with continuous exposures, using the methods described by Greenland and Longnecker [24,25]. When it was not possible to derive dose-response RR (e.g., for binary exposures) we meta-analyzed estimates corresponding to both continuous and categorical exposures, which allowed us to at least assess the direction of the potential associations. Weights were assigned to each study based on the inverse variance of their estimate and between study variance was estimated with the restricted maximum likelihood method. In meta-analyses of T1D, all studies of T1D were included, independently of whether they were assessing progression from IA or not. However, when enough studies were available, the risk of progressing from IA to T1D was assessed in separate analysis. Prenatal and postnatal exposures were analyzed separately.

Heterogeneity across studies was assessed with the Cochran's Q test and the percentage of observed variance due to heterogeneity with the I^2^ statistic (I^2^>50% indicates substantial heterogeneity). To investigate the source of heterogeneity, subgroup analyses based on study design, risk of bias, and genetic susceptibility, were performed when I^2^ >50% and at least five studies were available. We also performed post hoc sensitivity meta-analyses restricted to prospective data for the outcome T1D; for the outcome IA all studies had a prospective design. Small study effects were assessed for meta-analyses with at least 10 studies with the Egger's test and contour-enhanced funnel plots with critical regions at 1%, 5% and 10% significance levels were used to discriminate whether small study effects were attributable to publication bias.

### 2.5 Certainty of meta-evidence

The certainty of evidence for each meta-analysis was assessed through the Grading of Recommendations, Assessment, Development and Evaluations (GRADE) framework, which classifies the certainty of evidence as ‘very low’, ‘low’, ‘moderate’, and ‘high’ [26]. The certainty of evidence begins with 'high' rating for RCTs, as well as for observational studies assessed for bias with the ROBINS-I tool [27]. Reviewers may downgrade the certainty based on the presence of risk of bias, imprecision, inconsistency, indirectness, and publication bias [28]. Certainty increases if there is a large effect (RR <0⋅5 or RR >2), a dose-response gradient, or if residual confounding would attenuate an association [28].

### 2.6 Role of the funding source

The funders had no role in study design, data collection, analysis, interpretation, or writing of the report.

## 3 Results

### 3.1 Characteristics

Out of 5,935 articles initially screened, 96 studies with a cohort (n=42) or case-control (n=54) design could be meta-analyzed (Fig. 1). Of these, 46 were rated with moderate and 50 with serious risk of bias. Most studies were conducted in Europe (n=71), followed by North America (n=25), Asia (n=3), Australia (n=3), Africa (n=1), and South America (n=1). Details of these studies are presented in Supplementary Table 6. Quantitative synthesis could be performed for 26 dietary factors (Supplementary Table 5); summary RR and 95% CI of T1D and IA in relation to these factors are shown in Fig. 2, Fig. 3. Forest plots for all the individual meta-analyses are given in Supplementary Figs. 1-67. The risk of progression from IA to T1D was only possible to assess in relation to plasma levels of 25-hydroxyvitamin D (25(OH)D) in childhood, due to lack of adequate studies on the remaining exposures (Supplementary Fig. 65).

**Fig. 1:**
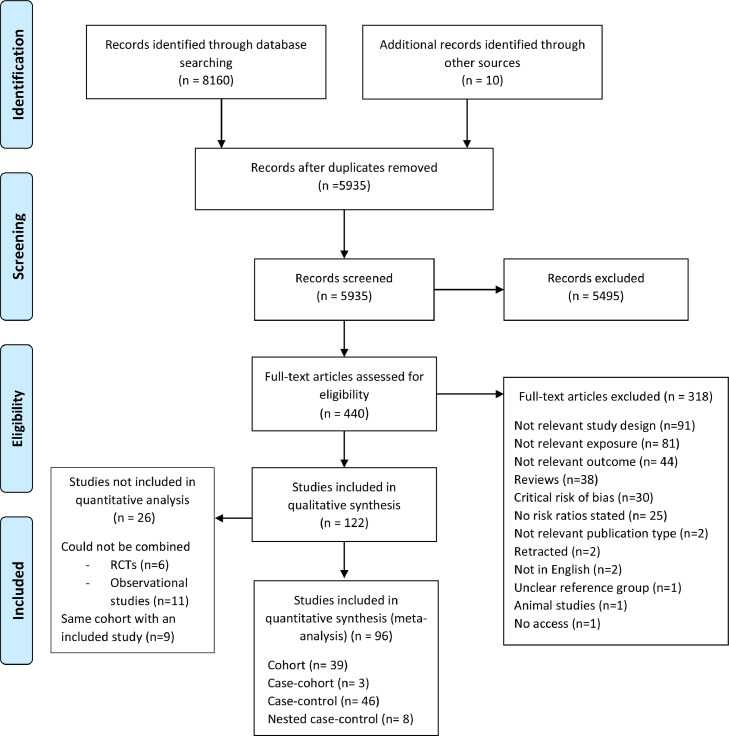
Flow diagram of study selection

**Fig. 2:**
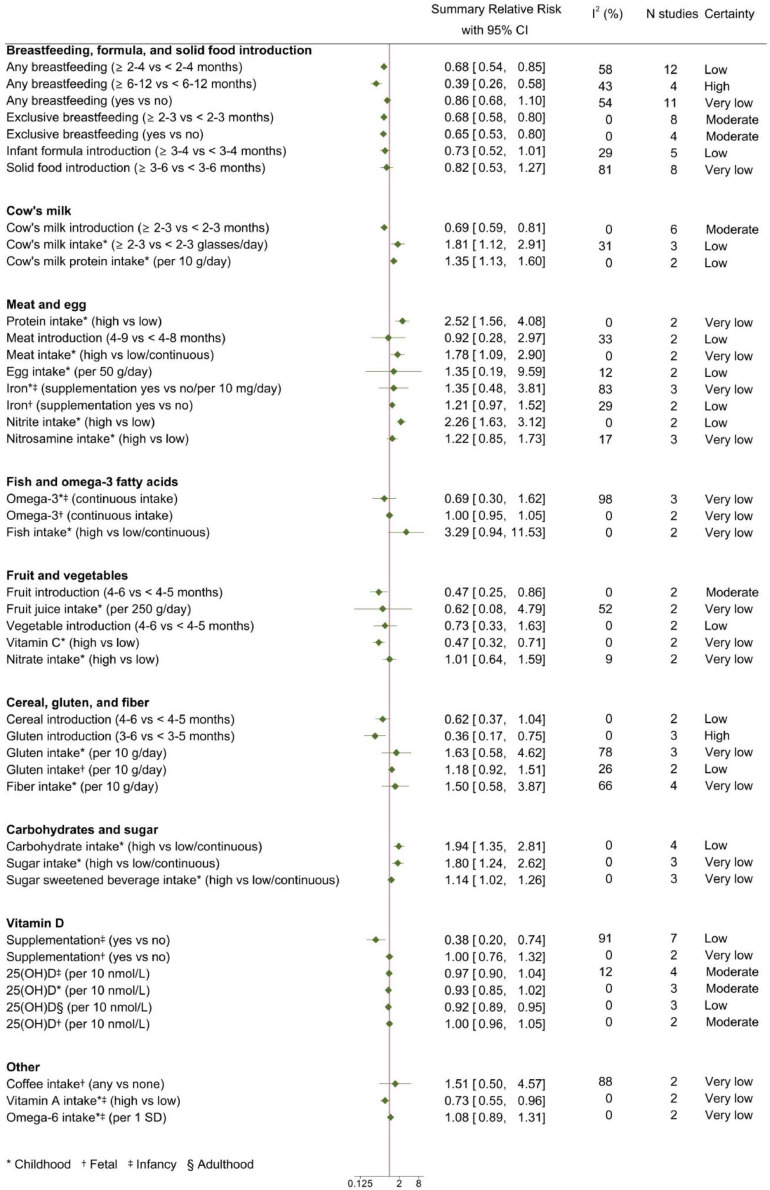
Summary relative risks and 95% confidence intervals of type 1 diabetes in relation to diet

**Fig. 3:**
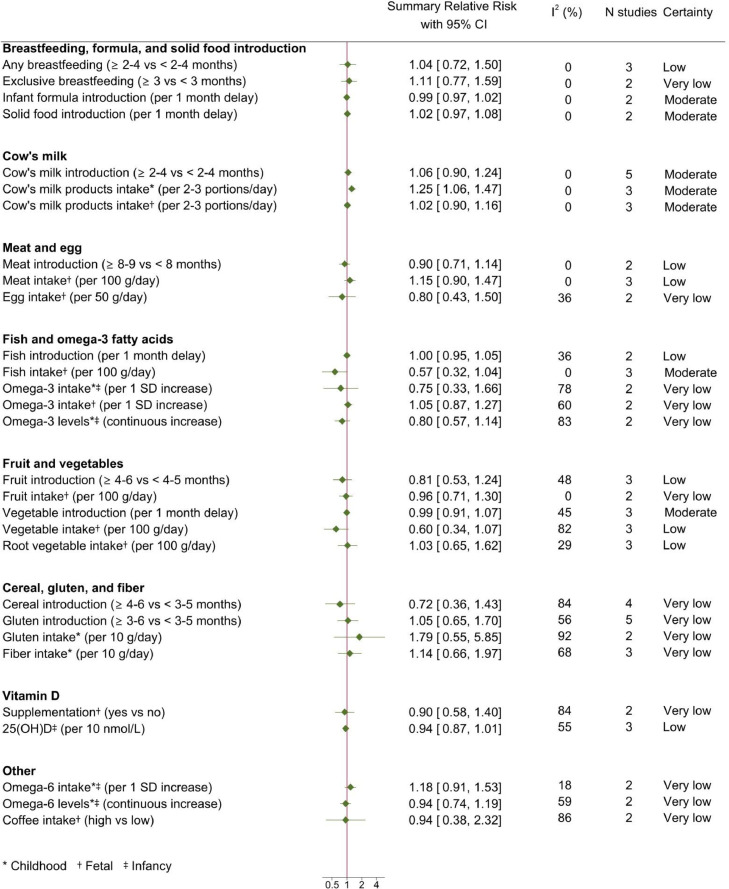
Summary relative risks and 95% confidence intervals of islet autoimmunity in relation to diet

## 4 Breastfeeding, infant formula, and solid food introduction

Longer durations of any or exclusive breastfeeding were inversely associated with T1D (Fig. 2). The largest risk reduction was observed for ≥6-12 versus <6-12 months of any breastfeeding (RR: 0⋅39, 95% CI 0⋅26-0⋅58, I^2^=43%). A tendency towards an inverse association was noted for later introduction to infant formula, whereas age at introduction to solid food was unrelated to T1D, although with substantial heterogeneity across studies (Fig. 2). Neither breastfeeding nor introduction to infant formula or solid food were associated with IA (Fig. 3).

### 4.1 Cow's milk

Later introduction to cow's milk (≥2-3 versus <2-3 months) was inversely associated with T1D (Fig. 2), whereas no association was seen with IA (Fig. 3). Higher childhood intake of cow's milk products conferred an increased risk of both T1D and IA, with minor heterogeneity across studies (Figs. 2 and 3). Similarly, higher intake of cow's milk protein was associated with increased risk of T1D (Fig. 2).

### 4.2 Meat and egg

For T1D, positive associations were observed in relation to childhood intake of meat, protein, and nitrite, with no heterogeneity between studies (Fig. 2). In contrast, the incidence of T1D appeared unrelated to age at introduction to meat, childhood intake of egg, iron, and nitrosamine, and fetal exposure to iron supplementation. IA was only investigated in relation to age at introduction to meat and fetal exposure to meat and egg, with no indication of an association (Fig. 3).

### 4.3 Fish and omega-3 fatty acids

The results were compatible with a reduced risk of T1D (RR: 0⋅69, 95% CI 0⋅30-1⋅62, I^2^=98%) and IA (RR: 0⋅75, 95% CI 0⋅33-1⋅66, I^2^=78%) in relation to higher childhood intake of omega-3 fatty acids, but the associations were not significant and there was substantial heterogeneity (Figs. 2 and 3). Fetal exposure to maternal omega-3 intake was not associated with neither T1D nor IA. However, there was indication of a reduced risk of IA in the offspring in relation to maternal fish intake during pregnancy (RR: 0⋅57, 95% CI 0⋅32-1⋅04, I^2^=0%), but no data on a potential association with T1D. There was no indication of a reduced risk of T1D in relation to childhood fish intake.

### 4.4 Fruit and vegetables

An inverse association with T1D was observed for age at introduction to fruit (RR: 0⋅47, 95% CI 0⋅25-0⋅86), with a similar tendency for age at introduction to vegetables (RR: 0⋅73, 95% CI 0⋅33-1⋅63) and childhood intake of fruit juice (RR: 0⋅62, 95% CI 0⋅08-4⋅79) (Fig. 2). Higher intakes of vitamin C and vitamin A in childhood, from dietary sources or supplements, were also associated with a reduced risk of T1D (Fig. 2). There were no significant associations between IA and fetal exposure or age at introduction to fruit and vegetables (Fig. 3). Data on the risk of IA in relation to childhood exposure to fruit, vegetables, or vitamin C, were lacking.

### 4.5 Cereal, gluten, and fiber

Later introduction to gluten (3-6 versus <3-5 months) was associated with reduced T1D risk (RR: 0⋅36, 95% CI 0⋅17-0⋅75, I^2^=0%) with a similar tendency for cereal introduction (Fig. 2). No such associations were observed for IA, but heterogeneity was high (I^2^=56% and I^2^=84%, respectively) and, for gluten introduction, possibly explained by genetic susceptibility (Supplementary Fig. 77). Gluten intake in childhood showed a tendency towards a positive association with both T1D and IA but confidence intervals were wide, and heterogeneity substantial (Figs. 2 and 3).

### 4.6 Carbohydrates and sugar

There were positive associations between T1D and childhood intake of carbohydrates, sugar, and sugar-sweetened beverages, which was most pronounced for carbohydrates (RR: 1⋅94, 95% CI 1⋅35-2⋅81, I^2^=0%) and without any indication of heterogeneity across studies (Fig. 2). These factors could not be meta-analyzed for IA (Supplementary Table 9).

### 4.7 Vitamin D

Vitamin D supplementation during infancy was inversely related to T1D (Fig. 2), but the between-study heterogeneity was high, and its sources could not be identified (Supplementary Fig. 78). A reduced risk of T1D was also observed in relation to serum levels of 25(OH)D in adulthood (Fig. 2). Neither prenatal exposure nor serum or plasma levels of 25(OH)D in infancy or childhood were associated with T1D (Fig. 2). Plasma levels of 25(OH)D in childhood could be meta-analyzed in relation to progression from IA to T1D (Supplementary Fig. 65) and there was no indication of an inverse association (RR: 0⋅97, 95% CI 0⋅85-1⋅09, I^2^=0%).

The risk of T1D and IA was also investigated in relation to omega-6 fatty acid intake during childhood and maternal coffee intake, but no associations were observed (Figs. 2 and 3).

### 4.8 Publication bias and sensitivity analyses

The presence of publication bias could be assessed for meta-analyses investigating the risk of T1D in relation to any breastfeeding and cow's milk introduction (Supplementary Figs. 68-70). No publication bias was observed through visual inspection of the funnel plots, however, the Egger's test indicated presence of small study effects for any breastfeeding (p=0.0059). Meta-analyses restricted to prospective studies of T1D (25 out of 80 studies) could be performed for less than half of the dietary factors, including breastfeeding (any and exclusive); age at infant formula, solid food, meat, fruit, vegetable, cereal, and gluten introduction; gluten and fiber intake during childhood; maternal, infant, childhood, and adult vitamin D levels; and omega-3 and omega-6 intake during infancy or childhood (Supplementary Fig. 79). These analyses revealed significantly reduced risks of T1D in relation to later fruit and gluten introduction and higher 25(OH)D levels during adulthood (Supplementary Fig. 79). Only one prospective study assessed the risk of T1D in relation to longer breastfeeding (≥12 versus <12 months) and showed an inverse association (RR: 0⋅37, 95% CI 0⋅15-0⋅93).

### 4.9 Certainty of meta-evidence

**Fig. 4:**
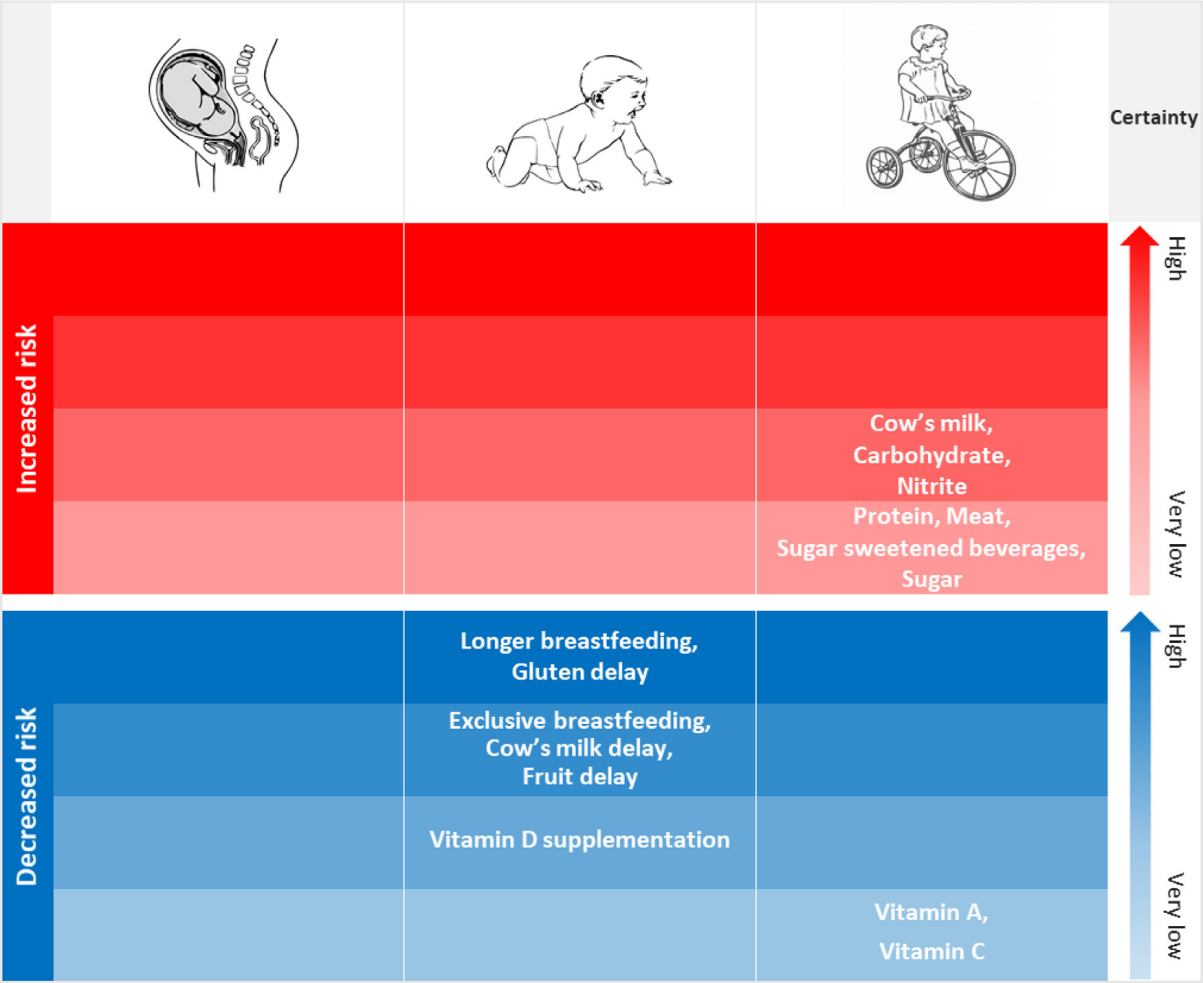
Certainty of evidence of the associations between dietary factors at different developmental stages and incidence of type 1 diabetes, based on the GRADE tool.

Associations between T1D and breastfeeding (≥6-12 versus <6-12 months) and age at introduction to gluten, were rated with high certainty, and associations with exclusive breastfeeding, age at introduction to cow's milk, and fruit, with moderate certainty (Fig. 4). For IA, the association with cow's milk intake was of moderate certainty. Certainty was low or very low for the remaining associations. Details of the certainty of evidence assessment of each meta-analysis are presented in Supplementary Table 10.

### 4.10 Studies that could not be meta-analyzed

Studies that were not meta-analyzed include 20 observational studies and six RCTs that either had overlapping study populations or were evaluating different dietary factors (Supplementary Tables 7-9). The observational studies assessed introduction to probiotics, maternal micronutrient intakes, and nutritional biomarker levels during infancy and childhood. The RCTs assessed the effects of weaning to an extensively hydrolyzed versus cow's milk-based formula, introduction to gluten at 12 months versus six months, and nicotinamide supplementation in childhood. No beneficial effects were seen for these factors (Supplementary Table 7).

## 5 Discussion

This meta-analysis included 26 dietary exposures assessed in 96 studies. The results indicate positive associations between T1D or IA and childhood intake of cow's milk, carbohydrates, sugar, sugar-sweetened beverages, protein, meat, and nitrite. In contrast, inverse associations were observed with breastfeeding, later introduction to cow's milk, fruit, and gluten, vitamin D supplementation in infancy, 25(OH)D levels in adulthood, and childhood intake of vitamin A and C. None of the fetal dietary exposures were related to IA or T1D. The certainty of evidence was high for a reduced risk relating to longer breastfeeding and later introduction to gluten; and moderate for an increased risk associated with higher intake of cow's milk, and a reduced risk with exclusive breastfeeding, and later introduction to cow's milk and fruit. For the remaining dietary factors, certainty was low (Fig. 4). This is the first meta-analysis that aimed at covering all dietary factors ever investigated in relation to the risk of IA or T1D.

Our results in favor of beneficial effects of breastfeeding are consistent with previous meta-analyses,[12,13] and a recently published systematic review [29]. As to potential mechanisms, breastfeeding may protect against autoimmune diseases by transferring maternal antigens to the infant and enhancing its microbiota [30]. Breastfeeding could also reduce the risk of T1D indirectly by delaying introduction of foods that may hypothetically trigger an autoimmune reaction. In line with this reasoning, age at introduction to several dietary factors including gluten, fruit, and cow's milk was inversely associated with T1D. Our findings suggest that it may be particularly beneficial to delay introduction to gluten. Evidence in mice indicate that gliadin, a gluten protein, can cross the gut barrier and cause beta-cell disruptions [31]. This might be particularly relevant in early life when gut permeability is increased. Nevertheless, it is not clear whether delaying the introduction to gluten for longer than six months may confer additional benefits. Notably, a small RCT found no difference in the risk of IA when comparing gluten introduction at 12 months and six months [32].

Our study also indicated adverse effects of cow´s milk both in terms of quantities consumed in childhood and early introduction. It has been speculated that the excess risk of T1D associated with earlier introduction to cow´s milk may reflect lack of breastfeeding [33]. In support hereof, a large RCT that compared weaning to extensively hydrolyzed formula versus cow´s milk-based formula failed to find beneficial effects [34]. On the other hand, proteins found in cow's milk contain amino acids with similar structure to human tissues, including the pancreatic islets, and they may enter the circulatory system undigested and trigger autoimmune response [35]. This might explain the observed associations between cow's milk consumption in childhood and risk of T1D and IA. Regarding other types of animal milk, milk supplements, or plant-based milk, no such studies were identified.

Carbohydrates, sugar, and sugar-sweetened beverages were associated with increased risk of T1D, although certainty of this evidence was low primarily owing to serious risk of bias in the individual studies. The underlying mechanism could involve effects on body weight, since adiposity is associated with increased risk of T1D [36]. In addition, in vitro models have shown that intermittent elevations of glucose levels may lead to beta-cell apoptosis [37]. The risk of T1D also increased with childhood exposure to nitrite, protein, and their common main source, meat. Toxic effects on the beta-cells have been observed after exposure to the N-nitroso compounds formulated after nitrite consumption [38]. The results should however be interpreted with caution as the certainty of this evidence was low.

Vitamin D is suggested to play a role in the etiology of T1D, primarily due to its involvement in the regulation of the immune system [39]. We found evidence with low certainty, mainly due to high heterogeneity and risk of confounding and recall bias, for a beneficial effect of vitamin D supplementation in infancy, which is in line with the results of a previous meta-analysis [17]. Still, circulating levels of 25(OH)D in early life were not associated with IA and T1D. This might indicate that vitamin D sufficiency, rather than higher levels, is implicated in T1D etiology.

Intake of antioxidants may hypothetically reduce the risk of T1D by inhibiting oxidative stress. Consistent with this hypothesis, we found a reduced risk of T1D in relation to vitamin A and C exposure during childhood, although the certainty of this evidence was very low.

Strengths include a broad and systematic literature search according to an a priori defined protocol, and assessment of different types of bias in the individual studies and of the overall quality of evidence for each dietary factor that was meta-analyzed. By performing study selection, data extraction, and risk of bias assessment in duplicate, we reduced the risk of missing important information and introducing errors. Additionally, we distinguished between exposures in utero, infancy, and childhood. The main limitation is that the number of studies was small for most dietary factors. This reduced power and the possibilities to perform sub-group analyses, assess dose-response relationships, and publication bias. Several of the included studies had serious risk of bias, mainly due to confounding, and potential misclassification of exposure caused by self-reported dietary information. The latter is a particular concern in case-control studies with retrospective information. This resulted in reduced certainty of evidence for most associations. Importantly, biomarker information was used in some studies, which reduces this bias. The GRADE tool does not distinguish between prospective and retrospective evidence. However, we performed sensitivity analyses restricted to prospective studies, which substantially reduced the number of eligible studies but revealed similar associations for the dietary factors that could be analyzed. In parallel, RCTs were few and not possible to synthesize since the interventions differed. A main concern regarding confounding was the lack of adjustment for other dietary factors, considering that no food is consumed in isolation. Associations were generally weaker for IA than for T1D. This could reflect that IA is a less specific outcome, identified by the presence of one or several different autoantibodies with varying positivity cut-offs. The assessment of dietary factors varied within meta-analyses both in terms of definition, timing, and categorization. This implies that some studies could not be included in the meta-analyses, e.g., if they had a unique exposure assessment that did not align with that of other studies. Most importantly, it precludes formulation of specific recommendations, e.g., regarding optimal age at introduction of certain foods, quantities of milk consumption, and duration of breastfeeding. Finally, it should be noted that most studies came from Europe and North America and whether the results apply to other populations with different genetic makeup and dietary habits remains to be explored.

Our findings indicate that longer breastfeeding and delayed introduction to gluten, cow's milk, and fruits may prevent T1D. Moreover, we find support for the general recommendation of vitamin D supplementation in early life. The potential harm in terms of T1D risk of high cow's milk consumption in childhood may be considered in risk-benefit analyses underlying dietary guidelines. There were several dietary factors previously linked to T1D for which we did not find associations including iron, fish, and omega-3 fatty acids and as noted above, many of the observed associations were of low certainty. This emphasizes the need for future studies to elucidate the role of diet in the etiology of T1D and these studies should preferably use a prospective design, nutritional biomarkers, and careful adjustment for potential confounders. Moreover, the potential interaction of genetic susceptibility with diet needs to be explored. Few studies investigated dietary factors in relation to progression from IA to T1D. This is an important topic for future studies as it may provide information to be used for prevention. In addition, distinguishing between stage 1 (IA with normoglycemia) and stage 2 (IA with dysglycemia) of presymptomatic T1D, when studying progression, may provide further insights in the etiology of T1D [40]. Importantly, RCTs conducted so far, focusing mainly on infant feeding, have failed to find protective effects of dietary interventions. Future studies elucidating whether the observed associations are causal and whether diet modification can prevent T1D in practice are needed.

In conclusion, this study supports that infant and childhood diet may influence the risk of T1D. The most convincing evidence was seen for beneficial effects of breastfeeding, later introduction to gluten, cow´s milk, and fruit, and lower consumption of cow´s milk during childhood. Other associations were observed, but the certainty of evidence was low. The results emphasize the need for future, high quality studies on the influence of diet on development of T1D.

## 6 Contributors

The study was conceived by SC together with AML and JEL. AML and JEL independently performed the study selection, data extraction, and risk of bias and certainty of evidence assessment and consulted SC in case of disagreement. AML did the statistical analyses and wrote the first draft with input from JEL and SC, who also edited the manuscript. All authors contributed to the interpretation of the findings, had full access to all the data and final responsibility for the decision to submit for publication.

## Declaration of competing interest

The authors declare no competing interests.

## Appendix Supplementary materials

Image, application 1
