# Supplementary material for: Dietary factors and risk of islet autoimmunity and type 1 diabetes: a systematic review and meta-analysis
Source: eBioMedicine. 2021 Oct 14;72:103633. doi: 10.1016/j.ebiom.2021.103633 (PMC8523874; doi:10.1016/j.ebiom.2021.103633)
Supplement: Supplementary file 1 [file mmc1.docx]

**Supplementary material**

List of Supplementary Tables

[Supplementary Table 1. Search strategy for identification of relevant articles in Medline 3](#_Toc81850611)

[Supplementary Table 2. Search strategy for identification of relevant articles in Embase 5](#_Toc81850612)

[Supplementary Table 3. Search strategy for identification of relevant articles in Cochrane Library 6](#_Toc81850613)

[Supplementary Table 4. Criteria for inclusion among studies using the same data 6](#_Toc81850614)

[Supplementary Table 5. Dietary factors included in meta-analyses 6](#_Toc81850615)

[Supplementary Table 6. Characteristics of the studies that were included in the meta-analysis 7](#_Toc81850616)

[Supplementary Table 7. Characteristics of eligible randomized controlled trials 30](#_Toc81850617)

[Supplementary Table 8. Characteristics of eligible observational studies that were excluded because other studies on the same cohort were selected 31](#_Toc81850618)

[Supplementary Table 9. Characteristics of associations that could not be meta-analyzed 33](#_Toc81850619)

[Supplementary Table 10. Quality of evidence rating using the GRADE system 53](#_Toc81850620)

List of Supplementary Figures

[Supplementary Figure 1. Any breastfeeding and IA (long vs short) 63](#_Toc81850648)

[Supplementary Figure 2. Any breastfeeding and IA (per additional month) 63](#_Toc81850649)

[Supplementary Figure 3. Any breastfeeding and T1D (long vs short) 64](#_Toc81850650)

[Supplementary Figure 4. Any breastfeeding and T1D (per additional month) 65](#_Toc81850651)

[Supplementary Figure 5. Exclusive breastfeeding and IA (long vs short) 65](#_Toc81850652)

[Supplementary Figure 6. Exclusive breastfeeding and IA (per additional month) 65](#_Toc81850653)

[Supplementary Figure 7. Exclusive breastfeeding and T1D (long vs short) 66](#_Toc81850654)

[Supplementary Figure 8. Exclusive breastfeeding and T1D (per additional month) 66](#_Toc81850655)

[Supplementary Figure 9. Any breastfeeding and T1D (yes vs no) 67](#_Toc81850656)

[Supplementary Figure 10. Exclusive breastfeeding and T1D (yes vs no) 67](#_Toc81850657)

[Supplementary Figure 11. Cow's milk introduction and IA. 68](#_Toc81850658)

[Supplementary Figure 12. Cow's milk introduction and T1D 69](#_Toc81850659)

[Supplementary Figure 13. Infant formula introduction and IA 70](#_Toc81850660)

[Supplementary Figure 14. Infant formula introduction and T1D (late vs early) 70](#_Toc81850661)

[Supplementary Figure 15. Infant formula introduction and T1D (per 1 month delay) 71](#_Toc81850662)

[Supplementary Figure 16. Solid food introduction and IA 71](#_Toc81850663)

[Supplementary Figure 17. Solid food introduction and T1D 72](#_Toc81850664)

[Supplementary Figure 18. Cereal introduction and IA 72](#_Toc81850665)

[Supplementary Figure 19. Cereal introduction and T1D 73](#_Toc81850666)

[Supplementary Figure 20. Gluten introduction and IA 74](#_Toc81850667)

[Supplementary Figure 21. Gluten introduction and T1D 75](#_Toc81850668)

[Supplementary Figure 22. Meat introduction and IA 76](#_Toc81850669)

[Supplementary Figure 23. Meat introduction and T1D 76](#_Toc81850670)

[Supplementary Figure 24. Fish introduction and IA 76](#_Toc81850671)

[Supplementary Figure 25. Fruit introduction and IA 77](#_Toc81850672)

[Supplementary Figure 26. Fruit introduction and T1D 77](#_Toc81850673)

[Supplementary Figure 27. Vegetable introduction and IA 78](#_Toc81850674)

[Supplementary Figure 28. Vegetable introduction and T1D 78](#_Toc81850675)

[Supplementary Figure 29. Cow's milk intake and IA 79](#_Toc81850676)

[Supplementary Figure 30. Cow's milk intake and T1D 79](#_Toc81850677)

[Supplementary Figure 31. Maternal meat intake and IA 80](#_Toc81850678)

[Supplementary Figure 32. Meat intake and T1D 80](#_Toc81850679)

[Supplementary Figure 33. Maternal fish intake and IA 80](#_Toc81850680)

[Supplementary Figure 34. Fish intake and T1D 81](#_Toc81850681)

[Supplementary Figure 35. Egg intake and IA 82](#_Toc81850682)

[Supplementary Figure 36. Egg intake and T1D 82](#_Toc81850683)

[Supplementary Figure 37. Fruit juice intake and T1D 83](#_Toc81850684)

[Supplementary Figure 38. Sugar sweetened beverage intake and T1D 83](#_Toc81850685)

[Supplementary Figure 39. Maternal coffee intake and IA 83](#_Toc81850686)

[Supplementary Figure 40. Coffee intake and T1D 84](#_Toc81850687)

[Supplementary Figure 41. Maternal fruit intake and IA 84](#_Toc81850688)

[Supplementary Figure 42. Maternal vegetable intake and IA 85](#_Toc81850689)

[Supplementary Figure 43. Maternal root vegetable intake and IA 85](#_Toc81850690)

[Supplementary Figure 44. Protein intake and T1D 85](#_Toc81850691)

[Supplementary Figure 45. Gluten intake and IA 86](#_Toc81850692)

[Supplementary Figure 46. Gluten intake and T1D 86](#_Toc81850693)

[Supplementary Figure 47. Carbohydrate intake and T1D 87](#_Toc81850694)

[Supplementary Figure 48. Sugar intake and T1D 87](#_Toc81850695)

[Supplementary Figure 49. Fiber intake and IA 87](#_Toc81850696)

[Supplementary Figure 50. Fiber intake and T1D 88](#_Toc81850697)

[Supplementary Figure 51. Omega-3 intake and IA 89](#_Toc81850698)

[Supplementary Figure 52. Omega-3 intake and T1D 89](#_Toc81850699)

[Supplementary Figure 53. Omega-6 intake and IA 90](#_Toc81850700)

[Supplementary Figure 54. Omega-6 intake and T1D 90](#_Toc81850701)

[Supplementary Figure 55. Vitamin D supplementation and IA 91](#_Toc81850702)

[Supplementary Figure 56. Vitamin D supplementation and T1D 91](#_Toc81850703)

[Supplementary Figure 57. Vitamin A intake and T1D 92](#_Toc81850704)

[Supplementary Figure 58. Vitamin C intake and T1D 92](#_Toc81850705)

[Supplementary Figure 59. Iron intake and T1D 93](#_Toc81850706)

[Supplementary Figure 60. Nitrate intake and T1D 93](#_Toc81850707)

[Supplementary Figure 61. Nitrite intake and T1D 94](#_Toc81850708)

[Supplementary Figure 62. Nitrosamine intake and T1D 94](#_Toc81850709)

[Supplementary Figure 63. Vitamin D levels in blood and IA 95](#_Toc81850710)

[Supplementary Figure 64. Vitamin D levels in blood and T1D 96](#_Toc81850711)

[Supplementary Figure 65. Vitamin D levels in blood and progression from IA to T1D 97](#_Toc81850712)

[Supplementary Figure 66. Omega-3 levels in blood and IA 97](#_Toc81850713)

[Supplementary Figure 67. Omega-6 levels in blood and IA 97](#_Toc81850714)

[Supplementary Figure 68. Contour-enhanced funnel plot of included studies on any breastfeeding (long vs short) and risk of T1D. 98](#_Toc81850715)

[Supplementary Figure 69. Contour-enhanced funnel plot of included studies on any breastfeeding (yes vs no) and risk of T1D. 98](#_Toc81850716)

[Supplementary Figure 70. Contour-enhanced funnel plot of included studies on cow’s milk introduction and risk of T1D. 99](#_Toc81850717)

[Supplementary Figure 71. Subgroup analysis for long vs short any breastfeeding and T1D based on risk of bias and study design 100](#_Toc81850718)

[Supplementary Figure 72. Subgroup analysis for any breastfeeding per additional month and T1D based on risk of bias and study design 101](#_Toc81850719)

[Supplementary Figure 73. Subgroup analysis for any breastfeeding yes vs no and T1D based on risk of bias and study design 102](#_Toc81850720)

[Supplementary Figure 74. Subgroup analysis for late vs early introduction to solid food and T1D based on risk of bias and study design 103](#_Toc81850721)

[Supplementary Figure 75. Subgroup analysis for late vs early introduction to solid food and T1D based on genetic risk 104](#_Toc81850722)

[Supplementary Figure 76. Subgroup analysis for late vs early introduction to gluten and IA based on risk of bias 105](#_Toc81850723)

[Supplementary Figure 77. Subgroup analysis for late vs early introduction to gluten and IA based on genetic risk 106](#_Toc81850724)

[Supplementary Figure 78. Subgroup analysis for vitamin D supplementation and T1D based on risk of bias and study design 107](#_Toc81850725)

[Supplementary Figure 79. Sensitivity analysis based on prospective evidence 108](#_Toc81850726)

[List of Supplementary references 109](#_Toc81850727)

**Supplementary Table 1. Search strategy for identification of relevant articles in Medline**

| Interface: Ovid  Date of Search: 15 Oct 2020  Number of hits: 4572  Comment: In Ovid, two or more words are automatically searched as phrases; i.e. no quotation marks are needed | Field labels   - exp/ = exploded MeSH term - / = non exploded MeSH term - .ti,ab,kf. = title, abstract and author keywords - adjx = adjacent within x words, regardless of order - * = truncation of word for alternate endings |
| --- | --- |
| 1. Diabetes mellitus, type 1/  2. ((diabetes or islet or insulin) adj3 (autoantibod* or autoimmun* or Brittle or insulin-dependent or juvenile or type 1)).ti,ab,kf.  3. (Iddm or T1D or T1DM).ti,ab,kf.  4. or/1-3  5. exp Diet/  6. exp Dietary Supplements/  7. exp Food/  8. exp Fatty Acids, Omega-3/  9. exp Fatty Acids, Omega-6/  10. exp Beverages/  11. Iron, Dietary/  12. exp Nitrates/  13. exp Nitrites/  14. Trace Elements/  15. exp Zinc/  15. Breast Feeding/  16. exp Maternal Nutritional Physiological Phenomena/  17. Prenatal Exposure Delayed Effects/  18. exp Environmental Exposure/  19. ((alcohol* or beverage* or caffeine* or cereal* or coffee or cheese* or diet* or dairy product* or egg* or fatty acid* or fiber* or fibre* or fish* or food* or fruit* or gluten* or grain* or iron* or legume* or macronutrient* or meat* or micronutrient* or milk or multivitamin* or nitrate* or nitrite* or nitrosamine* or nut* or nutrient* or omega-3 or n-3 or omega-6 or n-6 or probiotic* or seafood* or starch* or sugar* or sucrose* or supplement* or sweeten* or trace element* or vegetable* or vitamin* or zinc or yoghurt) adj7 (diabetes or islet or insulin) adj3 (autoantibod* or autoimmun* or Brittle or insulin-dependent or juvenile or type 1)).ti,ab,kf.  20. ((alcohol* or beverage* or caffeine* or cereal* or coffee or cheese* or diet* or dairy product* or egg* or fatty acid* or fiber* or fibre* or fish* or food* or fruit* or gluten* or grain* or iron* or legume* or macronutrient* or meat* or micronutrient* or milk or multivitamin* or nitrate* or nitrite* or nitrosamine* or nut* or nutrient* or omega-3 or n-3 or omega-6 or n-6 or probiotic* or seafood* or starch* or sugar* or sucrose* or supplement* or sweeten* or trace element* or vegetable* or vitamin* or zinc or yoghurt) adj7 (Iddm or T1D or T1DM)).ti,ab,kf.  21. (environment* adj7 (diabetes or islet or insulin) adj3 (autoantibod* or autoimmun* or Brittle or insulin-dependent or juvenile or type 1)).ti,ab,kf.  22. (environment* adj7 (iddm or T1D or T1DM or type 1)).ti,ab,kf.  23. ((breast feed* or breastfeed*) adj7 (diabetes or islet or insulin) adj3 (autoantibod* or autoimmun* or Brittle or insulin-dependent or juvenile or type 1)).ti,ab,kf.  24. ((breast feed* or breastfeed*) adj7 (iddm or T1D or T1DM or type 1)).ti,ab,kf.  25. ((fetal or infant or maternal or prenatal or perinatal or pregnancy) adj3 (diet or exposure* or feed* or food* or intake* or lactation or nutrition*)).ti,ab,kf.  26. ((fetal or prenatal or perinatal) adj7 (iddm or t1d or t1dm or type 1)).ti,ab,kf.  27. or/5-26  28. Risk factors/  29. Incidence/  30. (aetiolog* or develop* or etiolog* or inciden* or pathogenes* or prevent* or protect* or risk*).ti,ab,kf.  31. or/28-30  32. 4 and 27 and 31  33. 32 not (animals not humans).sh.  34. limit 33 to english language  35. limit 34 to (clinical conference or congress or consensus development conference or consensus development conference, nih or editorial or letter)  36. 34 not 35 | |

**Supplementary Table 2. Search strategy for identification of relevant articles in Embase**

| Interface: embase.com  Date of Search: 15 Oct 2020  Number of hits: 2551  Comment: Emtree is the controlled vocabulary in Embase | Field labels   - /exp = exploded Emtree term - /de = non exploded Emtree term - ti,ab = title and abstract - NEAR/x = adjacent within x words, regardless of order - * = truncation of word for alternate endings |
| --- | --- |
| ('insulin dependent diabetes mellitus'/exp/mj OR (((diabetes OR islet OR insulin) NEAR/3 (autoantibod* OR autoimmun* OR brittle OR 'insulin-dependent' OR juvenile OR 'type 1')):ti,ab,kw) OR iddm:ti,ab,kw OR t1d:ti,ab,kw OR t1dm:ti,ab,kw)  AND  ('nutrition'/exp/mj OR 'omega 3 fatty acid'/mj OR 'omega 6 fatty acid'/mj OR 'iron intake'/mj OR 'nitrate'/mj OR 'nitrite'/mj OR 'trace element'/mj OR 'zinc'/mj OR 'breast feeding'/exp/mj OR 'maternal nutrition'/mj OR 'prenatal exposure'/mj OR 'environmental exposure'/exp/mj OR (((alcohol* OR beverage* OR caffeine* OR cereal* OR coffee OR cheese* OR diet* OR 'dairy product*' OR egg* OR 'fatty acid*' OR fiber* OR fibre* OR fish* OR food* OR fruit* OR gluten* OR grain* OR iron* OR legume* OR macronutrient* OR meat* OR micronutrient* OR milk OR multivitamin* OR nitrate* OR nitrite* OR nitrosamine* OR nut* OR nutrient* OR 'omega-3' OR 'n-3' OR 'omega-6' OR 'n-6' OR probiotic* OR seafood* OR starch* OR sugar* OR sucrose* OR supplement* OR sweeten* OR 'trace element*' OR vegetable* OR vitamin* OR zinc OR yoghurt) NEAR/7 (diabetes OR islet OR insulin) NEAR/3 (autoantibod* OR autoimmun* OR brittle OR 'insulin-dependent' OR juvenile OR 'type 1')):ti,ab,kw) OR (((alcohol* OR beverage* OR caffeine* OR cereal* OR coffee OR cheese* OR diet* OR 'dairy product*' OR egg* OR 'fatty acid*' OR fiber* OR fibre* OR fish* OR food* OR fruit* OR gluten* OR grain* OR iron* OR legume* OR macronutrient* OR meat* OR micronutrient* OR milk OR multivitamin* OR nitrate* OR nitrite* OR nitrosamine* OR nut* OR nutrient* OR 'omega-3' OR 'n-3' OR 'omega-6' OR 'n-6' OR probiotic* OR seafood* OR starch* OR sugar* OR sucrose* OR supplement* OR sweeten* OR 'trace element*' OR vegetable* OR vitamin* OR zinc OR yoghurt) NEAR/7 (iddm OR t1d OR t1dm)):ti,ab,kw) OR ((environment* NEAR/7 (diabetes OR islet OR insulin) NEAR/3 (autoantibod* OR autoimmun* OR brittle OR 'insulin-dependent' OR juvenile OR 'type 1')):ti,ab,kw) OR ((environment* NEAR/7 (iddm OR t1d OR t1dm OR 'type 1')):ti,ab,kw) OR ((('breast feed*' OR breastfeed*) NEAR/7 (diabetes OR islet OR insulin) NEAR/3 (autoantibod* OR autoimmun* OR brittle OR 'insulin-dependent' OR juvenile OR 'type 1')):ti,ab,kw) OR ((('breast feed*' OR breastfeed*) NEAR/7 (iddm OR t1d OR t1dm OR 'type 1')):ti,ab,kw) OR (((fetal OR infant OR maternal OR prenatal OR perinatal OR pregnancy) NEAR/3 (diet OR exposure* OR feed* OR food* OR intake* OR lactation OR nutrition*)):ti,ab,kw) OR (((fetal OR prenatal OR perinatal) NEAR/7 (iddm OR t1d OR t1dm OR 'type 1')):ti,ab,kw))  AND  ('risk factor'/mj OR 'incidence'/mj OR aetiolog*:ti,ab,kw OR develop*:ti,ab,kw OR etiolog*:ti,ab,kw OR inciden*:ti,ab,kw OR pathogenes*:ti,ab,kw OR prevent*:ti,ab,kw OR protect*:ti,ab,kw OR risk*:ti,ab,kw)  NOT  ([animals]/lim NOT [humans]/lim) AND [english]/lim AND ([article]/lim OR [article in press]/lim OR [erratum]/lim OR [review]/lim OR [short survey]/lim) | |

**Supplementary Table 3. Search strategy for identification of relevant articles in Cochrane Library**

| Interface: Wiley  Date of Search: 15 Oct 2020  Number of hits: 1037 | Field labels   - ti,ab,kw = title, abstract and author keywords - NEAR/x = adjacent within x words, regardless of order - * = truncation of word for alternate endings |
| --- | --- |
| #1 ((diabetes OR islet OR insulin) NEAR/3 (autoantibod* OR autoimmun* OR brittle OR "insulin-dependent" OR juvenile OR "type 1")):ti,ab,kw #2 (iddm OR t1d OR t1dm):ti,ab,kw #3 #1 or #2  #4 ((alcohol* OR beverage* OR caffeine* OR cereal* OR coffee OR cheese* OR diet* OR "dairy product*" OR egg* OR "fatty acid*" OR fiber* OR fibre* OR fish* OR food* OR fruit* OR gluten* OR grain* OR iron* OR legume* OR macronutrient* OR meat* OR micronutrient* OR milk OR multivitamin* OR nitrate* OR nitrite* OR nitrosamine* OR nut* OR nutrient* OR omega-3 OR n-3 OR omega-6 OR n-6 OR probiotic* OR seafood* OR starch* OR sugar* OR sucrose* OR supplement* OR sweeten* OR "trace element*" OR vegetable* OR vitamin* OR zinc OR yoghurt) NEAR/7 (diabetes OR islet OR insulin) NEAR/3 (autoantibod* OR autoimmun* OR brittle OR "insulin-dependent" OR juvenile OR "type 1")):ti,ab,kw #5 ((alcohol* OR beverage* OR caffeine* OR cereal* OR coffee OR cheese* OR diet* OR "dairy product*" OR egg* OR "fatty acid*" OR fiber* OR fibre* OR fish* OR food* OR fruit* OR gluten* OR grain* OR iron* OR legume* OR macronutrient* OR meat* OR micronutrient* OR milk OR multivitamin* OR nitrate* OR nitrite* OR nitrosamine* OR nut* OR nutrient* OR omega-3 OR n-3 OR omega-6 OR n-6 OR probiotic* OR seafood* OR starch* OR sugar* OR sucrose* OR supplement* OR sweeten* OR "trace element*" OR vegetable* OR vitamin* OR zinc OR yoghurt) NEAR/7 (iddm OR t1d OR t1dm)):ti,ab,kw  #6 (environment* NEAR/7 (diabetes OR islet OR insulin) NEAR/3 (autoantibod* OR autoimmun* OR brittle OR insulin-dependent OR juvenile OR "type 1")):ti,ab,kw #7 (environment* NEAR/7 (iddm OR t1d OR t1dm OR 'type 1')):ti,ab,kw #8 (("breast feed*" OR breastfeed*) NEAR/7 (diabetes OR islet OR insulin) NEAR/3 (autoantibod* OR autoimmun* OR brittle OR insulin-dependent OR juvenile OR "type 1")):ti,ab,kw #9 (("breast feed*" OR breastfeed*) NEAR/7 (iddm OR t1d OR t1dm OR "type 1")):ti,ab,kw #10 ((fetal OR infant OR maternal OR prenatal OR perinatal OR pregnancy) NEAR/3 (diet OR exposure* OR feed* OR food* OR intake* OR lactation OR nutrition*)):ti,ab,kw #11 ((fetal OR prenatal OR perinatal) NEAR/7 (iddm OR t1d OR t1dm OR "type 1")):ti,ab,kw #12 #4 or #5 or #6 or #7 or #8 or #9 or #10 or #11  #13 (aetiolog* OR develop* OR etiolog* OR inciden* OR pathogenes* OR prevent* OR protect* OR risk*):ti,ab,kw  #14 #3 and #12 and #13 limited to Cochrane Reviews and Trials | |

**Supplementary Table 4. Criteria for inclusion among studies using the same data**

| Higher priority  Lower priority | Lower risk of bias |
| --- | --- |
|  | Comparable cut-off/reference group with the other studies of the same association |
|  | Larger sample size |
|  | More recent year of publication |

**Supplementary Table 5. Dietary factors included in meta-analyses**

| Breastfeeding |
| --- |
| Infant formula |
| Solid food |
| Cow’s milk |
| Cereal |
| Gluten |
| Fiber |
| Meat |
| Egg |
| Fish |
| Fruit |
| Vegetables |
| Coffee |
| Sugar sweetened beverages |
| Sugar |
| Carbohydrate |
| Protein |
| Omega-3 fatty acids |
| Omega-6 fatty acids |
| Vitamin D |
| Vitamin A |
| Vitamin C |
| Iron |
| Nitrate |
| Nitrite |
| Nitrosamine |

**Supplementary Table 6. Characteristics of the studies that were included in the meta-analysis**

| **Study** | **Country** | **Cohort name** | **Study design** | **N total** | **N  cases** | **Age at diagnosis** | **Exposure*** | | **Exposure assessment** | **Time of exposure** | | **Outcome** | **Outcome assessment** | **IA^†^** | **HLA risk** | **Family history** | | | | **Adjusted factors** | **Risk of bias** | | |
| --- | --- | --- | --- | --- | --- | --- | --- | --- | --- | --- | --- | --- | --- | --- | --- | --- | --- | --- | --- | --- | --- | --- | --- |
| Ahadi  2011^1^ | Iran |  | Case-control | 202 | 101 | 6.7 ± 3.3 years | Vitamin D supplementation:  yes vs no | | Specific questionnaire | Infancy | | T1D | Clinical diagnosis | No | No | Some | | | | age, sex, maternal age at delivery, mode of delivery, type of feeding | Serious | | |
| Antvorskov 2018^2^ | Denmark | Danish National Birth Cohort (DNBC) | Cohort | 67565 | 247 | 9.5 ± 3.9 years | Gluten intake:  per 10 g/d | | FFQ | Fetal (week 25) | | T1D | Danish Childhood Diabetes Registry | No | No | No | | | | maternal body mass index before pregnancy, age, parity, smoking status, parental socioeconomic status, total energy intake, breastfeeding duration, caesarean section, offspring sex, pre-existing maternal type 2 diabetes and suspected gestational diabetes mellitus | Moderate | | |
| Ashraf  2010^3^ | USA |  | Case-control | 195 | 128 | 3.2 ± 1.3 years | Iron intake from formula, supplements, breastmilk:  per 10 mg/day | | Specific questionnaire | Infancy | | T1D | Hospital records | No | No | No | | | | birth weight, age at the time of the survey and birth order | Serious | | |
| Awadalla 2017^4^ | Egypt |  | Hospital-based case-control | 408 | 204 | 7.9 ± 3.4 years | Introduction of vitamin D supplements: yes vs no | | Specific questionnaire | Infancy (1st year) | | T1D | Clinical diagnosis: WHO criteria | No | No | No | | | | age, sex, residence (rural vs. urban), parental history of T1D, mode of delivery, breastfeeding duration/introduction of cow's milk/vitamin D supplementation, physical activity | Serious | | |
|  |  |  |  |  |  |  | Breastfeeding duration:  > 6 months vs < 6 months | |  |  |  |  |  |  |  |  |  |  |  |  |  |  |  |
|  |  |  |  |  |  |  | Age at cow's milk introduction:  ≥ 1 year vs < 1 year | |  |  |  |  |  |  |  |  |  |  |  |  |  |  |  |
|  |  |  |  |  |  |  | Meat intake: > 4 times/week vs < 4 times/week | |  | Childhood | |  |  |  |  |  |  |  |  | age, sex |  |  |  |
|  |  |  |  |  |  |  | Fish intake: > 4 times/week vs < 4 times/week | |  |  |  |  |  |  |  |  |  |  |  |  |  |  |  |
| Bener  2009^5^ | Qatar |  | Case-control | 340 | 170 | < 16 years | Breastfeeding duration:  ≥ 6 months vs < 6 months | | Specific questionnaire | Infancy | | T1D | Clinical diagnosis | No | No | Some | | | | age, gender, ethnicity, father occupation, mother occupation, physical activity, family history of diabetes, low duration of time under sun light, low vitamin D level | Serious | | |
| Benson  2008^6^ | Canada |  | Case-control | 162 | 57 | 9 (4-13) years | Sugar sweetened beverage intake: > 1 times/week vs < 1 times/week | | FFQ | Year before diagnosis/ interview | | T1D | PEI Diabetes registry:  Canadian Diabetes Association criteria | No | No | Some | | | | age, sex, type of FFQ | Serious | | |
|  |  |  |  |  |  |  | Egg intake: per 50 g/day | |  |  |  |  |  |  |  |  |  |  |  | age, sex, type of FFQ, family history of T1D, infections during childhood, father's education, residence |  |  |  |
|  |  |  |  |  |  |  | Orange juice intake:  per 250 g/day | |  |  |  |  |  |  |  |  |  |  |  | age, sex, type of FFQ |  |  |  |
| Benson  2010^7^ | Canada |  | Case-control | 159 | 55 | <18 years | Iron intake:  per 10 mg/day | | FFQ | Year before diagnosis/ interview | | T1D | PEI Diabetes registry:  Canadian Diabetes Association criteria | No | No | No | | | | age, sex, and energy intake (Kcal), a third-generation family member previously diagnosed with T1D (siblings, parents, grandparents, parental siblings, and cousins), the number of infections during the first 2 years of life, residential area (urban, rural or farm), and father’s education level | Serious | | |
|  |  |  |  |  |  |  | Carbohydrate intake:   ≥ 352 g/day vs < 233.93 g/day | |  |  |  |  |  |  |  |  |  |  |  |  |  |  |  |
|  |  |  |  |  |  |  | Total sugars intake:  ≥ 171.42 g/day vs < 120.57 g/day | |  |  |  |  |  |  |  |  |  |  |  |  |  |  |  |
|  |  |  |  |  |  |  | Caffeine intake: ≥ 22 microg/day vs < 3.36 microg/d | |  |  |  |  |  |  |  |  |  |  |  |  |  |  |  |
|  |  |  |  |  |  |  | Fiber intake:  ≥ 18.6 g/day vs < 11.5 g/d | |  |  |  |  |  |  |  |  |  |  |  |  |  |  |  |
|  |  |  |  |  |  |  | Vitamin C intake:  ≥ 203.90 mg/day vs < 81.20 mg/day | |  |  |  |  |  |  |  |  |  |  |  |  |  |  |  |
|  |  |  |  |  |  |  | Vitamin A intake:  ≥ 952 microg/day vs < 501.8 microg/day | |  |  |  |  |  |  |  |  |  |  |  |  |  |  |  |
|  |  |  |  |  |  |  | Nitrate intake:  ≥ 9.01 mg/day vs < 5.66 mg/day | |  |  |  |  |  |  |  |  |  |  |  |  |  |  |  |
|  |  |  |  |  |  |  | Nitrite intake:  ≥ 4.82 mg/day vs < 1.83 mg/day | |  |  |  |  |  |  |  |  |  |  |  |  |  |  |  |
|  |  |  |  |  |  |  | Nitrosamine intake:  ≥ 0.04 microg/day vs < 0.01 microg/day | |  |  |  |  |  |  |  |  |  |  |  |  |  |  |  |
| Beyerlein 2015^8^ | USA and Germany | The Environmental Determinants  of Diabetes in the Young (TEDDY) | Cohort | 3330 | 70 | < 5 years | Soluble fiber intake:  per 10g increase | | 3-day food record | 1 year old | | T1D | Clinical diagnosis: American Diabetes Association criteria | No | Some | Some | | | | sex, human leukocyte antigen–DR3/DR4 status, country, and having a first-degree relative with type 1 diabetes, birth order, maternal BMI, delivery mode, maternal smoking in pregnancy, maternal education, and duration of exclusive breastfeeding or breastfeeding status at the time of the diet record | Moderate | | |
|  |  |  |  | 3252 | 207 |  |  |  |  |  |  | IA | Lab measurement (no details) |  |  |  |  |  |  |  |  |  |  |
| Bodington 1994^9^ | UK |  | Case-control | 356 | 178 | < 15 | Breastfeeding:  any vs none | | Questionnaire | Infancy | | T1D | Clinical diagnosis | No | No | No | | | | age, sex, race | Serious | | |
|  |  |  |  |  |  |  | Exclusive breastfeeding duration:  > 3 months vs ≤ 3 months | |  |  |  |  |  |  |  |  |  |  |  |  |  |  |  |
| Brekke  2007^10^ | Sweden | All Babies in Southeast Sweden (ABIS) | Cohort | 8694 | 774 | 1 year | Vitamin D supplementation:  yes vs no | | Specific questionnaire | Fetal | | IA | Immunoprecipitation method | No | No | No | | | | familial type 1 diabetes, maternal education, maternal age, delivery mode, weight increase from birth, breast‐feeding duration, introduction of cow’s‐milk protein, fish intake | Moderate | | |
| Brekke  2010^11^ | Sweden | All Babies in Southeast Sweden (ABIS) | Cohort | 5724 | 191 | < 5 years | Dairy intake (Including milk/Yoghurt/Sour milk): per 400 g/day | | FFQ | Fetal | | IA | Immunoprecipitation method/radio-binding assay for IAA | No | No | No | | | | maternal education, weight increase from birth to 2.5 yr, breastfeeding duration, and introduction of cow’s-milk protein | Moderate | | |
|  |  |  |  |  |  |  | Fish intake (open sea):  per 100 g/day | |  |  |  |  |  |  |  |  |  |  |  |  |  |  |  |
|  |  |  |  |  |  |  | Vegetable intake:  per 100 g/day | |  |  |  |  |  |  |  |  |  |  |  |  |  |  |  |
|  |  |  |  |  |  |  | Root vegetable intake: per 100 g/day | |  |  |  |  |  |  |  |  |  |  |  |  |  |  |  |
|  |  |  |  |  |  |  | Meat intake (cow, calf, ox):  per 100 g/day | |  |  |  |  |  |  |  |  |  |  |  |  |  |  |  |
|  |  |  |  |  |  |  | Egg intake: per 50 g/day | |  |  |  |  |  |  |  |  |  |  |  |  |  |  |  |
|  |  |  |  |  |  |  | Coffee intake: daily vs never | |  |  |  |  |  |  |  |  |  |  |  |  |  |  |  |
| Cadario 2015^12^ | Italy |  | Case-control | 303 | 67 | 4.2 years (1.5-6.9) | Vitamin D blood levels:  per 10 nmol/L | | Tandem mass spectroscopy (LC–MS/MS) | At birth | | T1D | Piedmont Childhood Diabetes registry | No | No | No | | | | age, place of birth, ethnic group | Serious | | |
| Chmiel 2015^13^ | Germany | BABYDIAB BABYDIET | Cohort | 2291 | 82 | < 15 years | Age at gluten introduction: > 6 months vs 3.1-6 months  3.1-6 months vs < 3 months | | Questionnaire, food records, interviews | Infancy (<3 months) | | T1D | Clinical diagnosis: American Diabetes Association criteria | No | No | Yes | | | | maternal type 1 diabetes, maternal age at birth, preterm delivery (<37 weeks), low birth weight, and study cohort (BABYDIAB/BABYDIET) | Moderate | | |
|  |  |  |  |  | 125 |  |  |  |  |  |  | IA | Lab measurement (no details) |  |  |  |  |  |  |  |  |  |  |
| Dahlquist 1991^14^ | Sweden | The Swedish childhood diabetes study | Case-control | 867 | 339 | < 14 years | Breastfeeding duration:  ≥ 3 months vs < 3 months | | Questionnaire | Infancy | | T1D | Stockholm diabetes register | No | No | Some | | | | age, sex, county, familial type 1 diabetes, familial type 2 diabetes, maternal age ≥ 40), maternal education (< university), frequency of intake of milk, foods rich in protein, carbohydrate, nitrosamines, no measles vaccination, frequency of infections last year (> 1), increased height, stressful life events last year (> 0) | Serious | | |
|  |  |  |  |  |  |  | Intake frequency of foods rich in carbohydrate:  > 25th centile vs < 25th centile | |  | Child | |  |  |  |  |  |  |  |  | age, sex, county, familial type 1 diabetes, familial type 2 diabetes, maternal age ≥ 40), maternal education (< university), frequency of intake of milk, breastfeeding duration, protein, nitrosamines, no measles vaccination, frequency of infections last year (> 1), increased height, stressful life events last year (> 0) |  |  |  |
|  |  |  |  |  |  |  | Intake frequency of foods rich in protein:  > 25th centile vs < 25th centile | |  |  |  |  |  |  |  |  |  |  |  | age, sex, county, familial type 1 diabetes, familial type 2 diabetes, maternal age ≥ 40), maternal education (< university), frequency of intake of milk, breastfeeding duration, carbohydrate, nitrosamines, no measles vaccination, frequency of infections last year (> 1), increased height, stressful life events last year (> 0) |  |  |  |
|  |  |  |  |  |  |  | Intake frequency of foods rich in nitrosamines:  >25th centile vs <25th centile | |  |  |  |  |  |  |  |  |  |  |  | age, sex, county, familial type 1 diabetes, familial type 2 diabetes, maternal age ≥ 40), maternal education (< university), frequency of intake of milk, foods rich in protein, carbohydrate, breastfeeding duration, no measles vaccination, frequency of infections last year (> 1), increased height, stressful life events last year (> 0) |  |  |  |
| Dahlquist 1992^15^ | Sweden |  | Case-control | 228 | 116 | 8.7 ± 4.0 years | Age at infant formula introduction:  ≥ 4 months vs < 4 months | | Questionnaire | Infancy | | T1D | Swedish diabetes register | Some | No | No | | | | age, sex, cow's milk antibodies, islet cell antibodies | Serious | | |
| EURODIAB 1999^16^ | Austria Bucharest Bulgaria Latvia Lithuania Luxembourg  N. Ireland | EURODIAB | Case-control | 2936 | 746 | < 15 years | Vitamin D supplementation:  yes vs no | | Specific questionnaire/Interview | Infancy | | T1D | Diabetes registers | No | No | No | | | | Age, duration of breast feeding less than 3 months, maternal age over 35 years, birth weight less than 2500 g, and study centre | Serious | | |
| Frederiksen 2013^17^ | USA | The Diabetes Autoimmunity Study in the Young (DAISY) | Cohort | 1835 | 53 | < 18 years | Exclusive breastfeeding duration:  per additional month | | Telephone or face to face interview | Infancy | | T1D | Clinical diagnosis | No | Some | Some | | | | human leukocyte antigen genotype, first-degree relative with T1DM, maternal education, and delivery type | Moderate | | |
|  |  |  |  |  |  |  | Age at cereal introduction (wheat/barley/oats/rice): ≥ 6 months vs 4 or 5 months  4 or 5 months vs < 4 months | |  |  |  |  |  |  |  |  |  |  |  |  |  |  |  |
|  |  |  |  |  |  |  | Age at gluten containing cereal introduction (wheat/barley): ≥ 6 months vs 4 or 5 months 4 or 5 months vs < 4 months | |  |  |  |  |  |  |  |  |  |  |  |  |  |  |  |
|  |  |  |  |  |  |  | Age at solid food introduction:  4 or 5 months vs < 4 months | |  |  |  |  |  |  |  |  |  |  |  |  |  |  |  |
|  |  |  |  |  |  |  | Age at fruit introduction (no fruit juice):  ≥ 6 months vs 4 or 5 months 4 or 5 months vs < 4 months | |  |  |  |  |  |  |  |  |  |  |  |  |  |  |  |
|  |  |  |  |  |  |  | Age at vegetable introduction: ≥ 6 months vs 4 or 5 months  4 or 5 months vs < 4 months | |  |  |  |  |  |  |  |  |  |  |  |  |  |  |  |
|  |  |  |  |  |  |  | Age at meat introduction: 4 or 5 months vs < 4 months | |  |  |  |  |  |  |  |  |  |  |  |  |  |  |  |
| Gimeno 1997^18^ | Brazil |  | Case-control | 692 | 346 | ≤ 18 years | Exclusive breastfeeding duration:  > 60 days vs ≤ 60 days | | Specific questionnaire | Infancy | | T1D | Medical history | No | No | Some | | | | age, sex, neighborhood | Serious | | |
|  |  |  |  |  |  |  | Age at introduction to cow's milk products:  ≥ 3 months vs < 3 months | |  |  |  |  |  |  |  |  |  |  |  |  |  |  |  |
| Glatthaar 1988^19^ | Australia |  | Case-control | 947 | 194 | < 18 years | Use of vitamin C tablets for more than a month:  yes vs no | | Questionnaire | Prior to diagnosis | | T1D | Self-reported, cross-checked with health records | No | No | Some | | | | age, sex | Serious | | |
| Gorham 2012^20^ | USA |  | Nested case-control | 2000 | 1000 | > 17 years (military) | Serum 25-OH D levels:  per 10 nmol/l | | competitive chemiluminescence immunoas say | Adult | | T1D | Military care database | No | No | No | | | | race, date that the blood sample was drawn (±2 days), age (±3 months), length of military service (±30 days), sex and whether the control was on active duty when the case was diagnosed | Moderate | | |
| Granfors 2016^21^ | Sweden | All Babies in Southeast Sweden (ABIS) | Cohort | 7138 | 47 | < 16 years | Vitamin D supplementation from multivitamins:  ≥2 µg vitamin D/d vs none | | FFQ | Fetal | | T1D | Swediabkids national register | No | No | No | | | | parity (siblings yes/no), mother's education level, maternal age, and smoking during pregnancy, father's education level, maternal infections during pregnancy, mode of delivery, familial type 1 diabetes, child's gender, mother's consumption of fish during pregnancy (as source of vitamin D), duration of exclusive breastfeeding, month of introduction to cow's milk, month of introduction to gluten, and AD‐vitamin droplets given to the child during the child's first year | Moderate | | |
| Hakola  2019^22^ | Finland | Finnish Type 1 Diabetes Prediction and Prevention (DIPP) | Cohort | 5714 | 90 | < 6 years | Gluten intake:  per 10 g increase | | 3-day food record | < 6 years old | | T1D | Finnish Pediatric Diabetes Register | No | Yes | Some | | | | sex of the child, human leukocyte antigen genotype, and familial  diabetes | Moderate | | |
|  |  |  |  |  |  |  | Fiber intake:  per 10 g increase | |  |  |  |  |  |  |  |  |  |  |  |  |  |  |  |
|  |  |  |  | 5545 | 246 |  | Gluten intake:  per 10 g increase | |  |  |  | IA | Radiobinding assays |  |  |  |  |  |  |  |  |  |  |
|  |  |  |  |  |  |  | Fiber intake:  per 10 g increase | |  |  |  |  |  |  |  |  |  |  |  |  |  |  |  |
| Hall  2015^23^ | USA | The Diabetes Autoimmunity Study in the Young (DAISY) | Cohort | 1783 | 58 | 8.5 ± 3.8 years | Breastfeeding duration: per additional month | | Specific questionnaire | Infancy | | T1D | Clinical diagnosis | No | Some | Some | | | | HLA, first degree relative with T1D, and ethnicity, attending daycare during the first 2 years of life | Moderate | | |
| Hummel 2017^24^ | USA and Europe | The Environmental Determinants  of Diabetes in the Young (TEDDY) | Cohort | 8506 | 686 | < 8 years | First formula introduced during first 3 months:  Cow’s milk–based, nonhydrolyzed vs no formula, no cow's milk | | Specific questionnaire | Infancy (3 months) | | IA | Lab measurement (no details) | No | Yes | Some | | | | Adjusted for HLA genotype, first-degree relative with type 1 diabetes, mother with type 1 diabetes, sex, country, mode of delivery, any breastfeeding 3 months, and seasonality of birth | Moderate | | |
| Hyppönen 1999^25^ | Finland | Childhood Diabetes in Finland  (DiMe) | Case-control | 821 | 435 | 8.2 ± 3.6 years | Age at cow's milk formula introduction:  ≥ 3 months vs < 3 months | | Specific questionnaire | Infancy (3 months) | | T1D | Diabetes registry | No | No | No | | | | age, sex, weight at the age of exposure | Serious | | |
| Hyppönen 2001^26^ | Finland |  | Cohort | 10366 | 81 | 14 (1-31) years | Vitamin D supplementation:  regularly vs none | | Specific questionnaire | Infancy | | T1D | Drug register  &  Hospital discharge registers | No | No | No | | | | sex, neonatal (parity, gestational and maternal age), length of maternal education, social status, and standardized birth weight, and growth rate in infancy (suspected rickets adjusted in addition to the increased dose of vitamin D) | Moderate | | |
| Jacobsen 2016^27^ | Denmark |  | Case-control | 858 | 429 | 8.5 years | Serum 25(OH)D3 levels:   per 10 nmol/L | | liquid chromatography–tandem mass spectrometry | At birth | | T1D | Danish Childhood Diabetes Registry & Hospital records | No | No | Some | | | | date of birth, gestational and mother’s delivery age, birthweight, mother’s ethnicity, HLA risk | Moderate | | |
| Jones 1998^28^ | UK | Oxford Record Linkage Study | Case-control | 518 | 60 | < 20 years | Breastfeeding at discharge from hospital:  yes vs no | | hospital records | Infancy (1st week) | | T1D | Hospital records | No | No | No | | | | sex, year and hospital of delivery | Serious | | |
| Koivusaari 2020^29^ | Finland | Finnish Type 1 Diabetes Prediction and Prevention (DIPP) | Cohort | 5545 | 246 | 2.5 years (1.3- 3.6) | Intake of non-fermented milk products, fermented products, cheeses:  per 400g | | 3-day food records | < 6 years | | IA | Islet cell autoantibodies (ICAs) with immunofluorescence | No | Yes | Some | | | | child's sex, genetic risk and first-degree familial diabetes | Moderate | | |
| Kostraba 1992^30^ | USA |  | Case-control | 422 | 211 | < 17 years | Age at cow's milk introduction: ≥ 3 months vs < 3 months | | Specific question | Infancy | | T1D | Allegheny County & Children's Hospital of Pittsburgh  IDDM Registries | No | No | No | | | | birth order, birth year (±2 yr), and race, breast-feeding status and maternal age at birth | Serious | | |
|  |  |  |  |  |  |  | Age at infant formula introduction:  ≥ 3 months vs < 3 months | |  |  |  |  |  |  |  |  |  |  |  |  |  |  |  |
| Kostraba 1993^31^ | USA |  | Case-control | 259 | 142 | < 18 years | Age at solid food introduction: ≥ 3 months vs < 3 months | | Questionnaire | Infancy | | T1D | Colorado IDDM Registry | No | Some | No | | | | age, sex, ethnicity, birth order, family income | Serious | | |
| Kyvik 1992^32^ | Denmark |  | Nested case-control | 230 males | 76 | < 20 years | Breastfeeding duration:  ≥ 3 months vs < 3 months | | Health records | Infancy | | T1D | National Service Conscript Registry | No | No | No | | | | age, restricted to males | Serious | | |
|  |  |  |  |  |  |  | Breastfeeding: any vs none | |  |  |  |  |  |  |  |  |  |  |  |  |  |  |  |
| Lamb  2008a^33^ | USA | The Diabetes Autoimmunity Study in the Young (DAISY) | Cohort | 642 | 27 | 4 years | Breastfeeding duration:  per additional month | | Telephone interview | Infancy | | IA | Radioimmunoassays | No | Some | Some | | | | total calories of the maternal diet, ethnicity, human leukocyte antigen genotype of the child, and whether or not the child has a first-degree relative with type 1 diabetes mellitus | Moderate | | |
|  |  |  |  |  |  |  | Cow's milk products intake:  per 1 SD increase | | semi-quantitative FFQ | Fetal (3d trimester) | |  |  |  |  |  |  |  |  |  |  |  |  |
|  |  |  |  |  |  |  | Intake of gluten containing foods: per 1 SD increase | |  |  |  |  |  |  |  |  |  |  |  |  |  |  |  |
|  |  |  |  |  |  |  | Fish intake (canned tuna, dark-meat fish, other fish, and shrimp, lobster, or scallops:  per 100 g/day | |  |  |  |  |  |  |  |  |  |  |  |  |  |  |  |
|  |  |  |  |  |  |  | Meat intake (chicken, turkey processed meats, liver, beef, pork, lamb: per 100 g/day | |  |  |  |  |  |  |  |  |  |  |  |  |  |  |  |
|  |  |  |  |  |  |  | Vegetable intake: per 100 g/day | |  |  |  |  |  |  |  |  |  |  |  |  |  |  |  |
|  |  |  |  |  |  |  | Root vegetable intake (beets, cooked carrots, raw carrots, and yams or sweet potatoes.):  per 100 g/day | |  |  |  |  |  |  |  |  |  |  |  |  |  |  |  |
|  |  |  |  |  |  |  | Fruit intake: per 100 g/day | |  |  |  |  |  |  |  |  |  |  |  |  |  |  |  |
| Lamb  2008b^34^ | USA | The Diabetes Autoimmunity Study in the Young (DAISY) | Cohort | 89 | 17 | 6.2 ± 2.5 years | Fiber intake:  per 10g increase | | semi-quantitative FFQ | Annually after the age of 2 (Diet for the year before diagnosis) | | T1D | Clinical diagnosis | Yes | Some | Some | | | | total calories, HLA genotype, age at first autoantibody positive visit, and family history of type 1 diabetes | Moderate | | |
|  |  |  |  | 1776 | 79 | 4.8 ± 2.5 |  |  |  |  |  | IA | Radioimmunoassays |  |  |  |  |  |  | total calories, HLA genotype, and family history of type 1 diabetes |  |  |  |
| Lamb  2015a^35^ | USA | The Diabetes Autoimmunity Study in the Young (DAISY) | Cohort | 142 | 42 | 10.9 years (7.0-17.2) | Carbohydrate intake:  per 1 SD increase | | semi-quantitative FFQ | Annually after the age of 2 (Diet for the year before diagnosis) | | T1D | Clinical diagnosis | Yes | Some | Some | | | | age at first autoantibody positivity, total energy, FFQ type, HLA-DR, DQ genotype, family history of type 1 diabetes, ethnicity | Moderate | | |
|  |  |  |  |  |  |  | Sugar intake:  per 1 SD increase | |  |  |  |  |  |  |  |  |  |  |  |  |  |  |  |
|  |  |  |  |  |  |  | Juice intake:  per 1 serving/day | |  |  |  |  |  |  |  |  |  |  |  |  |  |  |  |
|  |  |  |  |  |  |  | Sugar sweetened beverages intake: per 2 servings/week | |  |  |  |  |  |  |  |  |  |  |  | age at first autoantibody positivity, total energy, FFQ type, family history of type 1 diabetes, ethnicity |  |  |  |
| Lamb  2015b^36^ | USA | The Diabetes Autoimmunity Study in the Young (DAISY) | Cohort | 143 | 40 | 9.6 ± 3.5 years | Cow's milk protein intake:  per 10 g/day | | semi-quantitative FFQ | Annually after the age of 2 (Diet for the year before diagnosis) | | T1D | Clinical diagnosis |  |  |  | | | | total caloric intake, FFQ type, HLA-DR status, family history of T1D, age at first IA positivity and ethnicity | Moderate | | |
|  |  |  |  | 1835 | 133 | 7.0 ± 3.9 years |  |  |  |  |  | IA | Radioimmunoassays |  |  |  |  |  |  | total caloric intake, FFQ type, family history of T1D, and ethnicity, HLA genotype |  |  |  |
| Lund-Blix 2015^37^ | Norway | Environmental Triggers of Type 1 Diabetes (MIDIA) | Cohort | 726 | 56/25 | < 18 years | Breastfeeding duration: per additional month | | Specific questionnaire | Infancy | | IA | Radio-binding assays | Some | Yes | Some | | | | first-degree relative with type 1 diabetes, vitamin D supplementation, maternal education level, sex, and delivery type | Moderate | | |
|  |  |  |  |  |  |  | Exclusive breastfeeding duration: per additional month | |  |  |  | IA/T1D | Clinical diagnosis/  Radio-binding assays |  |  |  |  |  |  |  |  |  |  |
|  |  |  |  |  |  |  | Age at gluten introduction: ≥ 6 months vs 5-5.9 months 5-5.9 months vs < 5 months | |  |  |  |  |  |  |  |  |  |  |  |  |  |  |  |
|  |  |  |  |  |  |  | Age at infant formula introduction: per month of introduction delay | |  |  |  |  |  |  |  |  |  |  |  |  |  |  |  |
|  |  |  |  |  |  |  | Age at cereal introduction: ≥ 6 months vs 5-5.9 months 5-5.9 months vs < 5 months | |  |  |  |  |  |  |  |  |  |  |  |  |  |  |  |
|  |  |  |  |  |  |  | Age at fruit introduction: ≥ 6 months vs 5-5.9 months 5-5.9 months vs < 5 months | |  |  |  |  |  |  |  |  |  |  |  |  |  |  |  |
|  |  |  |  |  |  |  | Age at vegetable introduction: per 1 month delay | |  |  |  |  |  |  |  |  |  |  |  |  |  |  |  |
|  |  |  |  |  |  |  | Age at fish introduction:  per 1 month delay | |  |  |  |  |  |  |  |  |  |  |  |  |  |  |  |
|  |  |  |  |  |  |  | Age at meat introduction: 8-8.9 months vs < 8 months vs | |  |  |  |  |  |  |  |  |  |  |  |  |  |  |  |
|  |  |  |  |  |  |  | Age at solid food introduction: 5-5.9 months vs < 5 months | |  |  |  |  |  |  |  |  |  |  |  |  |  |  |  |
|  |  |  |  |  |  |  | Breastfeeding duration:  ≥ 12 months vs < 12 months | |  |  |  | T1D | Clinical diagnosis |  |  |  |  |  |  |  |  |  |  |
| Lund-Blix 2017^38^ | Denmark | Danish National Birth Cohort  (DNBC) | Cohort | 66676 | 219 | 8.5 years (0.9-15.8) | Breastfeeding duration: per additional month | | Telephone interview | Infancy | | T1D | Danish  Childhood Diabetes Registry | No | No | No | | | | gender, birth weight, gestational age, caesarean section, parity, maternal smoking in pregnancy, maternal age, maternal BMI, and parental diabetes | Moderate | | |
|  |  |  |  |  |  |  | Exclusive breastfeeding duration: per additional month | |  |  |  |  |  |  |  |  |  |  |  |  |  |  |  |
|  |  |  |  |  |  |  | Age at infant formula introduction: per month of introduction delay | |  |  |  |  |  |  |  |  |  |  |  |  |  |  |  |
|  |  |  |  |  |  |  | Age at solid food introduction: ≥ 6 months vs < 4 months | |  |  |  |  |  |  |  |  |  |  |  |  |  |  |  |
|  | Norway | Mother and Child Cohort Study  (MoBa) | Cohort | 88716 | 285 | 6.7 years (0.7-15.9) | Breastfeeding duration:  per additional month | | Specific questionnaire | Infancy | | T1D | Norwegian Childhood Diabetes  Registry  &  Norwegian Patient  Registry | No | No | No | | | |  |  |  |  |
|  |  |  |  |  |  |  | Exclusive breastfeeding duration: per additional month | |  |  |  |  |  |  |  |  |  |  |  |  |  |  |  |
|  |  |  |  |  |  |  | Age at infant formula introduction: per month of introduction delay | |  |  |  |  |  |  |  |  |  |  |  |  |  |  |  |
|  |  |  |  |  |  |  | Age at solid food introduction: ≥ 6 months vs < 4 months | |  |  |  |  |  |  |  |  |  |  |  |  |  |  |  |
| Lund-Blix 2019^39^ | USA | The Diabetes Autoimmunity Study in the Young (DAISY) | Cohort | 129 | 42 | < 15 years | Gluten intake:  per 10g/day | | semi-quantitative FFQ | Infancy  (1-2 years) | | T1D | Clinical diagnosis | Yes | Some | Some | | | | child’s total energy intake, age at introduction of gluten, family history of CD, HLA genotype, family history of T1D, parent-reported race-ethnicity, sex, and maternal age at the time of delivery | Moderate | | |
|  |  |  |  | 1916 | 178 | < 15 years |  |  |  | Year before diagnosis | | IA | Radioimmunoassays |  |  |  |  |  |  | child’s total energy intake, age at introduction of gluten, family history of CD, appearance of CDA, HLA genotype, family history of T1D, type of FFQ, parent-reported race-ethnicity, sex, and maternal age at delivery |  |  |  |
|  |  |  |  |  |  |  | Age at gluten introduction: ≥ 6 months vs 4-5.9 months 4-5.9 months vs < 4 months | | Telephone or face to face interview | Infancy | |  |  |  |  |  |  |  |  | child’s total energy intake, gluten intake at age 1–2 years, family history of CD, HLA genotype, family history of T1D, parent-reported race-ethnicity, sex, and maternal age at the time of delivery |  |  |  |
| Lund-Blix 2020^40^ | Norway | Mother and Child Cohort Study  (MoBa) | Cohort | 86306 | 346 | 7.5 years (0.7-15.0) | Fiber intake: per 10g/d increase | | semi-quantitative FFQ | Fetal (week 22) | | T1D | Norwegian Childhood Diabetes  Registry  &  Norwegian Patient  Registry | No | No | Some | | | | maternal age, prepregnant maternal BMI, parity, smoking during pregnancy, education, cesarean section, breastfeeding, sex, energy intake, birth weight, age at gluten introduction, prematurity, fiber intake, weight gain 0–12 months |  |  |  |
|  |  |  |  |  |  |  | Gluten intake: per 10g/d increase | |  |  |  |  |  |  |  |  |  |  |  | maternal age, prepregnant maternal BMI, parity, smoking during pregnency, education, cesarean section, breastfeeding, sex, energy intake, birth weight, age at gluten introduction, prematurity, fibre intake, weight gain 0-12 months, child's gluten intake/mother’s gluten intake, coeliac disease |  |  |  |
|  |  |  |  | 66725 | 271 |  |  |  | Specific questionnaire | 18 months | |  |  |  |  |  |  |  |  |  |  |  |  |
| Malcova 2006^41^ | Czech Republic |  | Case-control | 2084 | 738 | < 15 years | Breastfeeding duration: > 4-6 months vs 1-3 months | | Specific questionnaire | Infancy | |  | Diabetes register | No | No | No | | | | time at introduction of formula feeding, the maternal age and birth order, and for the year of birth | Serious | | |
|  |  |  |  | 2084 | 738 |  | Breastfeeding duration: 1-3 months vs no | |  |  |  |  |  |  |  |  |  |  |  |  |  |  |  |
|  |  |  |  | 2157 | 754 |  | Age at infant formula introduction:  4-6 months vs 1-3 months | |  |  |  |  |  |  |  |  |  |  |  | duration of breast-feeding, the maternal age and birth order, and for the year of birth |  |  |  |
| Marshall 2004^42^ | UK |  | Case-control | 577 | 196 | < 16 years | Breastfeeding duration:  per additional month | | structured interview | Infancy | | T1D | Hospital records | No | No | Some | | | | age, sex | Serious | | |
|  |  |  |  |  |  |  | Breastfeeding:  yes vs no | |  |  |  |  |  |  |  |  |  |  |  |  |  |  |  |
|  |  |  |  |  |  |  | Age at infant formula introduction: per month of introduction delay | |  | Infancy | |  |  |  |  |  |  |  |  | age, sex, room sharing, social contact between 6 and 11 months, parental diabetes, maternal thyroid, consumption of sugary food, maternal smoking during pregnancy, father's qualifications, age at introduction to formula milk, mother's age at birth, other infections in pregnancy, illnesses or conditions in pregnancy, race, typhoid vaccination, regular contact with animals |  |  |  |
| Mattila 2020b^43^ | Finland | Finnish Type 1 Diabetes Prediction and Prevention (DIPP) | Cohort | 4757 | 174 | 7.1 years (4.3-10.6) | Nitrate intake: per 1 SD (97 mg/d) | | semi-quantitative FFQ | Fetal (8th month) | | T1D | Clinical diagnosis | Some | Yes | Some | | | | energy with residual method, sex, family history of diabetes, human leukocyte antigen genotype, vitamin C, vitamin E, and selenium intakes | Moderate | | |
|  |  |  |  |  |  |  | Nitrite intake  per 1 SD (1 mg/d) | |  |  |  |  |  |  |  |  |  |  |  |  |  |  |  |
| Mayer 1988^44^ | USA |  | Case-control | 747 | 268 | < 18 years | Breastfeeding duration: 1-3 months vs none | | no detailed information (self-reported) | Infancy | | T1D | Colorado IDDM Registry  &  Barbara Davis Center for  Childhood Diabetes | No | No | No | | | | birth year, race, income, maternal education, maternal age, and sex | Serious | | |
| McKinney 1999^45^ | UK |  | Case-control | 521 | 196 | < 16 years | Initial exclusive breastfeeding: yes vs no | | face to face interview | Infancy | | T1D | Yorkshire Childhood Diabetes Register | No | No | Some | | | | age, sex, mother's age, mother with T1D, preeclampsia, cesarean delivery, neonatal illnesses | Serious | | |
| Meloni  1997^46^ | Italy |  | Case-control | 200 | 100 | < 17 years | Breastfeeding duration: per additional month | | Specific questionnaire | Infancy | | T1D | Hospital records | No | No | No | | | | age, sex | Serious | | |
|  |  |  |  |  |  |  | Breastfeeding: any vs none | |  |  |  |  |  |  |  |  |  |  |  |  |  |  |  |
|  |  |  |  |  |  |  | Age at cow's milk introduction: > 6 months vs < 3 months | |  |  |  |  |  |  |  |  |  |  |  |  |  |  |  |
|  |  |  |  |  |  |  | Age at solid food introduction:  > 6 months vs < 3 months | |  |  |  |  |  |  |  |  |  |  |  |  |  |  |  |
| Miettinen 2020^47^ | Europe, USA, Canada, Australia | Trial to Reduce IDDM In the Genetically at Risk (TRIGR) | Nested case-control | 432 | 144 | 6 years (2.9-9.4) | Serum 25(OH)D levels: per 10 nmol/l | | chemiluminescent microparticle immunoas say | At birth | | T1D | Clinical diagnosis | No | Yes | Yes | | | | age, country, month of sample collection, HLA genotype, maternal type 1 diabetes and sex | Moderate | | |
|  |  |  |  |  |  |  |  |  |  | Child | |  |  |  |  |  |  |  |  |  |  |  |  |
|  |  |  |  | 732 | 244 | 2 years (1.0-4.0) |  |  |  | Infancy (6 months) | | IA | Radiobinding assays |  |  |  |  |  |  |  |  |  |  |
| Munger 2013^48^ | USA |  | Nested case-control | 360 | 180 | 26 ± 5.4 years | Serum 25(OH)D levels: per 10 nmol/l | | chemiluminescence immunoassay (CLIA) | Adult | | T1D | US Naval Council of  Personnel Boards database  &  Medical records | No | No | No | | | | matching factors (age, race, date of blood collection, sex, and branch of military service) and latitude of residence at entry into the military | Serious | | |
| Muntoni 2013^49^ | Italy |  | Hospital-based case-control | 250 | 123 | 6.3 years | Exclusive breastfeeding duration: ≥ 3 months vs < 3 months | | semi-quantitative FFQ | Infancy | | T1D | Clinical diagnosis: American  Diabetes Association guidelines | No | No | No | | | | gender, child food item consumption during and after the first 2 years of life, consumptions of the mother during pregnancy and lactation | Serious | | |
|  |  |  |  |  |  |  | Meat intake:  almost daily vs 1-3 times a month | |  | First 2 years of life | |  |  |  |  |  |  |  |  | gender, breastfeeding, child food item consumption during and after the first 2 years of life, consumptions of the mother during pregnancy and lactation |  |  |  |
| Niinistö 2014^50^ | Finland | Finnish  Type 1 Diabetes Prediction and Prevention (DIPP) | Cohort | 4887 | 240 | 3.6 years (0.5-10) | Cow's milk products intake: per 1 SD (422g/d) | | semi-quantitative FFQ | Fetal (8th month) | | IA | Immunofluorescence, microassay, radiobibding assays | Some | Yes | Some | | | | maternal energy intake, genetic risk, hospital of birth, familial diabetes and maternal vocational education | Moderate | | |
|  |  |  |  |  |  |  | Fatty fish intake:  per 100 g/day | |  |  |  |  |  |  |  |  |  |  |  |  |  |  |  |
|  |  |  |  |  |  |  | Red meat intake: 100 g/day | |  |  |  |  |  |  |  |  |  |  |  |  |  |  |  |
|  |  |  |  |  | 112 | 4.6 years (0.5-11.5) | Omega-3 intake: per 1 SD | |  |  |  | T1D | Clinical diagnosis |  |  |  |  |  |  | genetic risk, hospital of birth, familial diabetes and maternal vocational education |  |  |  |
|  |  |  |  |  |  |  | Omega-6 intake: per 1 SD | |  |  |  |  |  |  |  |  |  |  |  |  |  |  |  |
|  |  |  |  |  | 240 | 3.6 years (0.5-10) | Omega-3 intake: per 1 SD | |  |  |  | IA | Immunofluorescence, microassay, radiobibding assays |  |  |  |  |  |  |  |  |  |  |
|  |  |  |  |  |  |  | Omega-6 intake: per 1 SD | |  |  |  |  |  |  |  |  |  |  |  |  |  |  |  |
| Niinistö 2015^51^ | Finland | Finnish  Type 1 Diabetes Prediction and Prevention (DIPP) | Cohort | 2939 | 81 | 6.1 years (1.3-13.2) | Omega-3 intake: per 1 SD | | semi-quantitative FFQ | Lactation (3d month) | | T1D | Clinical diagnosis | Some | Yes | Some | | | | genetic risk group (0 = moderate), familial diabetes (0 = no), maternal vocational education (1 = none) and duration of exclusive breastfeeding | Serious | | |
|  |  |  |  |  |  |  | Omega-6 intake: per 1 SD | |  |  |  |  |  |  |  |  |  |  |  |  |  |  |  |
|  |  |  |  |  | 172 | 4.4 years (0.5-13) | Omega-3 intake: per 1 SD | |  |  |  | IA | Immunofluorescence, microassay, radiobibding assays |  |  |  |  |  |  |  |  |  |  |
|  |  |  |  |  |  |  | Omega-6 intake: per 1 SD | |  |  |  |  |  |  |  |  |  |  |  |  |  |  |  |
| Niinistö 2017^52^ | Finland | Finnish  Type 1 Diabetes Prediction and Prevention (DIPP) | Nested case-control | 688 | 222 | 3.0 years (0.5-9.2) | Omega-3 proportion in serum: continuous | | gas chromatography | Infancy (6 months) | | IA | Immunofluorescence, microassay, radiobibding assays | No | Yes | Some | | | | sex, HLA-conferred DQB1 risk group (moderate or high risk), delivery hospital, the date of birth within 3 months in relation to the case children and not being from the same family as the case children, cow's milk consumption, familial diabetes and maternal vocational education | Moderate | | |
|  |  |  |  |  |  |  | Omega-6 proportion in serum: continuous | |  |  |  |  |  |  |  |  |  |  |  |  |  |  |  |
| Norris  2003^53^ | USA | The Diabetes Autoimmunity Study in the Young (DAISY) | Cohort | 1170 | 21 | 3.9 ± 2.5 years | Age at cow's milk introduction: 4-6 months vs 1-3 months | | Telephone or face to face interview | Infancy (4 months) | | IA | Micro-insulin autoantibody assay with 58% sensitivity and 99% specificity | No | Some | Some | | | | breastfed when exposed to cow's milk, HLA genotype, family history of T1D | Moderate | | |
|  |  |  |  | 1183 | 34 | 3.9 ± 2.2 years | Age at cereal introduction: ≥ 7 months vs 4-6 months 4-6 months vs 1-3 months | |  |  |  |  |  |  |  |  |  |  |  | breastfed when exposed to cereal, HLA genotype, ethnicity, family history of T1D, maternal age |  |  |  |
| Norris  2007^54^ | USA | The Diabetes Autoimmunity Study in the Young (DAISY) | Cohort | 1770 | 58 | 4.8 ± 2.6 years | Omega-3 intake: per 1 SD (0.778) | | semi-quantitative FFQ | Annually (exposure before year of diagnosis) | | IA | Radiobinding assays | No | Some | Some | | | | Total caloric intake, HLA-DR3/4,DQB1*0302 status, and family history of type 1 diabetes, omega-3 and omega-6 mutually adjusted | Moderate | | |
|  |  |  |  |  |  |  | Omega-6 intake: per 1 SD (6.252) | |  |  |  |  |  |  |  |  |  |  |  |  |  |  |  |
|  |  |  |  |  | 45 |  | Omega-3 intake: per 1 SD (0.778) | |  |  |  | Multiple autoanti-bodies or T1D | Radiobinding assays/ Clinical diagnosis |  |  |  |  |  |  |  |  |  |  |
|  |  |  |  |  |  |  | Omega-6 intake: per 1 SD (6.252) | |  |  |  |  |  |  |  |  |  |  |  |  |  |  |  |
| Norris  2014^55^ | USA | The Diabetes Autoimmunity Study in the Young (DAISY) | Case-cohort | 357 | 58 | 5.4 ± 3.3 years | Omega-3 percentage in erythrocyte membrane:  per 1% | | gas chromatography-mass spectrometry | Annually (exposure before year of diagnosis) | | IA | Radioimmunoassays | No | Some | Some | | | | HLA-DR status and family history of type 1 diabetes | Moderate | | |
|  |  |  |  |  |  |  | Omega-6 percentage in erythrocyte membrane:  per 1 SD (3.62) | |  |  |  |  |  |  |  |  |  |  |  |  |  |  |  |
| Norris  2018^56^ | USA & Europe | The Environmental Determinants  of Diabetes in the Young (TEDDY) | Nested case-control | 1341 | 360 | 21 months (2-4.5) | Plasma 25(OH)D levels: per 10 nmol/l | | ARCHITECT 25(OH)D chemiluminescent microparticle immunoassay | Infancy (before 12 months) | | IA | Radiobinding assays | No | Yes | Some | | | | clinical center, sex, and family history of type 1 diabetes, HLA-DR3/4 status and the first two PCs indicating ancestry, age and season of sample collection at the first visit | Moderate | | |
| Patterson 1994^57^ | N. Ireland |  | Case-control | 1548 | 258 | < 15 years | Breastfeeding: any vs none | | Specific question | Infancy (2 weeks) | | T1D | Hospital records | No | No | No | | | | sex, age, health board, social class | Serious | | |
|  |  |  |  |  |  |  | Exclusive breastfeeding: any vs none | |  |  |  |  |  |  |  |  |  |  |  |  |  |  |  |
| Pundziūtė-Luckå 2004^58^ | Sweden |  | Case-control | 279 | 99 | 7-14 years | Milk intake:  > 75th percentile vs ≤ 75th percentile | | FFQ | Year before diagnosis/interview | | T1D | Hospital records | No | No | No | | | | age, sex, and geographical region within Stockholm, energy intake | Serious | | |
|  |  |  |  |  |  |  | Carbohydrate intake: > 75th percentile vs ≤ 75th percentile | |  |  |  |  |  |  |  |  |  |  |  |  |  |  |  |
|  |  |  |  |  |  |  | Sugar intake: >75th percentile vs ≤ 75th percentile | |  |  |  |  |  |  |  |  |  |  |  |  |  |  |  |
|  |  |  |  |  |  |  | Sugar sweetened beverages intake: > 75th percentile vs ≤ 75th percentile | |  |  |  |  |  |  |  |  |  |  |  |  |  |  |  |
|  |  |  |  |  |  |  | Protein intake: > 75th percentile vs ≤ 75th percentile | |  |  |  |  |  |  |  |  |  |  |  |  |  |  |  |
| Raab  2014^59^ | Germany | BABYDIABBABYDIET TEENDIAB | Cohort | 161 | 53 | < 14 years | Plasma Vitamin D levels: per 10 nmol/l | | Radioimmunoassay | Child | | T1D | Clinical diagnosis: American Diabetes Association criteria | Yes | No | Yes | | | | age, season, study type | Serious | | |
| Radon  2005^60^ | Germany |  | Case-control | 466 | 242 | 6.7 ± 3.4 | Exclusive breastfeeding: yes vs no | | questionnaire | Infancy | | T1D | Hospital records | No | No | Some | | | | age, sex, center, family history of diabetes, kindergarden attendance, BMI, rhinitis, raw milk consumption during the first year of life, regular contact to stables during 1st year of life and 2nd to 6th year of life | Serious | | |
| Robertson 2010^61^ | Scotland |  | Case-control | 1444 | 361 | < 15 years | Breastfeeding at discharge from hospital: yes vs no | | Health records | Infancy | | T1D | Scottish Study Group for the Care of Diabetes in the Young Register  & Aberdeen Maternity Neonatal Databank | No | No | No | | | | year of birth, maternal deprivation | Serious | | |
| Rosenbauer  2008^62^ | Germany |  | Case-control | 2454 | 719 | < 5 years | Breastfeeding duration: ≥ 5 months vs < 2 weeks | | Questionnaire | Infancy | | T1D | Hospital-based  surveillance system ESPED | No | No | Some | | | | age (±12 month), sex, and place of residence current cow’s milk consumption, familial type 1 diabetes, social status, maternal age, change of residence, number of children, and birth weight | Serious | | |
|  |  |  |  | 2443 | 718 |  | Age at infant formula introduction: ≥ 5 months vs < 2 weeks | |  |  |  |  |  |  |  |  |  |  |  |  |  |  |  |
|  |  |  |  | 2631 | 760 |  | Age at cow's milk introduction:  ≥ 1 year or no exposure vs < 1 year | |  |  |  |  |  |  |  |  |  |  |  | matched on age (±12 month), sex, and place of residence |  |  |  |
|  |  |  |  |  |  |  | Age at solid food introduction:  ≥ 5 months vs ≤ 4 months | |  |  |  |  |  |  |  |  |  |  |  |  |  |  |  |
|  |  |  |  |  |  |  | Coffee intake: 1-2 cups/day vs none | |  | Fetal | |  |  |  |  |  |  |  |  |  |  |  |  |
| Sadauskaite-Kuehne 2004^63^ | Sweden | Diabetes and Environment around the Baltic Sea (DEBS) | Case-control | 517 | 166 | 5-9 years | Breastfeeding duration: ≥ 7 months vs < 7 months | | Questionnaire | Infancy | | T1D | Diabetes registers | No | No | No | | | | age, sex, mother ≥ 35 years, prematurity, Treatment at hospital during 1 month of life, Infection during 1 month of life, Neonatal jaundice during 1st week, Mother’s toxicosis during pregnancy, Infection during last 6 months, Stressful event during last 6 months, Family living in a city | Serious | | |
|  | Lithuania |  |  | 302 | 81 |  | Exclusive breastfeeding duration: ≥ 2 months vs < 2 months | |  |  |  |  |  |  |  |  |  |  |  | mother ≥ 35 years, prematurity, Treatment at hospital during 1 age, sex, month of life, Infection during 1 month of life, Neonatal jaundice during 1st week, Mother’s toxicosis during pregnancy, Infection during last 6 months, Family living in a city, Mother’s education (higher than secondary) |  |  |  |
|  | Sweden |  |  | 538 | 167 |  | Age at cow's milk introduction:  ≥ 7 months vs < 7 months | |  |  |  |  |  |  |  |  |  |  |  | age, sex, prematurity, Treatment at hospital during 1 month of life, Infection during 1 month of life, Neonatal jaundice during 1st week, Mother’s toxicosis during pregnancy, Infection during last 6 months, Stressful event during last 6 months, Family living in a city |  |  |  |
|  |  |  |  | 266 | 92 |  | Age at infant formula introduction: ≥ 3 months vs < 3 months | |  |  |  |  |  |  |  |  |  |  |  | age, sex, mother ≥ 35 years, prematurity, Treatment at hospital during 1 month of life, Infection during 1 month of life, Neonatal jaundice during 1st week, Mother’s toxicosis during pregnancy, Infection during last 6 months, Stressful event during last 6 months, Family living in a city |  |  |  |
| Saukkonen 1998^64^ | Finland | Finnish Childhood Diabetes in  Finland (DiMe) | Case-control | 820 (410 sibling pairs) | 410 | < 15 years | Breastfeeding duration: ≥ 4 months vs < 4 months | | Questionnaire | Infancy | | T1D | Diabetes registry | No | Some | No | | | | supplementary milk feeding, overall breastfeeding/current milk consumption, cow's milk formula IgA, cow's milk formula IgG, cow's milk formula IgM, BLG IgA, BLG IgG, BSA IgA, BSA IgG, DQB1*0201/0302 or *0302/xd | Serious | | |
|  |  |  |  |  |  |  | Cow's milk intake: ≥ 3 glasses (180 ml)/day vs < 3 glasses/day | |  | Before diagnosis | |  |  |  |  |  |  |  |  |  |  |  |  |
| Silvis  2019^65^ | USA & Europe | The Environmental Determinants  of Diabetes in the Young (TEDDY) | Cohort | 8260 | 747 | 35 months (18-70) | Vitamin D supplementation:  yes vs no | | Specific question | Fetal | | IA | Radiobinding assays | No | Yes | Some | | | | HLA-DR-DQ genotype, family history of T1D, sex, and country | Moderate | | |
|  |  |  |  |  |  |  | Omega-3 supplementation:  per 100g increase (cumulative intake during whole pregnancy, 0,4g/day) | |  |  |  |  |  |  |  |  |  |  |  |  |  |  |  |
| Simpson 2011^66^ | USA | Diabetes Autoimmunity Study in the Young (DAISY) | Cohort | 185 | 55 | 8.6 ± 3.9 years | Plasma 25(OH)D levels: per 10 nmol/l | | Radioimmunoassay | Child | | T1D | Clinical diagnosis | Yes | Some | Some | | | | family history of T1D, HLA-DR3/4, DQB1*0302 genotype, ethnicity, and age at first appearance of autoantibodies | Moderate | | |
|  |  |  | Case-cohort | 128 | 30 | 3.5 ± 2.6 years | Plasma 25(OH)D levels: per 10 nmol/l | |  | Infancy  (9 months) | | IA | Radioimmunoassays |  |  |  |  |  |  | family history of T1D, and HLA-DR3/4, DQB1*0302 genotype |  |  |  |
| Šipetić  2004^67^ | Serbia | The Belgrade childhood diabetes study | Hospital-based case-control | 315 | 105 | ≤ 16 years | Coffee intake:  any vs none | | Questionnaire | Fetal | | T1D | Clinical diagnosis: WHO criteria | No | No | Some | | | | age, sex and place of residence, socioeconomic status-poor, higher education of father, alcohol consumption by father, family members > 3, complications during pregnancy, ultrasound scan during pregnancy, gestational age > 41 weeks, nitrosamine-rich food during pregnancy | Serious | | |
| Šipetić 2005a^68^ | Serbia | The Belgrade childhood diabetes study | Hospital-based case-control | 315 | 105 | ≤ 16 years | Nitrosoamines-rich foods intake: high amount vs low amount | | Questionnaire | Fetal | | T1D | Clinical diagnosis: WHO criteria | No | No | Some | | | | age, sex and place of residence, stressful events, and symptoms of psychological dysfunction during the 12 months preceding the onset of the disease, irregular vaccination, infection during the 6 months preceding the onset of disease, use of ultrasound diagnostic technologies, higher education of father, alcohol consumption by father, and type 1 and type 2 diabetes in three generations of children’s relatives | Serious | | |
| Šipetić 2005b^69^ | Serbia | The Belgrade childhood diabetes study | Hospital-based case-control | 315 | 105 | ≤ 16 years | Breastfeeding duration: ≥ 4 months vs < 4 months | | Questionnaire | Infancy | | T1D | Clinical diagnosis: WHO criteria | No | No | Some | | | | age, sex and place of residence, father’s education, number of family members, socioeconomic status, gestational age, and postnatal intensive care | Serious | | |
|  |  |  |  |  |  |  | Age at cow's milk introduction:  ≥ 5 months vs < 5 months | |  |  |  |  |  |  |  |  |  |  |  |  |  |  |  |
| Skaaby  2015^70^ | Denmark | Monica10, Inter99, Health2006 | Cohort | 11521 | 135 | > 18 years | Serum 25(OH)D levels:  per 10 nmol/L | | No information | Adult | | T1D | Danish National  Patient Register | No | No | No | | | | gender, education, season of blood sample, physical activity, smoking habits, alcohol intake, body mass index, systolic and diastolic blood pressure, serum total cholesterol, and serum triglycerides | Serious | | |
| Skrodeniené 2010^71^ | Lithuania | Diabetes and Environment around the Baltic Sea  (DEBS) | Case-control | 202 | 124 | 9.2 ± 3.9 years | Breastfeeding duration: ≥ 3 months vs < 3 months | | Questionnaire | Infancy | | T1D | Diabetes registry | No | Some | No | | | | age, sex, mother's residence during pregnancy, at least one of the protective HLA haplotypes, at least one of the risk HLA haplotypes, rubella infection, infection during the last 6 months before diagnosis | Serious | | |
| Stene  2003^72^ | Norway |  | Case-control | 2011 | 477 | 10.9 ± 3.4 years | Vitamin D supplementation: ≥ 5 times/week vs no | | Questionnaire | Fetal | | T1D | National Childhood Diabetes Registry | No | No | Some | | | | maternal use of cod liver oil during pregnancy, child’s use of cod liver oil or other vitamin D supplements during the first year of life, duration of exclusive breastfeeding, child’s age at introduction of solid foods, maternal education, maternal smoking during pregnancy, maternal age at delivery, child’s number of siblings, type 1 diabetes among siblings or parents, and the child’s age, sex | Serious | | |
|  |  |  |  | 2051 | 482 |  |  |  |  | Infancy | |  |  |  |  |  |  |  |  |  |  |  |  |
|  |  |  |  | 2074 | 499 |  | Cod liver oil supplementation: per 1 time/week | |  | Fetal | |  |  |  |  |  |  |  |  |  |  |  |  |
|  |  |  |  | 2126 | 515 |  |  |  |  | Infancy | |  |  |  |  |  |  |  |  |  |  |  |  |
|  |  |  |  | 2213 | 545 |  | Exclusive breastfeeding duration: 3.5-6 months vs no | |  |  |  |  |  |  |  |  |  |  |  | age, sex, maternal education, maternal smoking in pregnancy, maternal age at delivery, introduction to solid foods, number of siblings, sibling or parent with T1D |  |  |  |
|  |  |  |  | 2118 | 517 |  | Age at solid food introduction (mainly porridge from wheat): ≥ 5 months vs ≤ 3 months | |  |  | |  |  |  |  |  |  |  |  | age, sex, maternal education, maternal smoking in pregnancy, maternal age at delivery, duration of exclusive breastfeeding, number of siblings, sibling or parent with T1D |  |  |  |
| Størdal  2018^73^ | Norway | Mother and Child Cohort Study  (MoBa) | Cohort | 94209 | 373 | 7.5 years (0.7-15.1) | Iron supplementation: yes vs no | | Questionnaire | Fetal | | T1D | Norwegian Childhood Diabetes  Registry  &  Norwegian Patient  Registry | No | No | No | | | | maternal age and education, smoking, parity, pre-pregnancy BMI, mode of delivery, birth weight and prematurity, maternal type 1 diabetes, maternal celiac disease and diagnosed maternal anaemia (<17 weeks) | Moderate | | |
| Svensson 2005^74^ | Denmark |  | Case-control | 1152 | 475 | 8.4 years | Vitamin D supplementation: yes vs no | | questionnaire | Infancy | | T1D | Diabetes registry | No | No | Some | | | | maternal age, birth cohort and gender | Serous | | |
|  |  |  |  |  | 466 |  | Age at cow's milk introduction:  ≥ 3 months vs < 3 months | |  |  |  |  |  |  |  |  |  |  |  |  |  |  |  |
| Syrjälä  2019^75^ | Finland | Finnish  Type 1 Diabetes Prediction and Prevention (DIPP) | Cohort | 5545 | 195 | 6.4 years (4.2-10.0) | Egg intake:  per 50 g/day | | 3-day food records | Annually until the age of 6 | | T1D | National pediatric diabetes registry | Some | Yes | Some | | | | sex of the child, genetic risk of the child and familial diabetes | Moderate | | |
|  |  |  |  |  |  |  | Meat and meat products intake: per 60 g/day | |  |  |  |  |  |  |  |  |  |  |  |  |  |  |  |
|  |  |  |  |  |  |  | Fish and fish products intake: per 60 g/day | |  |  |  |  |  |  |  |  |  |  |  |  |  |  |  |
| Sørensen 2016^76^ | Norway |  | Nested case-control | 235 | 80 | 9.0 ± 3.5 years | Serum 25(OH)D levels: per 10 nmol/l | | Radioimmunoassay | Fetal | | T1D | Norwegian Childhood Diabetes Registry | No | No | Some | | | | sex of the child, maternal diabetes, and season of blood sample (January–March, April–June, July–September, October–December) and levels of vitamin D-binding protein | Moderate | | |
| Tai  1998^77^ | Taiwan |  | Case-control | 307 | 116 | 8.3 ± 3.3 years | Breastfeeding: yes vs no (cow's milk) | | Questionnaire/ interview | Infancy | | T1D | Diabetes registry | No | No | No | | | | age, sex, and parental and individual educational levels | Serous | | |
| Tenconi 2007^78^ | Italy |  | Hospital-based case-control | 477 | 159 | 15.5 ± 8.3 years | Vitamin D supplementation: yes vs no | | telephone questionnaire | Lactation | | T1D | Diabetes registry | No | No | Some | | | | age, sex | Serious | | |
|  |  |  |  |  |  |  | Exclusive breastfeeding duration: ≥ 3 months vs < 3 months | | specific question (at diagnosis) | Infancy | |  |  |  |  |  |  |  |  |  |  |  |  |
| Thorsen 2018^79^ | Norway & Denmark | Danish National Birth Cohort  (DNBC)  &  Mother and Child Cohort Study  (MoBa) | Case-cohort | 2020 | 459 | 5.7-9.0 years | Plasma 25(OH)D levels: per 10 nmol/L | | liquid chromatography–tandem mass spectrometry | At birth | | T1D | Danish Childhood Diabetes  Registry  & Norwegian Diabetes Childhood Registry | No | No | Some | | | | maternal diabetes, age at time of delivery, prepregnancy body mass index, child’s sex, birth weight, and the time of year/season that each blood sample was taken | Moderate | | |
|  |  |  |  |  |  |  |  |  |  | Fetal | |  |  |  |  |  |  |  |  |  |  |  |  |
| Thorsen 2019^80^ | Denmark | Danish National Birth Cohort  (DNBC) | Case-cohort | 63931 | 238 | 9.8 years (0.9-16.9) | Pure iron supplements: yes vs no | | telephone interview | Fetal | | T1D | Danish Childhood Diabetes  Registry | No | No | Some | | | | parental socio-economic status, mode of delivery, pre-pregnancy BMI, age, smoking status, parity, gestational age, maternal age, breastfeeding, maternal celiac disease, maternal type I diabetes and maternal anemia | Moderate | | |
|  |  |  |  | 51859 | 191 |  | Iron droplets use: yes vs no | |  | Infancy (< 18 months) | |  |  |  |  |  |  |  |  |  |  |  |  |
| Uusitalo U 2018^81^ | USA & Europe | The Environmental Determinants  of Diabetes in the Young (TEDDY) | Cohort | 7311 | 694 | 33 months (16-62) | Breastfeeding duration:  per additional month | | Food diary | Infancy (up to 24 months) | | IA | Radiobinding assays | No | Yes | Some | | | country, HLA genotype, FDR status, sex of the child, and probiotic use < 28 days | | Moderate | | |
|  |  |  |  | 7563 | 703 |  | Exclusive breastfeeding duration: per additional month | |  |  |  |  |  |  |  |  |  |  |  |  |  |  |  |
|  |  |  |  | 7374 | 696 |  | Age at gluten containing cereal introduction:  4-9 months vs < 4 months | |  |  |  |  |  |  |  |  |  |  |  |  |  |  |  |
|  |  |  |  | 6604 | 598 |  | Age at infant formula introduction: per month of introduction delay | |  |  |  |  |  |  |  |  |  |  |  |  |  |  |  |
|  |  |  |  | 7540 | 701 |  | Age at solid food introduction: per month of introduction delay | |  |  |  |  |  |  |  |  |  |  |  |  |  |  |  |
|  |  |  |  | 6810 | 699 |  | Age at cereal introduction:  > 4 months vs ≤ 4 months | |  |  |  |  |  |  |  |  |  |  |  |  |  |  |  |
|  |  |  |  | 6806 | 700 |  | Age at fruit introduction (including berries): > 4 months vs ≤ 4 months | |  |  |  |  |  |  |  |  |  |  |  |  |  |  |  |
|  |  |  |  | 7507 | 701 |  | Age at root vegetable introduction: per 1 month delay | |  |  |  |  |  |  |  |  |  |  |  |  |  |  |  |
|  |  |  |  | 6872 | 662 |  | Age at fish or sea-food introduction: per 1 month delay | |  |  |  |  |  |  |  |  |  |  |  |  |  |  |  |
|  |  |  |  | 6676 | 692 |  | Age at meat introduction: > 8 months vs ≤ 8 months | |  |  |  |  |  |  |  |  |  |  |  |  |  |  |  |
| Wadsworth 1997^82^ | UK |  | Case-control | 430 | 215 | < 5 years | Age at infant formula introduction:  ≥ 4 months vs early introduction < 2 weeks | | Specific question | Infancy | | T1D | British Paediatric  Association Surveillance Unit reporting framework | No | No | Some | | | | age, sex | Serious | | |
| Verge  1994^83^ | Australia |  | Case-control | 475 | 217 | < 15 years | Breastfeeding duration: 2-7 months vs < 2 months | | Questionnaire | Infancy | | T1D | Diabetes registry | No | No | No | | | | age, sex, maternal education level | Serious | | |
|  |  |  |  |  |  |  | Exclusive breastfeeding duration: ≥ 3 months vs < 3 months | |  |  |  |  |  |  |  |  |  |  |  |  |  |  |  |
|  |  |  |  |  |  |  | Age at cow's milk formula introduction: > 3 months or never vs ≤ 3 months | |  |  |  |  |  |  |  |  |  |  |  |  |  |  |  |
|  |  |  |  |  |  |  | Cow's milk protein intake: per 10 g/day | | semi-quantitative FFQ | Year before diagnosis | |  |  |  |  |  |  |  |  | age, sex, fluid intake and cereal protein intake |  |  |  |
|  |  |  |  |  |  |  | Intake of foods rich in nitrosamine: highest tertile vs lowest tertile | |  |  |  |  |  |  |  |  |  |  |  | age, sex, fluid intake, cow's milk, and cereal protein intake |  |  |  |
| Viner 2008^84^ | UK | The 1970 British Cohort Study (BCS70) | Cohort | 9062 | 47 | 20 years (10-30) | Breastfeeding duration: 1-3 months vs none | | Questionnaire | Infancy (assessed at age of 5 years) | | T1D | Self-reported | No | No | No | | | | birthweight, height z-score (5-years), BMI z-score (10 years), Height z-score (10 years), signs of puberty at 10 years, social class, maternal education, sex | Moderate | | |
| Virtanen  1991^85^ | Finland | Childhood Diabetes in Finland (DiMe) | Case-control | 178 | 89 | < 7 years | Breastfeeding duration: ≥ 3 months vs < 3 months | | Questionnaire | Infancy | | T1D | Diabetes registry | No | No | No | | | | age, sex, maternal education | Serious | | |
|  |  |  |  | 144 | 72 |  | Exclusive breastfeeding duration: ≥ 3 months vs < 3 months | |  |  |  |  |  |  |  |  |  |  |  |  |  |  |  |
|  |  |  |  | 174 | 87 |  | Age at infant formula introduction:  ≥ 3 months vs < 3 months | |  |  |  |  |  |  |  |  |  |  |  |  |  |  |  |
| Virtanen  1992^86^ | Finland | Childhood Diabetes in Finland (DiMe) | Case-control | 852 | 426 | 7-14 years | Breastfeeding duration: ≥ 3 months vs < 3 months | | Questionnaire | Infancy | | T1D | Diabetes registry | No | No | No | | | | age, sex | Serious | | |
|  |  |  |  |  |  |  | Exclusive breastfeeding duration: ≥ 3 months vs < 3 months | |  |  |  |  |  |  |  |  |  |  |  |  |  |  |  |
| Virtanen 1994b^87^ | Finland | Childhood Diabetes in Finland (DiMe) | Case-control | 1136 | 600 | < 14 years | Coffee intake: 1 cup (110 ml)/day vs < 1 cup/day | | Questionnaire | Before diagnosis | | T1D | Diabetes registry | No | No | No | | | | mother's education (< 14 years, ≥ 14 years), child's age, sex | Serious | | |
| Virtanen 1994c^88^ | Finland | Childhood Diabetes in Finland (DiMe) | Case-control | 1279 | 595 | < 14 years | Nitrate intake: 4th quartile vs 1st quartile | | FFQ | Half year before introduction to the study | | T1D | Diabetes registry | No | No | No | | | | age, sex, mother's education (< 13 years, ≥ 13 years), child's age, place of residence | Moderate | | |
|  |  |  |  |  |  |  | Nitrite intake: 4th quartile vs 1st quartile | |  |  |  |  |  |  |  |  |  |  |  |  |  |  |  |
| Virtanen  1998^89^ | Finland | Childhood Diabetes in Finland (DiMe) | Cohort | 725 | 33 | 3-26 years | Breastfeeding duration: ≥ 2 months vs < 2 months | | Questionnaire | Infancy | | T1D | National Central Drug Registry | Some | No | Yes (sibling) | | | | age, sex, maternal age, age at introduction of supplementary milk feeding, childhood milk and sour milk consumption, length of maternal education | Moderate | | |
|  |  |  |  | 602 | 43 | 3-23 years | Breastfeeding duration: ≥ 2 months vs < 2 months | |  |  |  | IA | Lab assessment |  |  |  |  |  |  |  |  |  |  |
|  |  |  |  |  | 38 |  | Age at cow's milk products introduction (infant formulas, cow’s milk, other cow’s milk products):  ≥ 2 months vs < 2 months | |  |  |  |  |  |  |  |  |  |  |  | age, sex, maternal age, duration of total breast feeding, childhood milk and sour milk consumption, length of maternal education |  |  |  |
|  |  |  |  |  | 45 |  | Milk intake:  ≥ 3 glasses (180 g)/day vs < 3 glasses/day | |  | Half year before introduction to the study | |  |  |  |  |  |  |  |  | age, sex, maternal age, age at introduction of supplementary milk feeding, duration of total breast feeding, length of maternal education |  |  |  |
| Virtanen  2000^90^ | Finland | Childhood Diabetes in Finland (DiMe) | Nested case-control | 255 | 29 | 7.2 years | Age at cow's milk products introduction (infant formulas, cow’s milk, other cow’s milk products):  ≥ 2 months vs < 2 months | | Questionnaire | Infancy | | T1D | National Central Drug Registry | No | Some | Yes (sibling) | | | | matching factors (age, sex, start of follow-up), maternal education, maternal and child’s ages, HLA genotype, age at introduction of cow's milk/cow’s milk intake | Moderate | | |
|  |  |  |  | 287 | 33 |  | Milk intake: ≥ 3 glasses (180 g)/day vs < 3 glasses/day | |  | Half year before introduction to the study (3-19 years) | |  |  |  |  |  |  |  |  |  |  |  |  |
| Virtanen  2006^91^ | Finland | Finnish  Type 1 Diabetes Prediction and Prevention (DIPP) | Cohort | 3184 | 97 | < 4 years | Breastfeeding duration: > 8.5 months vs < 4 months | | Questionnaire | Infancy | | IA | Immunofluorescence, microassay, radiobibding assays | No | Yes | Some | | | | genetic risk | Serious | | |
|  |  |  |  | 3430 | 104 |  | Exclusive breastfeeding duration: ≥ 3 months vs 1-2.99 months | |  |  |  |  |  |  |  |  |  |  |  |  |  |  |  |
|  |  |  |  | 3417 | 103 |  | Age at cow's milk introduction:  ≥ 4 months vs 1-3.99 months | |  |  |  |  |  |  |  |  |  |  |  |  |  |  |  |
|  |  |  |  | 3234 | 101 |  | Age at cereal introduction (wheat, rye, oats or barley): ≥ 5.5 months vs 5-5.5 months ≥ 5.5 months vs < 5 months | | Diary of new foods |  |  |  |  |  |  |  |  |  |  |  |  |  |  |
|  |  |  |  | 3286 | 101 |  | Age at fruit introduction (including berries):  > 4 months vs < 3.5 months | |  |  |  |  |  |  |  |  |  |  |  | sex, genetic risk, familial diabetes, gestational age, maternal age, educational level and smoking during pregnancy, number of siblings at the time of birth, and for area of birth (Oulu vs Tampere) |  | | |
| Virtanen  2011a^92^ | Finland | Finnish  Type 1 Diabetes Prediction and Prevention (DIPP) | Cohort | 5630 | 237 | < 10 years | Age at root vegetable introduction: per 1 month delay | | Diary of new foods | Infancy | | IA | Immunofluorescence, microassay, radiobibding assays | No | Yes | Some | | | | sex, HLA risk, familial diabetes, hospital of birth, number of siblings, maternal education, maternal age, gestational age | Moderate | | |
| Virtanen 2011b^93^ | Finland | Finnish  Type 1 Diabetes Prediction and Prevention (DIPP) | Cohort | 3723 | 138 | < 5 years | Fruit intake: per 100 g/day | | FFQ | Fetal | | IA | Immunofluorescence, microassay, radiobibding assays | No | Yes | Some | | | | age, genetic risk, familial diabetes, energy intake | Serious | | |
|  |  |  |  |  |  |  | Vegetable intake: per 100 g/day | |  |  |  |  |  |  |  |  |  |  |  |  |  |  |  |
|  |  |  |  |  |  |  | Root vegetable intake:  per 100 g/day | |  |  |  |  |  |  |  |  |  |  |  |  |  |  |  |
|  |  |  |  |  |  |  | Egg intake: per 50 g/day | |  |  |  |  |  |  |  |  |  |  |  |  |  |  |  |
|  |  |  |  |  |  |  | Coffee intake: highest quarter vs lowest quarter | |  |  |  |  |  |  |  |  |  |  |  |  |  |  |  |
| Visalli  2003^94^ | Italy | EURODIAB ACE | Case-control | 900 | 150 | < 18 years | Breastfeeding duration: ≥ 3 months vs < 3 months | | Questionnaire | Infancy | | T1D | Diabetes registry | No | No | Some | | | | age, family history of T1D, infectious diseases during mother's pregnancy, occurence of eczema | Serious | | |
| Ziegler  2003^95^ | Germany | BABYDIAB | Cohort | 1460 | 81 | < 8 years | | Breastfeeding duration: 3.1-6 months vs < 3 months | Questionnaire | | Infancy | IA | Radiobinding assays | No | Some | | Yes (parent with T1D) | maternal type 1 diabetes mellitus, gestation age before 36 weeks, birth weight below 2700 g, and region of residence | | | |  |  |
|  |  |  |  |  |  |  |  | Exclusive breastfeeding duration: 3.1-6 months vs < 3 months |  |  |  |  |  |  |  |  |  |  |  |  |  | Moderate |  |
|  |  |  |  | 1282 | 73 |  |  | Milk-based food supplements only < 3 months of age: yes vs no food supplementation | Questionnaire, food record | |  |  |  |  |  |  |  |  |  |  |  |  |  |

* In categorical exposures, when the reference group was representing the highest category, it was shifted to the lowest. Quantities of continuous exposures were transformed to the most frequent unit. ^†^ This column refers to the presence of islet autoimmunity at baseline where ‘Yes’ indicates islet autoimmunity at baseline, ‘No’ indicates no islet autoimmunity or no assessment of islet autoimmunity at baseline, and ‘Some’ indicates that some individuals had islet autoimmunity at baseline. T1D, type 1 diabetes; IA, islet autoimmunity; HLA, human leukocyte antigen; SD, standard deviation; FFQ, food frequency questionnaire

**Supplementary Table 7. Characteristics of eligible randomized controlled trials**

| **Study** | **Country** | **RCT name** | **N total** | **N  cases** | **Intervention** | **Control** | **Duration** | **Time of exposure** | **Outcome** | **Outcome assessment** | **IA**^*^ | **HLA risk** | **Family history** | **Follow up** | **Hazard ratio (95% CI)** | **Adjustment** | **Risk of bias** |
| --- | --- | --- | --- | --- | --- | --- | --- | --- | --- | --- | --- | --- | --- | --- | --- | --- | --- |
| Åkerblom 2005^96^ | Finland | Trial to Reduce IDDM in the Genetically at Risk  (TRIGR) | 208 | 32 | Weaning to extensively hydrolyzed casein-based formula | Weaning to cow's milk-based formula (80% intact milk protein and 20% hydrolyzed casein-based formula) | At least 60 days | First 6-8 months of life | IA | Radiobinding assays and immunofluorescence | No | Yes | Yes | 4.7 years | 0.44  (0.20 to 0.93) | Duration of study formula feeding | Low |
| Knip  2010^97^ |  |  | 208 | 50 |  |  |  |  |  |  |  |  |  | 4.8 years | 0.51  (0.28 to 0.91) |  | Low |
|  |  |  | 230 | 16 |  |  |  |  | T1D | National drug reimbursement register, clinical diagnosis | Some | Yes | Yes | 4.8 years | 0.48  (0.14 to 1.61) |  |  |
| Knip  2014^98^ | 15 countries (Australia, Canada, Europe, USA) |  | 2070 | 845 |  |  |  |  | IA | Radiobinding assays and immunofluorescence | No | Yes | Yes | 6.3 years | 1.09  (0.95 to 1.24) | HLA risk, duration of breastfeeding, vitamin D use, study formula duration and consumption, and region | Low |
| Knip 2018^99^ |  |  | 2159 | 173 |  |  |  |  | T1D | Clinical diagnosis based on the WHO criteria | Some | Yes | Yes | 11 years | 1.1  (0.8 to 1.5) | HLA risk, duration of breastfeeding and formula consumption, sex, and region | Low |
| Lampeter 1998^100^ | Austria & Germany | The Deutsche Nicotinamide Intervention Study | 55 | 11 | Nicotinamide (slow release, 1.2 g/m^2^ body surface per day) | Placebo | 3 years | 3-12 years | T1D | Clinical diagnosis | Yes ICAs | No | Yes siblings | 3 years | 0.79  (0.25 to 3.38) |  | Some concerns |
| Hummel 2011^101^ | Germany | BABYDIET | 150 | 16 | Introduction to gluten at 12 months | Introduction to gluten at 6 months |  |  | IA | Radiobinding assays | No | Some | Yes | 3 years | 1.3  (0.6 to 3.0) | Breastfeeding duration, breastfeeding during first gluten exposure, introduction of solid food, gluten dosage | Some concerns |

*This column refers to the presence of islet autoimmunity at baseline where ‘Yes’ indicates islet autoimmunity at baseline, ‘No’ indicates no islet autoimmunity or no assessment of islet autoimmunity at baseline, and ‘Some’ indicates that some individuals had islet autoimmunity at baseline. RCT, randomized controlled trial; IA, islet autoimmunity; T1D, type 1 diabetes; HLA, human leukocyte antigen; CI, confidence interval; ICAs, islet cell antibodies

**Supplementary Table 8. Characteristics of eligible observational studies that were excluded because other studies on the same cohort were selected**

| **Study** | **Country** | **Cohort name** | **Study design** | **N total** | **N  cases** | **Age at diagnosis** | **Exposure** | | | **Outcome** | | **Outcome assessment** | **IA**^*^ | **HLA risk** | **Family history** | **Relative risk (95% CI)** | **Adjusted factors** | **Risk of bias** |
| --- | --- | --- | --- | --- | --- | --- | --- | --- | --- | --- | --- | --- | --- | --- | --- | --- | --- | --- |
|  |  |  |  |  |  |  | **Assessment** | | **Time** |  |  |  |  |  |  |  |  |  |
| Blom  1989^102^ | Sweden | Same cohort as Dahlquist 1991^14^ | Case-control | 176 | 66 | < 7 years | Breastfeeding duration:  < 3 vs ≥ 3 months | Questionnaire | Infancy | | T1D | Clinical diagnosis | No | No | No | 1.7 (1.02 to 2.89) | age, sex, county | Serious |
| Kimpimäki 2001^103^ | Finland | Finnish  Type 1 Diabetes Prediction and Prevention  (DIPP) | Case-control | 455 | 65 | 2.5 years (0.8-3.8) | Exclusive breastfeeding duration:  ≥ 4 vs < 2 months | Questionnaire | Infancy | | IA | Immunofluorescence | No | Yes | No | 0.89 (0.43 to 1.83) | mother's age, duration of general education and relative height and weight at the age of 12 months | Serious |
|  |  |  |  |  |  |  | Age at cow's milk introduction:  < 2 vs ≥ 4 months |  |  |  |  |  |  |  |  | 1.02 (0.53 to 1.97) |  |  |
| Miller 2011^104^ | USA | Diabetes Autoimmunity Study in the Young (DAISY) | Cohort | 157 | 30 | 8.7 ± 3.9 years | Omega-3 intake:  per 1 SD increase | FFQ | Year before diagnosis | | T1D | Clinical diagnosis | Yes | Some | Some | 1.31 (0.65 to 2.62) | total caloric intake, type of FFQ, age at first autoantibody positive visit, HLA DR3/4 status, family history of type 1 diabetes, maternal age, maternal education, and maternal ethnicity | Moderate |
|  |  |  |  |  |  |  | Omega-6 intake:  per 1 SD increase |  |  |  |  |  |  |  |  | 1.50 (0.80 to 2.82) |  |  |
| Sørensen 2012^105^ | Norway | Same cohort as Sørensen 2016^76^ | Nested case-control | 328 | 109 | 9.0 ± 3.6 years | Serum 25(OH)D levels:  > 89 nmol/l vs ≤ 54 nmol/l | Radioimmunoassay | Fetal (3d trimester) | | T1D | Clinical diagnosis | No | No | No | 2.38 (1.12 to 5.07) | season of blood sample (January through March, April through June, July through September, and October through December) and sex of the child | Serious |
| Stene  2000^106^ | Norway |  | Case-control | 1058 | 78 | 11.1 ± 3.6 years | Vitamin D supplementation:  any vs none | Questionnaire | Infancy  (1st year) | | T1D | National Childhood Diabetes Registry | No | No | No | 1.27 (0.70 to 2.31) | age, sex, breastfeeding, maternal education, cod liver oil | Serious |
|  |  |  |  | 1131 | 84 |  | Cod liver oil supplementation:  any vs none |  | Fetal | |  |  |  |  |  | 0.36 (0.14 to 0.90) | age, sex, breastfeeding, maternal education, multivitamin |  |
|  |  |  |  | 1058 | 78 |  |  |  | Infancy (1st year) | |  |  |  |  |  | 0.82 (0.47 to 1.42) |  |  |
| Tapia  2019^107^ | Norway | Mother and Child Cohort Study  (MoBa) | Nested case-control | 705 | 174 | 5.7 years (0.7-12.7) | Plasma 25(OH)D levels:  per 1 nmol/L | Liquid chromatography–tandem mass spectrometry | Fetal (Mid-pregnancy) | | T1D | Clinical diagnosis | No | Some | No | 1.01 (1.00 to 1.01) | child`s HLA genotype, sex, maternal ethnicity, age, pre-pregnancy BMI, caesarean section, and smoking | Moderate |
|  |  |  |  | 717 | 175 |  |  |  | Infancy (Cord-blood) | |  |  |  |  |  | 0.99 (0.98 to 1.01) |  |  |
|  |  |  |  | 698 | 174 |  |  |  | Fetal (Postpartum) | |  |  |  |  |  | 1.00 (0.99 to 1.01) |  |  |
| Virtanen 2010^108^ | Finland | Finnish  Type 1 Diabetes Prediction and Prevention  (DIPP) | Nested case-control | 251 | 83 | 2.5 years | ALA percentage in serum: high vs low | Gas chromatography | ≥ 0.5 year before diagnosis | | IA | Immunofluorescence, microassay, radiobibding assays | No | Yes | Some | 0.86 (0.62 to 1.20) | gender, genetic risk group, site, and time of birth (range ± 3 months), vocational education of the mother and the father, maternal age, duration of gestation, type I diabetes in a first-degree relative, number of earlier deliveries and maternal smoking during pregnancy | Moderate |
|  |  |  |  |  |  |  | EPA percentage in serum: high vs low |  |  |  |  |  |  |  |  | 0.97 (0.73 to 1.30) |  |  |
|  |  |  |  |  |  |  | DHA percentage in serum: high vs low |  |  |  |  |  |  |  |  | 1.00 (0.71 to 1.41) |  |  |
|  |  |  |  |  |  |  | DPA percentage in serum: high vs low |  |  |  |  |  |  |  |  | 0.87 (0.63 to 1.21) |  |  |
|  |  |  |  |  |  |  | LA percentage in serum: high vs low |  |  |  |  |  |  |  |  | 0.67 (0.48 to 0.94) |  |  |
|  |  |  |  |  |  |  | ARA percentage in serum: high vs low |  |  |  |  |  |  |  |  | 1.06 (0.79 to 1.42) |  |  |
|  |  |  |  |  |  |  | GLA percentage in serum: high vs low |  |  |  |  |  |  |  |  | 1.15 (0.86 to 1.55) |  |  |
| Virtanen 1993^109^ | Finland | Childhood Diabetes in Finland  (DiMe) | Case-control | 1016 | 508 | ≤ 14 years | Breastfeeding duration:  ≥ 6 vs < 2 months or none | Questionnaire | Infancy | | T1D | Clinical diagnosis | No | No | No | 0.87 (0.47 to 1.63) | age, sex, age at introduction of dairy products | Serious |
|  |  |  |  |  |  |  | Age at cow's milk introduction:  ≥ 6 vs < 2 months |  |  |  |  |  |  |  |  | 0.62 (0.35 to 1.08) | age, sex, total breastfeeding duration |  |
|  |  |  |  |  |  |  | Exclusive breastfeeding duration:  ≥ 6 vs < 2 months or none |  |  |  |  |  |  |  |  | 1.24 (0.46 to 3.36) | age, sex, age at introduction of dairy products |  |
| Virtanen 1994a^110^ | Finland | Childhood Diabetes in Finland  (DiMe) | Case-control | 172 | 86 | < 7 years | Breastfeeding duration:  ≥ 4 vs < 4 months | Questionnaire | Infancy | | T1D | Clinical diagnosis | No | No | No | 0.54 (0.21 to 1.37) | age, sex | Serious |
|  |  |  |  |  |  |  | Age at cow's milk introduction:  ≥ 4 vs < 4 months |  | Infancy | |  |  |  |  |  | 0.58 (0.30 to 1.14) |  |  |
|  |  |  |  |  |  |  | Intake of cow's milk: ≥ 3 vs < 3 glasses/day |  | Period before diagnosis | |  |  |  |  |  | 1.10 (0.60 to 2.00) |  |  |

^*^ This column refers to the presence of islet autoimmunity at baseline where ‘Yes’ indicates islet autoimmunity at baseline, ‘No’ indicates no islet autoimmunity or no assessment of islet autoimmunity at baseline, and ‘Some’ indicates that some individuals had islet autoimmunity at baseline. IA, islet autoimmunity; T1D, type 1 diabetes; HLA, human leukocyte antigen; CI, confidence interval; SD, standard deviation; FFQ, food frequency questionnaire; ALA, alpha-linolenic acid; EPA, eicosapentaenoic acid; DHA, docosahexaenoic acid; DPA, docosapentaenoic acid; LA, linoleic acid; ARA, arachidonic acid; GLA, gamma-linolenic acid

**Supplementary Table 9. Characteristics of associations that could not be meta-analyzed**

| **Study** | **Country** | **Cohort name** | **Study design** | **N  total** | | **N cases** | **Age at diagnosis** | **Exposure** | | | **Outcome** | | | | **IA**^*^ | | **HLA risk** | **Family history** | **Relative risk (95% CI)** | **Adjusted factors** | | **Risk of bias** | **Exclusion^†^** |
| --- | --- | --- | --- | --- | --- | --- | --- | --- | --- | --- | --- | --- | --- | --- | --- | --- | --- | --- | --- | --- | --- | --- | --- |
|  |  |  |  |  |  |  |  | Assessment | | Time |  |  |  |  |  |  |  |  |  |  |  |  |  |
| **Breastfeeding duration** | | | | | | | | | | | | | | | | | | | | | | |  |
| Glatthaar 1988^19^ | Australia |  | Case-control | 947 | 194 | | < 18 years | Breastfeeding duration:  < 1 week vs ≥ 1 week | Questionnaire | Infancy | | T1D | self-reported, cross-checked with health records | | No | | No | Some | 1.40 (1.00 to 1.95) | age, sex | | Serious | Cut-off |
| **Introduction to foods** | | | | | | | | | | | | | | | | | | | | | | |  |
| Lamb 2008a^33^ | USA | DAISY | Cohort | 642 | | 27 | 4 years | Age at cereal introduction: < 3 months vs 4-6 months | telephone interview | Infancy | | IA | | Radioimmunoassays | | No | Some | Some | 2.12 (0.81 to 5.57) | | total calories of the maternal diet, ethnicity, human leukocyte antigen genotype of the child, whether or not the child has a first-degree relative with type 1 diabetes mellitus, potato consumption during pregnancy, duration of breast-feeding | Moderate | Same cohort |
| Lamb 2008a^33^ | USA | DAISY | Cohort | 642 | | 27 | 4 years | Age at cereal introduction: > 7 months vs 4-6 months | telephone interview | Infancy | | IA | | Radioimmunoassays | | No | Some | Some | 6.08 (1.94 to 19.08) | | total calories of the maternal diet, ethnicity, human leukocyte antigen genotype of the child, whether or not the child has a first-degree relative with type 1 diabetes mellitus, potato consumption during pregnancy, duration of breast-feeding | Moderate | Same cohort |
| Hummel 2017^24^ | USA & Europe | TEDDY | Cohort | 8506 | | 686 | < 8 years | Type of first formula introduced during first 3 months: Cow’s milk–based, extensively hydrolyzed vs non-hydrolyzed | Questionnaire | Infancy | | IA | | Lab measurement (no details) | | No | Yes | Some | 1.38 (0.95 to 2.01) | | HLA genotype, first-degree relative with type 1 diabetes, mother with type 1 diabetes, sex, country, mode of delivery, any breastfeeding at 3 months, and seasonality of birth | Moderate | Unique |
| Uusitalo U 2018^81^ | USA & Europe | TEDDY | Cohort | 7525 | | 701 | 33 months (16-62) | Age at cow’s milk introduction: per 1 month delay | food diary | Infancy | | IA | | Radio binding assays | | No | Yes | Some | 1.01 (0.98 to 1.04) | | age, sex, country, HLA genotype, FDR status, sex of the child, and probiotic use < 28 days | Moderate | Same cohort |
| Uusitalo U 2018^81^ | USA & Europe | TEDDY | Cohort | 7142 | | 684 | 33 months (16-62) | Age at egg introduction: per 1 month delay | food diary | Infancy | | IA | | Radio binding assays | | No | Yes | Some | 1.02 (0.99 to 1.05) | | country, HLA genotype, FDR status, sex of the child, and probiotic use < 28 days | Moderate | Unique |
| Skrodeniené 2010^71^ | Lithuania | DEBS | Case-control | 202 | | 124 | 9.2 ± 3.9 years | Age at egg introduction: < 5 months vs ≥ 5 months | Questionnaire | Infancy | | T1D | | Clinical diagnosis | | No | Some | No | 3.70 (1.77 to 7.75) | | mother’s residence during pregnancy (village or remote house), tetanus, diphtheria, pertussis vaccine, varicella infection, rubella infection, infection during the last 6 months before diagnosis of T1D, stressful event previous 6 months before diagnosis | Serious | Unique |
| Uusitalo U 2016^111^ | USA & Europe | TEDDY | Cohort | 7473 | | 601 | 33 ± 23 months | Age at probiotics introduction: < 28 days vs > 1 year or no exposure | Questionnaire | Infancy | | IA | | Radio binding assays | | No | Yes | Some | 0.66 (0.45 to 0.96) | | FDR status, HLA-DR genotype, sex, birth order, mode of delivery, exclusive breastfeeding duration, birth year, child antibiotic use, diarrhea, maternal age, maternal probiotic use, and maternal smoking during pregnancy | Moderate | Unique |
| Krischer 2017^112^ | USA and Europe | TEDDY | Cohort | 8503 | | 589 | 27 months (14.9-43.2) | Age at probiotics introduction: < 28 days vs ≥ 28 days | Questionnaire | Infancy | | IA | | Radio binding assays | | No | Yes | Some | 0.66 (0.46 to 0.96) | | sex, family history, HLA, SNPs, country of residence, weight at 12 months, health conditions before first clinical visit | Moderate | Unique |
| Krischer 2017^112^ | USA and Europe | TEDDY | Cohort | 8504 | | 172 | 38.5 months (24.0-56.1) | Age at probiotics introduction: < 28 days vs ≥ 28 days | Questionnaire | Infancy | | T1D | | Clinical diagnosis | | No | Yes | Some | 0.86 (0.49 to 1.53) | | birth year, season, sex, HLA‐DQB1 genotype, birth data, and maternal age at delivery | Moderate | Unique |
| Lund-Blix 2015^37^ | Norway | MIDIA | Cohort | 726 | | 25 |  | Age at fish introduction: < 8 months vs 8-8.9 months | Questionnaire | Infancy | | T1D | | Clinical diagnosis | | Some | Yes | Some | 2.01 (0.46 to 8.84) | | first-degree relative with type 1 diabetes, vitamin D supplementation, maternal education level, sex, and delivery type | Moderate | Unique |
| **Intake of foods** | | | | | | | | | | | | | | | | | | | | | | |  |
| Hakola 2019^22^ | Finland | DIPP | Cohort | 5714 | | 90 | < 6 years | Cereal intake (with gluten): per 10g increase | 3-day food record | < 6 years old | | T1D | | Clinical diagnosis | | No | Yes | Some | 1.16 (1.01 to 1.34) | | sex of the child, human leukocyte antigen genotype, and familial  diabetes. | Moderate | Unique |
| Hakola 2019^22^ | Finland | DIPP | Cohort | 5545 | | 246 | < 6 years | Cereal intake (with gluten): per 10g increase | 3-day food record | < 6 years old | | IA | | Radio binding assays | | No | Yes | Some | 1.11 (1.03 to 1.20) | | sex of the child, human leukocyte antigen genotype, and familial  diabetes. | Moderate | Unique |
| Lamb 2008a^33^ | USA | DAISY | Cohort | 642 | | 27 | 4 years | Cereal intake (without gluten): per 1 SD increase | semi-quantitative FFQ | Fetal (3d trimester) | | IA | | Radioimmunoassays | | No | Some | Some | 0.98 (0.64 to 1.51) | | total calories of the maternal diet, ethnicity, human leukocyte antigen genotype of the child, and whether or not the child has a first-degree relative with type 1 diabetes mellitus | Moderate | Unique |
| Koivusaari 2020^29^ | Finland | DIPP | Cohort | 5545 | | 246 | 2.5 years (1.3- 3.6) | Intake of milk-based infant formulas not hydrolized: per 100g increase | 3-day food records | Infancy | | IA | | Islet cell autoantibodies (ICAs) with immunofluorescence | | No | Yes | Some | 1.02 (0.88 to 1.19) | | child’s sex, genetic risk and first-degree familial diabetes | Moderate | Unique |
| Niinistö 2014^50^ | Finland | DIPP | Cohort | 4887 | | 112 | 4.6 years (0.5-11.5) | Intake of cow’s milk products: per 1 SD increase | semi-quantitative FFQ | Fetal (8^th^ month) | | T1D | | Immunofluorescence, microassay, radio binding assays | | Some | Yes | Some | 1.01 (0.81 to 1.13) | | maternal energy intake, genetic risk, hospital of birth, familial diabetes and maternal vocational education | Moderate | Unique |
| Dahlquist 1991^14^ | Sweden | The Swedish childhood diabetes study | Case-control | 867 | | 339 | < 14 years | Intake of cow’s milk: <25^th^ centile vs > 25^th^ centile | Questionnaire |  | | T1D | | Diabetes registry | | No | No | Some | 2.03 (1.34 to 3.07) | | familial type 1 diabetes, familial type 2 diabetes, maternal age ≥ 40), maternal education (< university), breasrfeeding duration, foods rich in protein, m, nitrosamines, no measles vaccination, frequency of infections last year (> 1), increased height, stressful life events last year (> 0) | Serious | Cut-off |
| Niinistö 2015^51^ | Finland | DIPP | Cohort | 2939 | | 172 | 4.4 years (0.5-13.0) | Intake of cow’s milk products: per 1 SD increase | semi-quantitative FFQ | Lactation (3d month) | | IA | | Immunofluorescence, microassay, radio binding assays | | No | Yes | Some | 1.07 (0.89 to 1.29) | | maternal energy intake, genetic risk group (0 = moderate), familial diabetes (0 = no), maternal vocational education (1 = none) and duration of exclusive breastfeeding | Serious | Unique |
| Niinistö 2015^51^ | Finland | DIPP | Cohort | 2939 | | 81 | 6.1 years (1.3-13.2) | Intake of cow’s milk products: per 1 SD increase | semi-quantitative FFQ | Lactation (3d month) | | T1D | | Clinical diagnosis | | Some | Yes | Some | 0.93 (0.71 to 1.21) | | maternal energy intake, genetic risk group (0 = moderate), familial diabetes (0 = no), maternal vocational education (1 = none) and duration of exclusive breastfeeding | Serious | Unique |
| Benson 2008^6^ | Canada |  | Case-control | 162 | | 57 | 9 years (4-13) | Intake of cheese: more than daily vs less than daily | FFQ | Year before diagnosis/interview | | T1D | | Clinical diagnosis | | No | No | Some | 0.22 (0.05 to 1.03) | | age, sex, type of FFQ | Moderate | Unique |
| Koivusaari 2020^29^ | Finland | DIPP | Cohort | 5545 | | 246 | 2.5 years (1.3- 3.6) | Intake of cheese: per 100g increase | 3-day food records | < 6 | | IA | | Islet cell autoantibodies (ICAs) with immunofluorescence | | No | Yes | Some | 1.57 (0.30 to 8.23) | | child’s sex, genetic risk and first-degree familial diabetes | Moderate | Unique |
| Niinistö 2014^50^ | Finland | DIPP | Cohort | 4887 | | 240 | 3.6 years (0.5-10) | Intake of cheese: > 86 g/d vs 31-86 g/d | semi-quantitative FFQ | Fetal (8^th^ month) | | IA | | Immunofluorescence, microassay, radio binding assays | | No | Yes | Some | 0.74 (0.52 to 1.04) | | maternal energy intake, genetic risk, hospital of birth, familial diabetes and maternal vocational education | Moderate | Unique |
| Niinistö 2014^50^ | Finland | DIPP | Cohort | 4887 | | 112 | 4.6 years (0.5-11.5) | Intake of cheese: > 86 g/d vs 31-86 g/d | semi-quantitative FFQ | Fetal (8^th^ month) | | T1D | | Immunofluorescence, microassay, radio binding assays | | Some | Yes | Some | 0.55 (0.33 to 0.94) | | maternal energy intake, genetic risk, hospital of birth, familial diabetes and maternal vocational education | Moderate | Unique |
| Syrjälä 2019^75^ | Finland | DIPP | Cohort | 5545 | | 348 | 3.5 years (1.8–6.6) | Intake of eggs: per 10g increase | 3-day food records | Annually until the age of 6 | | IA | | Immunofluorescence, radio binding assays | | No | Yes | Some | 1.08 (0.91 to 1.29) | | sex of the child, genetic risk of the child and familial diabetes | Moderate | Unique |
| Niinistö 2014^50^ | Finland | DIPP | Cohort | 4887 | | 112 | 4.6 years (0.5-11.5) | Intake of fatty fish: per 1 SD increase | semi-quantitative FFQ | Fetal (8^th^ month of pregnancy) | | T1D | | Clinical diagnosis | | Some | Yes | Some | 0.91 (0.73 to 1.14) | | maternal energy intake, genetic risk, hospital of birth, familial diabetes and maternal vocational education | Moderate | Unique |
| Niinistö 2014^50^ | Finland | DIPP | Cohort | 4887 | | 112 | 4.6 years (0.5-11.5) | Intake of lean fish: per 1 SD increase | semi-quantitative FFQ | Fetal (8^th^ month of pregnancy) | | T1D | | Clinical diagnosis | | Some | Yes | Some | 0.99 (0.81 to 1.20) | | maternal energy intake, genetic risk, hospital of birth, familial diabetes and maternal vocational education | Moderate | Unique |
| Niinistö 2015^51^ | Finland | DIPP | Cohort | 2939 | | 172 | 4.4 years (0.5-13.0) | Intake of fatty fish: < 0.5 times/week vs 0.5 to 1.5 times/week | semi-quantitative FFQ | Lactation (3d month) | | IA | | Immunofluorescence, microassay, radio binding assays | | No | Yes | Some | 0.92 (0.63 to 1.33) | | maternal energy intake, genetic risk group (0 = moderate), familial diabetes (0 = no), maternal vocational education (1 = none) and duration of exclusive breastfeeding | Serious | Unique |
| Niinistö 2015^51^ | Finland | DIPP | Cohort | 2939 | | 172 | 4.4 years (0.5-13.0) | Intake of lean fish: < 0.2 times/week vs 0.2 to 0.9 times/week | semi-quantitative FFQ | Lactation (3d month) | | IA | | Immunofluorescence, microassay, radio binding assays | | No | Yes | Some | 0.73 (0.51 to 1.04) | | maternal energy intake, genetic risk group (0 = moderate), familial diabetes (0 = no), maternal vocational education (1 = none) and duration of exclusive breastfeeding | Serious | Unique |
| Niinistö 2015^51^ | Finland | DIPP | Cohort | 2939 | | 81 | 6.1 years (1.3-13.2) | Intake of fatty fish: < 5 times/week vs 0.5 to 1.5 times/week | semi-quantitative FFQ | Lactation (3d month) | | T1D | | Clinical diagnosis | | Some | Yes | Some | 0.97 (0.59 to 1.58) | | maternal energy intake, genetic risk group (0 = moderate), familial diabetes (0 = no), maternal vocational education (1 = none) and duration of exclusive breastfeeding | Serious | Unique |
| Niinistö 2015^51^ | Finland | DIPP | Cohort | 2939 | | 81 | 6.1 years (1.3-13.2) | Intake of lean fish: < 0.2 times/week vs 0.2 to 0.9 times/week | semi-quantitative FFQ | Lactation (3d month) | | T1D | | Clinical diagnosis | | Some | Yes | Some | 0.63 (0.38 to 1.03) | | maternal energy intake, genetic risk group (0 = moderate), familial diabetes (0 = no), maternal vocational education (1 = none) and duration of exclusive breastfeeding | Serious | Unique |
| Syrjälä 2019^75^ | Finland | DIPP | Cohort | 5545 | | 348 | 3.5 years (IQR: 1.8–6.6) | Intake of fish and fish products: per 10g increase | 3-day food records | Annually until the age of 6 | | IA | | Immunofluorescence, radio binding assays | | No | Yes | Some | 0.99 (0.86 to 1.14) | | sex of the child, genetic risk of the child and familial diabetes | Moderate | Unique |
| Niinistö 2014^50^ | Finland | DIPP | Cohort | 4887 | | 112 | 4.6 years (0.5-11.5) | Intake of meat and meat products: per 1 SD increase | semi-quantitative FFQ | Fetal (8^th^ month) | | T1D | | Immunofluorescence, microassay, radio binding assays | | Some | Yes | Some | 1.16 (0.92 to 1.45) | | maternal energy intake, genetic risk, hospital of birth, familial diabetes and maternal vocational education | Moderate | Unique |
| Niinistö 2015^51^ | Finland | DIPP | Cohort | 2939 | | 172 | 4.4 years (0.5-13.0) | Intake of red meat and meat products: per 1 SD increase | semi-quantitative FFQ | Lactation (3d month) | | IA | | Immunofluorescence, microassay, radio binding assays | | No | Yes | Some | 1.19 (1.02 to 1.40) | | maternal energy intake, genetic risk group (0 = moderate), familial diabetes (0 = no), maternal vocational education (1 = none) and duration of exclusive breastfeeding | Serious | Unique |
| Niinistö 2015^51^ | Finland | DIPP | Cohort | 2939 | | 172 | 4.4 years (0.5-13.0) | Intake of processed meat: per 1 SD increase | semi-quantitative FFQ | Lactation (3d month) | | IA | | Immunofluorescence, microassay, radio binding assays | | No | Yes | Some | 1.12 (0.96 to 1.32) | | maternal energy intake, genetic risk group (0 = moderate), familial diabetes (0 = no), maternal vocational education (1 = none) and duration of exclusive breastfeeding | Serious | Unique |
| Niinistö 2015^51^ | Finland | DIPP | Cohort | 2939 | | 81 | 6.1 years (1.3-13.2) | Intake of red meat and meat products: per 1 SD increase | semi-quantitative FFQ | Lactation (3d month) | | T1D | | Clinical diagnosis | | Some | Yes | Some | 1.27 (1.06 to 1.52) | | maternal energy intake, genetic risk group (0 = moderate), familial diabetes (0 = no), maternal vocational education (1 = none) and duration of exclusive breastfeeding | Serious | Unique |
| Niinistö 2015^51^ | Finland | DIPP | Cohort | 2939 | | 81 | 6.1 years (1.3-13.2) | Intake of processed meat: per 1 SD increase | semi-quantitative FFQ | Lactation (3d month) | | T1D | | Clinical diagnosis | | Some | Yes | Some | 1.23 (1.02 to 1.48) | | maternal energy intake, genetic risk group (0 = moderate), familial diabetes (0 = no), maternal vocational education (1 = none) and duration of exclusive breastfeeding | Serious | Unique |
| Syrjälä 2019^75^ | Finland | DIPP | Cohort | 5545 | | 348 | 3.5 years (1.8–6.6) | Intake of red meat and meat products: per 10g increase | 3-day food records | Annually until the age of 6 | | IA | | Immunofluorescence, radio binding assays | | No | Yes | Some | 1.01 (0.96 to 1.05) | | sex of the child, genetic risk of the child and familial diabetes | Moderate | Unique |
| Awadalla 2017^4^ | Egypt |  | Hospital-based case-control | 408 | | 204 | 7.94 ± 3.39 years | Intake of vegetables: > 4 times/week vs < 4 times/week | specific question | Child | | T1D | | Clinical diagnosis | | No | No | No | 0.24 (0.09 to 0.70) | | age, sex | Serious | Unique |
| **Intake of drinks** | | | | | | | | | | | | | | | | | | | | | | |  |
| Virtanen 1994b^87^ | Finland | DiMe | Case-control | 1136 | | 600 | < 14 years | Intake of tea: ≥ 2 cups (110 ml)/day vs < 2 cups | Questionnaire | Before diagnosis | | T1D | | Clinical diagnosis | | No | No | No | 2.59 (1.60 to 4.18) | | mother's education (< 14 years, ≥ 14 years), child's age, sex | Serious | Unique |
| Lamb 2015a^35^ | USA | DAISY | Cohort | 1893 | | 139 | 6.1 years (3.5-10.1) | Intake of fruit juice: 7.2 servings per week | semi-quantitative FFQ | Annually after the age of 2 | | IA | | Radioimmunoassays | | No | Some | Some | 0.93 (0.77 to 1.13) | | total energy, FFQ type, HLA-DR,DQ genotype, family history of type 1 diabetes and ethnicity | Moderate | Unique |
| Lamb 2015a^35^ | USA | DAISY | Cohort | 1893 | | 139 | 6.1 years (3.5-10.1) | Intake of sugar sweetened beverages: per 4.8 servings per week | semi-quantitative FFQ | Annually after the age of 2 | | IA | | Radioimmunoassays | | No | Some | Some | 1.08 (0.92 to 1.26) | | total energy, FFQ type, HLA-DR,DQ genotype, family history of type 1 diabetes and ethnicity | Moderate | Unique |
| Benson 2010^7^ | Canada |  | Case-control | 159 | | 55 | < 18 years | Intake of alcohol: any vs none | FFQ | Year before diagnosis/interview | | T1D | | Diabetes registry | | No | No | No | 0.45 (0.08 to 2.43) | | age, sex, and energy intake (Kcal), a third-generation family member previously diagnosed with T1D (siblings, parents, grandparents, parental siblings, and cousins), the number of infections during the first 2 years of life, residential area (urban, rural or farm), and father’s education level | Moderate | Unique |
| **Intake of nutrients** | | | | | | | | | | | | | | | | | | | | | | |  |
| Lamb 2008b^34^ | USA | DAISY | Cohort | 1776 | | 79 | 4.82 ± 2.49 years | Intake of carbohydrates: per 1 SD increase (100g) | semi-quantitative FFQ | Annually after the age of 2 | | IA | | Radioimmunoassays | | No | Some | Some | 0.91 (0.73 to 1.15) | | total calories, HLA genotype, and family history of type 1 diabetes | Moderate | Unique |
| Lamb 2008b^34^ | USA | DAISY | Cohort | 89 | | 17 | 6.22 ± 2.51 years | Intake of carbohydrates: per 1 SD increase (100g) | semi-quantitative FFQ | Annually after the age of 2 | | T1D | | Clinical diagnosis | | Yes | Some | Some | 1.44 (0.86 to 2.41) | | total calories, HLA genotype, age at first autoantibody positive visit, and family history of type 1 diabetes | Moderate | Same cohort |
| Lamb 2015a^35^ | USA | DAISY | Cohort | 1893 | | 139 | 6.1 years (3.5-10.1) | Intake of carbohydrates: per 99.9g | semi-quantitative FFQ | Annually after the age of 2 | | IA | | Radioimmunoassays | | No | Some | Some | 0.84 (0.50 to 1.41) | | total energy, FFQ type, HLA-DR,DQ genotype, family history of type 1 diabetes and ethnicity | Moderate | Unique |
| Lamb 2015a^35^ | USA | DAISY | Cohort | 1893 | | 139 | 6.1 years (3.5-10.1) | Intake of sugar: per 58.1g | semi-quantitative FFQ | Annually after the age of 2 | | IA | | Radioimmunoassays | | No | Some | Some | 1.01 (0.75 to 1.36) | | total energy, FFQ type, HLA-DR, DQ genotype, family history of type 1 diabetes and ethnicity | Moderate | Unique |
| Pundziūtė-Luckå 2004^58^ | Sweden |  | Case-control | 279 | | 99 | 7-14 years | Intake of fat: >75^th^ percentile vs ≤ 75^th^ percentile | FFQ | Year before diagnosis/interview | | T1D | | Clinical diagnosis | | No | No | No | 1.10 (0.54 to 2.23) | | age, sex, geographical region within Stockholm, energy intake | Moderate | Unique |
| Lund-Blix 2020^40^ | Norway | MoBa | Cohort | 86306 | | 346 | 7.5 years (0.7-15.0) | Intake of fiber: per 1g/d increase | semi-quantitative FFQ | Fetal (week 22 of pregnancy) | | T1D | | Clinical diagnosis | | No | No | Some | 1.00 (0.99 to 1.01) | | maternal age, pre-pregnant maternal body mass index, parity, smoking during pregnancy, education, caesarean section, breastfeeding, sex, energy intake, birthweight, age at gluten introduction, prematurity, fibre intake, weight gain 0-12 months | Moderate | Unique |
| Uusitalo L 2008b^113^ | Finland | DIPP | Cohort | 3723 | | 138 | 4.4 (0.2-8.8) | Retinol intake from foods and supplements: per 2-fold increase | semi-quantitative FFQ | Fetal (8^th^ month) | | IA | | Immunofluorescence, microassay, radio binding assays | | No | Yes | Some | 1.03 (0.81 to 1.30) | | genetic risk, familial diabetes, sex, gestational age, maternal age, maternal parity, maternal education, maternal smoking during pregnancy, degree of urbanization of the home municipality, and region of birth (Oulu versus Tampere area) | Moderate | Unique |
| Uusitalo L 2008b^113^ | Finland | DIPP | Cohort | 3723 | | 138 | 4.4 (0.2-8.8) | Beta-carotene intake from foods and supplements: per 2-fold increase | semi-quantitative FFQ | Fetal (8^th^ month) | | IA | | Immunofluorescence, microassay, radio binding assays | | No | Yes | Some | 1.02 (0.85 to 1.22) | | genetic risk, familial diabetes, sex, gestational age, maternal age, maternal parity, maternal education, maternal smoking during pregnancy, degree of urbanization of the home municipality, and region of birth (Oulu versus Tampere area) | Moderate | Unique |
| Benson 2010^7^ | Canada |  | Case-control | 159 | | 55 | < 18 years | Intake of thiamine: ≥ 2.08 mg/d vs < 1.31 | FFQ | Year before diagnosis/interview | | T1D | | Diabetes registry | | No | No | No | 1.26 (0.19 to 8.17) | | age, sex, and energy intake (Kcal), a third-generation family member previously diagnosed with T1D (siblings, parents, grandparents, parental siblings, and cousins), the number of infections during the first 2 years of life, residential area (urban, rural or farm), and father’s education level | Moderate | Unique |
| Benson 2010^7^ | Canada |  | Case-control | 159 | | 55 | < 18 years | Intake of niacin: ≥ 43.9 (NE) vs < 29.3 | FFQ | Year before diagnosis/interview | | T1D | | Diabetes registry | | No | No | No | 0.93 (0.17 to 5.15) | | age, sex, and energy intake (Kcal), a third-generation family member previously diagnosed with T1D (siblings, parents, grandparents, parental siblings, and cousins), the number of infections during the first 2 years of life, residential area (urban, rural or farm), and father’s education level | Moderate | Unique |
| Benson 2010^7^ | Canada |  | Case-control | 159 | | 55 | < 18 years | Intake of riboflavin: ≥ 2.96 mg/d vs < 2.01 | FFQ | Year before diagnosis/interview | | T1D | | Diabetes registry | | No | No | No | 0.52 (0.10 to 2.59) | | age, sex, and energy intake (Kcal), a third-generation family member previously diagnosed with T1D (siblings, parents, grandparents, parental siblings, and cousins), the number of infections during the first 2 years of life, residential area (urban, rural or farm), and father’s education level | Moderate | Unique |
| Benson 2010^7^ | Canada |  | Case-control | 159 | | 55 | < 18 years | Intake of B6 vitamin: ≥ 1.96 mg/d vs < 1.31 | FFQ | Year before diagnosis/interview | | T1D | | Diabetes registry | | No | No | No | 1.02 (0.19 to 5.59) | | age, sex, and energy intake (Kcal), a third-generation family member previously diagnosed with T1D (siblings, parents, grandparents, parental siblings, and cousins), the number of infections during the first 2 years of life, residential area (urban, rural or farm), and father’s education level | Moderate | Unique |
| Benson 2010^7^ | Canada |  | Case-control | 159 | | 55 | < 18 years | Intake of folate: ≥ 544.3 microg/d vs < 333.2 | FFQ | Year before diagnosis/interview | | T1D | | Diabetes registry | | No | No | No | 0.28 (0.05 to 1.58) | | age, sex, and energy intake (Kcal), a third-generation family member previously diagnosed with T1D (siblings, parents, grandparents, parental siblings, and cousins), the number of infections during the first 2 years of life, residential area (urban, rural or farm), and father’s education level | Moderate | Unique |
| Benson 2010^7^ | Canada |  | Case-control | 159 | | 55 | < 18 years | Intake of B12 vitamin: ≥ 6.24 microg/d vs < 4.09 | FFQ | Year before diagnosis/interview | | T1D | | Diabetes registry | | No | No | No | 0.85 (0.23 to 3.18) | | age, sex, and energy intake (Kcal), a third-generation family member previously diagnosed with T1D (siblings, parents, grandparents, parental siblings, and cousins), the number of infections during the first 2 years of life, residential area (urban, rural or farm), and father’s education level | Moderate | Unique |
| Uusitalo L 2008b^113^ | Finland | DIPP | Cohort | 3723 | | 138 | 4.4 (0.2-8.8) | Vitamin C intake from foods and supplements: per 2-fold increase | semi-quantitative FFQ | Fetal (8^th^ month of pregnancy) | | IA | | Immunofluorescence, microassay, radio binding assays | | No | Yes | Some | 0.96 (0.78 to 1.17) | | genetic risk, familial diabetes, sex, gestational age, maternal age, maternal parity, maternal education, maternal smoking during pregnancy, degree of urbanization of the home municipality, and region of birth (Oulu versus Tampere area) | Moderate | Unique |
| Fronczak 2003^114^ | USA | DAISY | Cohort | 222 | | 16 | 2.5 ± 1.7 years | Vitamin D intake via food: per 155.6 IU | FFQ | Fetal (3d trimester) | | IA | | Radioimmunoassays | | No | Some | Some | 0.37 (0.17 to 0.78) | | family history of T1D, HLA genotype, gestational diabetes, ethnicity | Moderate | Unique |
| Benson 2010^7^ | Canada |  | Case-control | 159 | | 55 | < 18 years | Intake of vitamin D: ≥ 10.87 microg/d vs < 5.14 | FFQ | Year before diagnosis/interview | | T1D | | Diabetes registry | | No | No | No | 0.65 (0.17 to 2.44) | | age, sex, and energy intake (Kcal), a third-generation family member previously diagnosed with T1D (siblings, parents, grandparents, parental siblings, and cousins), the number of infections during the first 2 years of life, residential area (urban, rural or farm), and father’s education level | Moderate | Unique |
| Marjamäki 2010^115^ | Finland | DIPP | Cohort | 3395 | | 138 | < 4 years | Intake of vitamin D: 4^th^ quartile vs 1^st^ quartile | FFQ | Fetal | | IA | | Radio binding assays | | No | Yes | Some | 1.17 (0.73 to 1.90) | | genetic risk, familial type 1 diabetes, sex, gestational age, maternal age, maternal education, delivery hospital, route of delivery, number of earlier deliveries and smoking during pregnancy | Moderate | Unique |
| Marjamäki 2010^115^ | Finland | DIPP | Cohort | 3395 | | 138 | < 4 years | Vitamin D supplementation: continuous | FFQ | Fetal | | IA | | Radiobinding assays | | No | Yes | Some | 1.05 (0.95 to 1.16) | | genetic risk, familial type 1 diabetes, sex, gestational age, maternal age, maternal education, delivery hospital, route of delivery, number of earlier deliveries and smoking during pregnancy | Moderate | Unique |
| Marjamäki 2010^115^ | Finland | DIPP | Cohort | 3723 | | 55 | 4.1 years (1.0-7.9) | Intake of vitamin D: 4^th^ quartile vs 1^st^ quartile | FFQ | Fetal | | T1D | | Clinical diagnosisWHO criteria | | No | Yes | Some | 0.77 (0.35 to 1.72) | | genetic risk, familial type 1 diabetes, sex, gestational age, maternal age, maternal education, delivery hospital, route of delivery, number of earlier deliveries and smoking during pregnancy | Moderate | Unique |
| Marjamäki 2010^115^ | Finland | DIPP | Cohort | 3723 | | 55 | 4.1 years (1.0-7.9) | Vitamin D supplementation: continuous | FFQ | Fetal | | T1D | | Clinical diagnosisWHO criteria | | No | Yes | Some | 1.09 (0.99 to 1.20) | | genetic risk, familial type 1 diabetes, sex, gestational age, maternal age, maternal education, delivery hospital, route of delivery, number of earlier deliveries and smoking during pregnancy | Moderate | Unique |
| Simpson 2011^66^ | USA | DAISY | Cohort | 1875 | | 123 | 6.4 ± 3.4 years | Intake of vitamin D: per 1 SD increase (159.39 IU) | FFQ | Anually after the age of 2 | | IA | | Radioimmunoassays | | No | Some | Some | 1.15 (0.94 to 1.41) | | family history of T1D, HLA-DR3/4, DQB1*0302 genotype, reported energy intake, and the type of survey completed | Moderate | Unique |
| Simpson 2011^66^ | USA | DAISY | Cohort | 1875 | | 123 | 6.4 ± 3.4 years | Vitamin D supplementation: per 1 SD increase (141.01 IU) | FFQ | Anually after the age of 2 | | IA | | Radioimmunoassays | | No | Some | Some | 1.05 (0.89 to 1.23) | | family history of T1D, HLA-DR3/4, DQB1*0302 genotype, reported energy intake, and the type of survey completed | Moderate | Unique |
| Simpson 2011^66^ | USA | DAISY | Cohort | 178 | | 35 | 8.9 ± 4.0 years | Intake of vitamin D: per 1 SD increase (156.04 IU) | FFQ | Anually since IA diagnosis | | T1D | | Clinical diagnosis | | Yes | Some | Some | 1.16 (0.77 to 1.76) | | family history of T1D, HLA-DR3/4, DQB1*0302 genotype, reported energy intake, the type of survey completed and age at first appearance of autoantibodies | Moderate | Unique |
| Simpson 2011^66^ | USA | DAISY | Cohort | 178 | | 35 | 8.9 ± 4.0 years | Vitamin D supplementation: per 1 SD increase (147.15 IU) | FFQ | Anually since IA diagnosis | | T1D | | Clinical diagnosis | | Yes | Some | Some | 1.18 (0.89 to 1.58) | | family history of T1D, HLA-DR3/4, DQB1*0302 genotype, reported energy intake, the type of survey completed and age at first appearance of autoantibodies | Moderate | Unique |
| Uusitalo L 2008b^113^ | Finland | DIPP | Cohort | 3723 | | 138 | 4.4 (0.2-8.8) | Intake of vitamin E from foods and supplements: per 2-fold increase | semi-quantitative FFQ | Fetal (8^th^ month) | | IA | | Immunofluorescence, microassay, radio binding assays | | No | Yes | Some | 1.06 (0.75 to 1.49) | | genetic risk, familial diabetes, sex, gestational age, maternal age, maternal parity, maternal education, maternal smoking during pregnancy, degree of urbanization of the home municipality, and region of birth (Oulu versus Tampere area) | Moderate | Unique |
| Benson 2010^7^ | Canada |  | Case-control | 159 | | 55 | <18 years | Intake of vitamin E: ≥ 5.36 mg/d vs < 3.21 | FFQ | Year before diagnosis/interview | | T1D | | Diabetes registry | | No | No | No | 0.40 (0.08 to 2.06) | | age, sex, and energy intake (Kcal), a third-generation family member previously diagnosed with T1D (siblings, parents, grandparents, parental siblings, and cousins), the number of infections during the first 2 years of life, residential area (urban, rural or farm), and father’s education level | Moderate | Unique |
| Benson 2010^7^ | Canada |  | Case-control | 159 | | 55 | <18 years | Intake of calcium: ≥ 1672.8 mg/d vs < 1024.1 | FFQ | Year before diagnosis/interview | | T1D | | Diabetes registry | | No | No | No | 1.23 (0.29 to 5.24) | | age, sex, and energy intake (Kcal), a third-generation family member previously diagnosed with T1D (siblings, parents, grandparents, parental siblings, and cousins), the number of infections during the first 2 years of life, residential area (urban, rural or farm), and father’s education level | Moderate | Unique |
| Benson 2010^7^ | Canada |  | Case-control | 159 | | 55 | <18 years | Intake of magnesium: ≥ 355 microg/d vs < 228.5 | FFQ | Year before diagnosis/interview | | T1D | | Diabetes registry | | No | No | No | 0.59 (0.10 to 3.66) | | age, sex, and energy intake (Kcal), a third-generation family member previously diagnosed with T1D (siblings, parents, grandparents, parental siblings, and cousins), the number of infections during the first 2 years of life, residential area (urban, rural or farm), and father’s education level | Moderate | Unique |
| Uusitalo L 2008b^113^ | Finland | DIPP | Cohort | 3723 | | 138 | 4.4 (0.2-8.8) | Manganese intake from foods and supplements: per 2-fold increase | semi-quantitative FFQ | Fetal (8^th^ month) | | IA | | Immunofluorescence, microassay, radio binding assays | | No | Yes | Some | 1.03 (0.76 to 1.40) | | genetic risk, familial diabetes, sex, gestational age, maternal age, maternal parity, maternal education, maternal smoking during pregnancy, degree of urbanization of the home municipality, and region of birth (Oulu versus Tampere area) | Moderate | Unique |
| Uusitalo L 2008b^113^ | Finland | DIPP | Cohort | 3723 | | 138 | 4.4 (0.2-8.8) | Selenium intake from foods and supplements: per 2-fold increase | semi-quantitative FFQ | Fetal (8^th^ month) | | IA | | Immunofluorescence, microassay, radio binding assays | | No | Yes | Some | 1.11 (0.76 to 1.64) | | genetic risk, familial diabetes, sex, gestational age, maternal age, maternal parity, maternal education, maternal smoking during pregnancy, degree of urbanization of the home municipality, and region of birth (Oulu versus Tampere area) | Moderate | Unique |
| Uusitalo L 2008b^113^ | Finland | DIPP | Cohort | 3723 | | 138 | 4.4 (0.2-8.8) | Zinc intake from foods and supplements: per 2-fold increase | semi-quantitative FFQ | Fetal (8^th^ month) | | IA | | Immunofluorescence, microassay, radio binding assays | | No | Yes | Some | 1.05 (0.74 to 1.48) | | genetic risk, familial diabetes, sex, gestational age, maternal age, maternal parity, maternal education, maternal smoking during pregnancy, degree of urbanization of the home municipality, and region of birth (Oulu versus Tampere area) | Moderate | Unique |
| Benson 2010^7^ | Canada |  | Case-control | 159 | | 55 | <18 years | Intake of zinc: ≥ 13.13 microg/d vs < 8.47 | FFQ | Year before diagnosis/interview | | T1D | | Diabetes registry | | No | No | No | 0.31 (0.05 to 1.89) | | age, sex, and energy intake (Kcal), a third-generation family member previously diagnosed with T1D (siblings, parents, grandparents, parental siblings, and cousins), the number of infections during the first 2 years of life, residential area (urban, rural or farm), and father’s education level | Moderate | Unique |
| Mattila 2020b^43^ | Finland | DIPP | Cohort | 4706 | | 305 | 3.5 years (1.7–6.6) | Intake of nitrate: per 1 SD (97 mg/d) | semi-quantitative FFQ | Fetal (8^th^ month) | | IA | | Radio binding assays | | No | Yes | Some | 1.00 (0.88 to 1.14) | | energy with residual method, sex, family history of diabetes, human leukocyte antigen genotype, vitamin C, vitamin E, and selenium intakes | Moderate | Unique |
| Mattila 2020b^43^ | Finland | DIPP | Cohort | 4706 | | 305 | 3.5 years (1.7–6.6) | Intake of nitrite: per 1 SD (1 mg/d) | semi-quantitative FFQ | Fetal (8^th^ month) | | IA | | Radio binding assays | | No | Yes | Some | 1.03 (0.92 to 1.16) | | energy with residual method, sex, family history of diabetes, human leukocyte antigen genotype, vitamin C, vitamin E, and selenium intakes | Moderate | Unique |
| **Nutritional biomarker levels in blood** | | | | | | | | | | | | | | | | | | | | | | |  |
| Knekt 1999^116^ | Finland | Finnish Mobile Clinic | Nested case-control | 76  males | | 19 | 26 years (21-46) | Serum alpha-tocopherol levels:  ≥ 8 mg/L vs < 5.2 mg/L | high  performance  liquid chromatography | 4-14 years before diagnosis | | T1D | | Drug registry | | No | No | No | 0.12 (0.02 to 0.85) | | age and time of baseline examination, serum cholesterol | Serious | Unique |
| Uusitalo L 2005^117^ | Finland | DiMe | Nested case-control | 97 | | 16 | 10.2 years (5.1-18.8) | Serum alpha-tocopherol levels:  3d tertile (> 4.45 mg/L) vs ≤ 4.45 mg/L | Reversed-phase high-performance liquid chromatography | prior to diagnosis | | T1D | | National Central Drug Registry | | No | Some | Yes sibling | 0.22 (0.04 to 1.19) | | sex, age, time of serum sample maternal education | Moderate | Unique |
| Uusitalo L 2008a^118^ | Finland | DIPP | Nested case-control | 324 | | 108 | 2.1 (0.18-7.9) | Serum alpha-tocopherol levels: high (> 75th percentile) vs intermediate (25th to 75th percentile) | Fluorescence | Annually until seroconversion | | IA | | Immunofluorescence, microassay, radio binding assays | | No | Yes | Some | 0.80 (0.44 to 1.47) | | birth date, sex, hospital of birth, genotype | Serious | Unique |
| Uusitalo L 2008a^118^ | Finland | DIPP | Nested case-control | 324 | | 108 | 2.1 (0.18-7.9) | Serum gamma-tocopherol levels: high (> 75th percentile) vs intermediate (25th to 75th percentile) | Fluorescence | Annually until seroconversion | | IA | | Immunofluorescence, microassay, radio binding assays | | No | Yes | Some | 1.07 (0.61 to 1.87) | | birth date, sex, hospital of birth, genotype | Serious | Unique |
| Prasad 2011^119^ | Finland | DIPP | Nested case-control | 296 | | 97 | < 6 years | Serum alpha-carotene levels: highest quarter vs intermediate half | Reversed-phase high-performance liquid chromatography | Infancy 12 months | | IA | | Immunofluorescence, microassay, radio binding assays | | No | Yes | Some | 1.21 (0.56 to 2.62) | | birth date, gender, hospital of birth genotype, parental education, maternal age, duration of gestation, diabetes in first-degree relatives, number of earlier deliveries and maternal smoking during pregnancy | Moderate | Unique |
| Prasad 2011^119^ | Finland | DIPP | Nested case-control | 296 | | 97 | < 6 years | Serum beta-carotene levels: highest quarter vs intermediate half | Reversed-phase high-performance liquid chromatography | Infancy 12 months | | IA | | Immunofluorescence, microassay, radio binding assays | | No | Yes | Some | 0.86 (0.39 to 1.89) | | birth date, gender, hospital of birth genotype, parental education, maternal age, duration of gestation, diabetes in first-degree relatives, number of earlier deliveries and maternal smoking during pregnancy | Moderate | Unique |
| Mattila 2020a^120^ | USA & Europe | TEDDY | Case-control | 1324 | | 350 | 23 months (2-72) | Concentration of ascorbic acid in plasma: per 1mg/L increase | Liquid chromatography | Annually until the age of 6 | | IA | | Lab assessment | | No | Some | Some | 0.96 (0.92 to 0.99) | | ethnicity, HLA genotype, family history of T1D, clinical centre, sex | Moderate | Unique |
| Mattila 2020a^120^ | USA & Europe | TEDDY | Case-control | 384 | | 102 | 31 months (8-75) | Concentration of ascorbic acid in plasma: per 1mg/L increase | Liquid chromatography | Annually until the age of 7 | | T1D | | Clinical diagnosis according to the American Diabetes Association criteria | | Some | Some | Some | 0.93 (0.86 to 1.02) | | ethnicity, HLA genotype, family history of T1D, clinical centre, sex | Moderate | Unique |
| Kyvsgaard 2017a^121^ | Denmark |  | Case-control | 398 | | 199 | < 16 years | Zinc levels in blood: lowest vs highest quartile | Laser ablation inductively coupled plasma mass spectrometry | Infancy (1st week) | | T1D | | Clinical diagnosis | | No | Some | No | 0.89 (0.40 to 1.97) | | birth year, season, sex, HLA‐DQB1 genotype, birth data, and maternal age at delivery | Moderate | Unique |
| Kyvsgaard 2017b^122^ | Denmark |  | Case-control | 335 | | 154 | < 16 years | Iron levels in blood: per doubling in iron content | Laser ablation inductively coupled plasma mass spectrometry | Infancy (1st week) | | T1D | | Clinical diagnosis | | No | Some | No | 2.55 (1.04 to 6.24) | | season, birth year, sex, HLA-DQB1 genotype, birth weight, gestational age, and maternal age at delivery | Moderate | Unique |

^*^ This column refers to the presence of islet autoimmunity at baseline where ‘Yes’ indicates islet autoimmunity at baseline, ‘No’ indicates no islet autoimmunity or no assessment of islet autoimmunity at baseline, and ‘Some’ indicates that some individuals had islet autoimmunity at baseline. † This column refers to the reason for excluding the study or association from the meta-analysis where ‘Cut-off’ indicates that the exposure cut-off could not be combined with other cut-offs that were meta-analyzed, ‘Same cohort’ indicates that the estimate was selected from another study based on the same data, and ‘Unique’ indicates that the association under study is unique and could not be synthesized with any other estimate. IA, islet autoimmunity; T1D, type 1 diabetes; HLA, human leukocyte antigen; CI, confidence interval; SD, standard deviation; FFQ, food frequency questionnaire; DAISY, Diabetes Autoimmunity Study in the Young; TEDDY, The Environmental Determinants of Diabetes in the Young; DEBS, Diabetes and Environment around the Baltic Sea; MIDIA, Environmental Triggers of Type 1 Diabetes; DIPP, Finnish Type 1 Diabetes Prediction and Prevention; ABIS, All Babies in Southeast Sweden; DiMe, Childhood Diabetes in Finland; MoBa, Mother and Child Cohort Study

**Supplementary Table 10. Quality of evidence rating using the GRADE system**

| **Certainty assessment** | | | | | | | **№ of cases** | **Exposure details** | | **Summary Relative Risk (95% CI)** | **Certainty** | **Outcome** |
| --- | --- | --- | --- | --- | --- | --- | --- | --- | --- | --- | --- | --- |
| **№ of studies** | **Study design** | **Risk of bias** | **Inconsistency** | **Indirectness** | **Imprecision** | **Other considerations** |  | **Duration/Quantity** | **Timing** |  |  |  |
| **Question: Longer any breastfeeding compared to shorter any breastfeeding for IA and T1D prevention** | | | | | | | | | | | | |
| 3 | cohort | not serious | not serious | not serious | very serious | none | 221 | ≥ 2-4 months vs  < 2-4 months | infancy | **RR 1.04** (0.71 to 1.50) | ⨁⨁◯◯ LOW | IA |
| 3 | cohort | not serious | serious | not serious | not serious | none | 777 | per additional month | infancy | **RR 0.99** (0.94 to 1.04) | ⨁⨁⨁◯ MODERATE | IA |
| 12 | 1 cohort  1 nested case-control 10 case-control | serious | serious | not serious | not serious | none | 3426 | ≥ 2-4 months vs  < 2-4 months | infancy | **RR 0.68** (0.54 to 0.85) | ⨁⨁◯◯ LOW | T1D |
| 4 | 1 cohort 3 case-control | serious | not serious | not serious | not serious | strong association | 565 | ≥ 6-12 months vs  < 6-12 months | infancy | **RR 0.39** (0.26 to 0.58) | ⨁⨁⨁⨁ HIGH | T1D |
| 5 | 3 cohort 2 case-control | serious | serious | not serious | not serious | none | 858 | per additional month | infancy | **RR 0.98** (0.95 to 1.01) | ⨁⨁◯◯ LOW | T1D |
| **Question: Longer exclusive breastfeeding compared to shorter exclusive breastfeeding for IA and T1D prevention** | | | | | | | | | | | | |
| 2 | cohort | serious | not serious | not serious | very serious | none | 185 | ≥ 3 months vs  < 3 months | infancy | **RR 1.11** (0.77 to 1.59) | ⨁◯◯◯ VERY LOW | IA |
| 2 | cohort | not serious | not serious | not serious | serious | none | 759 | per additional month | infancy | **RR 1.00** (0.96 to 1.04) | ⨁⨁⨁◯ MODERATE | IA |
| 8 | case-control | serious | not serious | not serious | not serious | none | 1602 | ≥ 2-3 months vs  < 2-3 months | infancy | **RR 0.68** (0.58 to 0.80) | ⨁⨁⨁◯ MODERATE | T1D |
| 4 | cohort | not serious | not serious | not serious | not serious | none | 582 | per additional month | infancy | **RR 0.97** (0.90 to 1.04) | ⨁⨁⨁⨁ HIGH | T1D |
| **Question: Any breastfeeding compared to no breastfeeding for T1D prevention** | | | | | | | | | | | | |
| 11 | 1 cohort  1 nested case-control 9 case-control | serious | serious | not serious | serious | none | 2398 | any vs none | infancy | **RR 0.86** (0.68 to 1.10) | ⨁◯◯◯ VERY LOW | T1D |
| **Question: Exclusive breastfeeding compared to no exclusive breastfeeding for T1D prevention** | | | | | | | | | | | | |
| 4 | case-control | serious | not serious | not serious | not serious | none | 1241 | any vs none | infancy | **RR 0.65** (0.53 to 0.80) | ⨁⨁⨁◯ MODERATE | T1D |
| **Question: Later introduction to cow's milk compared to earlier introduction to cow's milk for IA and T1D prevention** | | | | | | | | | | | | |
| 5 | cohort | not serious | not serious | not serious | serious | none | 921 | ≥ 2-4 months vs  < 2-4 months | infancy | **RR 1.06** (0.90 to 1.24) | ⨁⨁⨁◯ MODERATE | IA |
| 6 | 5 case-control  1 nested case-control | serious | not serious | not serious | not serious | none | 1704 | ≥ 2-3 months vs  < 2-3 months | infancy | **RR 0.69** (0.59 to 0.81) | ⨁⨁⨁◯ MODERATE | T1D |
| 3 | case-control | serious | serious | not serious | very serious | none | 372 | ≥ 5-7 months vs  < 5-7 months | infancy | **RR 0.65** (0.29 to 1.44) | ⨁◯◯◯ VERY LOW | T1D |
| 2 | case-control | serious | very serious | not serious | very serious | none | 964 | ≥ 1 year vs < 1 year | infancy | **RR 0.20** (0.01 to 2.96) | ⨁◯◯◯ VERY LOW | T1D |
| **Question: Later introduction to infant formula compared to earlier introduction to infant formula for IA and T1D prevention** | | | | | | | | | | | | |
| 2 | cohort | not serious | not serious | not serious | serious | none | 654 | per 1 month delay | infancy | **RR 0.99** (0.97 to 1.02) | ⨁⨁⨁◯ MODERATE | IA |
| 5 | case-control | serious | not serious | not serious | serious | none | 1260 | ≥ 3-4 months vs  < 3-4 months | infancy | **RR 0.73** (0.52 to 1.01) | ⨁⨁◯◯ LOW | T1D |
| 2 | case-control | serious | serious | not serious | very serious | none | 933 | ≥ 4-5 months vs  < 2 weeks | infancy | **RR 0.97** (0.57 to 1.63) | ⨁◯◯◯ VERY LOW | T1D |
| 4 | 3 cohort 1 case-control | not serious | serious | not serious | not serious | none | 725 | per 1 month delay | infancy | **RR 0.97** (0.93 to 1.02) | ⨁⨁⨁◯ MODERATE | T1D |
| **Question: Later introduction to solid food compared to earlier introduction to solid food for IA and T1D prevention** | | | | | | | | | | | | |
| 2 | cohort | not serious | not serious | not serious | serious | none | 757 | per 1 month delay | infancy | **RR 1.02** (0.97 to 1.08) | ⨁⨁⨁◯ MODERATE | IA |
| 8 | 4 cohort 4 case-control | serious | very serious | serious | serious | none | 2101 | ≥ 3-6 months vs  < 3-6 months | infancy | **RR 0.82** (0.53 to 1.27) | ⨁◯◯◯ VERY LOW | T1D |
| **Question: Later introduction to cereal compared to earlier introduction to cereal for IA and T1D prevention** | | | | | | | | | | | | |
| 3 | cohort | serious | very serious | not serious | very serious | none | 191 | ≥ 6 months vs  3-6 months | infancy | **RR 1.62** (0.49 to 5.31) | ⨁◯◯◯ VERY LOW | IA |
| 4 | cohort | not serious | very serious | not serious | very serious | none | 890 | ≥ 4-6 months vs  < 3-5 months | infancy | **RR 0.72** (0.36 to 1.43) | ⨁◯◯◯ VERY LOW | IA |
| 2 | cohort | not serious | serious | not serious | very serious | none | 78 | ≥ 6 months vs  4-6 months | infancy | **RR 2.19** (0.82 to 5.86) | ⨁◯◯◯ VERY LOW | T1D |
| 2 | cohort | not serious | not serious | not serious | very serious | none | 78 | 4-6 months vs  < 4-5 months | infancy | **RR 0.62**  (0.37 to 1.04) | ⨁⨁◯◯ LOW | T1D |
| **Question: Later introduction to gluten compared to earlier introduction to gluten for IA and T1D prevention** | | | | | | | | | | | | |
| 4 | cohort | not serious | not serious | not serious | very serious | none | 460 | ≥ 6 months vs  4-6 months | infancy | **RR 0.89** (0.69 to 1.14) | ⨁⨁◯◯ LOW | IA |
| 5 | cohort | not serious | serious | not serious | very serious | none | 1156 | ≥ 3-6 months vs  < 3-5 months | infancy | **RR 1.05** (0.65 to 1.70) | ⨁◯◯◯ VERY LOW | IA |
| 3 | cohort | not serious | not serious | not serious | very serious | none | 160 | ≥ 6 months vs  4-6 months | infancy | **RR 1.10** (0.74 to 1.64) | ⨁⨁◯◯ LOW | T1D |
| 3 | cohort | not serious | not serious | not serious | serious | strong association | 160 | 3-6 months vs  < 3-5 months | infancy | **RR 0.36** (0.17 to 0.75) | ⨁⨁⨁⨁ HIGH | T1D |
| **Question: Later introduction to meat compared to earlier introduction to meat for IA and T1D prevention** | | | | | | | | | | | | |
| 2 | cohort | not serious | not serious | not serious | very serious | none | 748 | ≥ 8-9 months vs  < 8 months | infancy | **RR 0.90** (0.71 to 1.14) | ⨁⨁◯◯ LOW | IA |
| 2 | cohort | not serious | not serious | not serious | very serious | none | 78 | 4-9 months vs  < 4-8 months | infancy | **RR 0.92** (0.28 to 2.97) | ⨁⨁◯◯ LOW | T1D |
| **Question: Later introduction to fish compared to earlier introduction to fish for IA prevention** | | | | | | | | | | | | |
| 2 | cohort | not serious | not serious | not serious | very serious | none | 718 | per 1 month delay | infancy | **RR 1.00** (0.95 to 1.05) | ⨁⨁◯◯ LOW | IA |
| **Question: Later introduction to fruit compared to earlier introduction to fruit for IA and T1D prevention** | | | | | | | | | | | | |
| 3 | cohort | not serious | not serious | not serious | very serious | none | 857 | ≥ 4-6 months vs  < 4-5 months | infancy | **RR 0.81** (0.53 to 1.24) | ⨁⨁◯◯ LOW | IA |
| 2 | cohort | not serious | not serious | not serious | very serious | none | 78 | ≥ 6 months vs  4-6 months | infancy | **RR 1.16** (0.64 to 2.12) | ⨁⨁◯◯ LOW | T1D |
| 2 | cohort | not serious | not serious | not serious | very serious | strong association | 78 | 4-6 months vs  < 4-5 months | infancy | **RR 0.47** (0.25 to 0.86) | ⨁⨁⨁◯ MODERATE | T1D |
| **Question: Later introduction to vegetables compared to earlier introduction to vegetables for IA and T1D prevention** | | | | | | | | | | | | |
| 3 | cohort | not serious | not serious | not serious | serious | none | 994 | per 1 month delay | infancy | **RR 0.99** (0.91 to 1.07) | ⨁⨁⨁◯ MODERATE | IA |
| 2 | cohort | not serious | not serious | not serious | very serious | none | 78 | ≥ 6 months vs  4-6 months | infancy | **RR 1.18** (0.66 to 2.10) | ⨁⨁◯◯ LOW | T1D |
| 2 | cohort | not serious | not serious | not serious | very serious | none | 78 | 4-6 months vs  < 4-5 months | infancy | **RR 0.73** (0.33 to 1.63) | ⨁⨁◯◯ LOW | T1D |
| **Question: Higher intake of cow’s milk products compared to lower intake of cow’s milk products for IA and T1D prevention** | | | | | | | | | | | | |
| 3 | cohort | not serious | not serious | not serious | serious | none | 424 | per 2-3 portions/day | childhood | **RR 1.25** (1.06 to 1.47) | ⨁⨁⨁◯ MODERATE | IA |
| 3 | cohort | not serious | not serious | not serious | serious | none | 458 | per 2-3 portions/day | fetal | **RR 1.02** (0.90 to 1.16) | ⨁⨁⨁◯ MODERATE | IA |
| 3 | 2 case-control  1 nested case-control | serious | not serious | not serious | serious | none | 542 | ≥ 2-3 glasses of milk/day vs  < 2-3 glasses of milk/day | childhood | **RR 1.81** (1.12 to 2.91) | ⨁⨁◯◯ LOW | T1D |
| 2 | 1 cohort 1 case-control | serious | not serious | not serious | serious | none | 257 | cow’s milk protein per 10 g/day | childhood | **RR 1.35** (1.13 to 1.60) | ⨁⨁◯◯ LOW | T1D |
| **Question: Higher intake of meat compared to lower intake of meat for IA and T1D prevention** | | | | | | | | | | | | |
| 3 | cohort | not serious | not serious | not serious | very serious | none | 458 | per 100 g/day | fetal | **RR 1.15** (0.90 to 1.47) | ⨁⨁◯◯ LOW | IA |
| 2 | 1 cohort 1 case-control | serious | not serious | serious | very serious | none | 399 | high vs low/ continuous intake | childhood | **RR 1.78** (1.09 to 2.90) | ⨁◯◯◯ VERY LOW | T1D |
| **Question: Higher intake of fish compared to lower intake of fish for IA and T1D prevention** | | | | | | | | | | | | |
| 3 | cohort | not serious | not serious | not serious | serious | none | 458 | per 100 g/day | fetal | **RR 0.57** (0.32 to 1.04) | ⨁⨁⨁◯ MODERATE | IA |
| 2 | 1 cohort 1 case-control | serious | not serious | serious | very serious | none | 399 | high vs low/ continuous intake | childhood | **RR 3.29** (0.94 to 11.53) | ⨁◯◯◯ VERY LOW | T1D |
| **Question: Higher intake of egg compared to lower intake of egg for T1D prevention** | | | | | | | | | | | | |
| 2 | 2 cohort | serious | not serious | not serious | very serious | none | 252 | per 50 g/day | fetal | **RR 0.80** (0.43 to 1.50) | ⨁◯◯◯ VERY LOW | IA |
| 2 | 1 cohort 1 case-control | not serious | not serious | not serious | very serious | none | 252 | per 50 g/day | childhood | **RR 1.35** (0.19 to 9.59) | ⨁⨁◯◯ LOW | T1D |
| **Question: Higher intake of fruit juice compared to lower intake of fruit juice for T1D prevention** | | | | | | | | | | | | |
| 2 | 1 cohort 1 case-control | serious | serious | not serious | very serious | none | 99 | per 250 g/day | childhood | **RR 0.62** (0.08 to 4.79) | ⨁◯◯◯ VERY LOW | T1D |
| **Question: Higher intake of sugar sweetened beverages compared to lower intake of sugar sweetened beverages for T1D prevention** | | | | | | | | | | | | |
| 3 | 1 cohort 2 case-control | serious | not serious | serious | serious | none | 198 | high vs low/ continuous intake | childhood | **RR 1.14** (1.02 to 1.26) | ⨁◯◯◯ VERY LOW | T1D |
| **Question: Any quantity of maternal coffee intake compared to no coffee intake for T1D prevention in the offspring** | | | | | | | | | | | | |
| 2 | cohort | serious | very serious | not serious | very serious | none | 329 | high vs low | fetal | **RR 0.94** (0.38 to 2.32) | ⨁◯◯◯ VERY LOW | IA |
| 2 | case-control | serious | very serious | not serious | very serious | none | 865 | any vs none | fetal | **RR 1.51** (0.50 to 4.57) | ⨁◯◯◯ VERY LOW | T1D |
| **Question: Higher maternal fruit intake compared to lower fruit intake for IA prevention in the offspring** | | | | | | | | | | | | |
| 2 | cohort | serious | not serious | not serious | very serious | none | 165 | per 100 g/day | fetal | **RR 0.96** (0.71 to 1.30) | ⨁◯◯◯ VERY LOW | IA |
| **Question: Higher maternal vegetable intake compared to lower vegetable intake for IA prevention in the offspring** | | | | | | | | | | | | |
| 3 | cohort | not serious | not serious | not serious | very serious | none | 356 | per 100 g/day | fetal | **RR 0.60** (0.34 to 1.07) | ⨁⨁◯◯ LOW | IA |
| **Question: Higher maternal root vegetable intake compared to lower root vegetable intake for IA prevention in the offspring** | | | | | | | | | | | | |
| 3 | cohort | not serious | not serious | not serious | very serious | none | 356 | per 100 g/day | fetal | **RR 1.03** (0.65 to 1.62) | ⨁⨁◯◯ LOW | IA |
| **Question: Higher intake of protein compared to lower intake of protein for T1D prevention** | | | | | | | | | | | | |
| 2 | case-control | serious | not serious | very serious  (Swedish population) | very serious | strong association | 438 | high vs low | childhood | **RR 2.52** (1.56 to 4.08) | ⨁◯◯◯ VERY LOW | T1D |
| **Question: Higher intake of gluten compared to lower intake of gluten for IA and T1D prevention** | | | | | | | | | | | | |
| 2 | cohort | not serious | very serious | not serious | very serious | none | 424 | per 10 g/day | childhood | **RR 1.79** (0.55 to 5.85) | ⨁◯◯◯ VERY LOW | IA |
| 3 | cohort | not serious | serious | not serious | very serious | none | 403 | per 10 g/day | childhood | **RR 1.63** (0.58 to 4.62) | ⨁◯◯◯ VERY LOW | T1D |
| 2 | cohort | not serious | not serious | not serious | very serious | none | 593 | per 10 g/day | fetal | **RR 1.18** (0.92 to 1.51) | ⨁⨁◯◯ LOW | T1D |
| **Question: Higher intake of carbohydrates compared to lower intake of carbohydrates for T1D prevention** | | | | | | | | | | | | |
| 4 | 1 cohort 3 case-control | serious | not serious | serious | not serious | none | 535 | high vs low/ continuous intake | childhood | **RR 1.94** (1.35 to 2.81) | ⨁⨁◯◯ LOW | T1D |
| **Question: Higher intake of sugar compared to lower intake of sugar for T1D prevention** | | | | | | | | | | | | |
| 3 | 1 cohort 2 case-control | serious | not serious | serious | serious | none | 196 | high vs low/ continuous intake | childhood | **RR 1.80** (1.24 to 2.62) | ⨁◯◯◯ VERY LOW | T1D |
| **Question: Higher intake of fiber compared to lower intake of fiber for IA and T1D prevention** | | | | | | | | | | | | |
| 3 | cohort | not serious | serious | not serious | very serious | none | 532 | per 10 g/day | childhood | **RR 1.14** (0.66 to 1.97) | ⨁◯◯◯ VERY LOW | IA |
| 4 | 3 cohort 1 case-control | not serious | serious | not serious | very serious | none | 232 | per 10 g/day | childhood | **RR 1.50** (0.58 to 3.87) | ⨁◯◯◯ VERY LOW | T1D |
| **Question: Higher intake of omega-3 fatty acids compared to lower intake of omega-3 fatty acids for IA and T1D prevention** | | | | | | | | | | | | |
| 2 | cohort | serious | serious | not serious | very serious | none | 230 | per 1 SD increase | childhood/infancy | **RR 0.75** (0.33 to 1.66) | ⨁◯◯◯ VERY LOW | IA |
| 2 | cohort | not serious | serious | not serious | very serious | none | 987 | per 1 SD increase | fetal | **RR 1.05** (0.87 to 1.27) | ⨁◯◯◯ VERY LOW | IA |
| 3 | 2 cohort 1 case-control | serious | very serious | serious | very serious | none | 641 | supplementation per 1 time/week/ per 1 SD increase | childhood/infancy | **RR 0.69** (0.30 to 1.62) | ⨁◯◯◯ VERY LOW | T1D |
| 2 | 1 cohort 1 case-control | serious | not serious | serious | serious | none | 589 | supplementation per 1 time/week / per 1 SD increase | fetal | **RR 1.00** (0.95 to 1.05) | ⨁◯◯◯ VERY LOW | T1D |
| **Question: Higher intake of omega-6 fatty acids compared to lower intake of omega-6 fatty acids for IA and T1D prevention** | | | | | | | | | | | | |
| 2 | cohort | serious | not serious | not serious | very serious | none | 230 | per 1 SD increase | childhood/infancy | **RR 1.18** (0.91 to 1.53) | ⨁◯◯◯ VERY LOW | IA |
| 2 | cohort | serious | not serious | serious | very serious | none | 126 | per 1 SD increase | childhood/infancy | **RR 1.08** (0.89 to 1.31) | ⨁◯◯◯ VERY LOW | T1D |
| **Question: Vitamin D supplementation compared to no vitamin D supplementation for IA and T1D prevention** | | | | | | | | | | | | |
| 2 | cohort | not serious | very serious | not serious | very serious | none | 1521 | any vs none | fetal | **RR 0.90** (0.58 to 1.40) | ⨁◯◯◯ VERY LOW | IA |
| 7 | 1 cohort 6 case-control | serious | very serious | not serious | not serious | strong association | 2248 | any vs none | infancy | **RR 0.38** (0.20 to 0.74) | ⨁⨁◯◯ LOW | T1D |
| 2 | 1 cohort 1 case-control | serious | not serious | not serious | very serious | none | 524 | any vs none | fetal | **RR 1.00** (0.76 to 1.32) | ⨁◯◯◯ VERY LOW | T1D |
| **Question: Higher intake of vitamin A compared to lower intake of vitamin A for T1D prevention** | | | | | | | | | | | | |
| 2 | case-control | serious | not serious | serious | very serious | none | 570 | high vs low/ supplementation | childhood/infancy | **RR 0.73** (0.55 to 0.96) | ⨁◯◯◯ VERY LOW | T1D |
| **Question: Higher intake of vitamin C compared to lower intake of vitamin C for T1D prevention** | | | | | | | | | | | | |
| 2 | case-control | serious | not serious | serious | very serious | strong association | 249 | high vs low/ supplementation | childhood | **RR 0.47** (0.32 to 0.71) | ⨁◯◯◯ VERY LOW | T1D |
| **Question: Higher intake of iron compared to lower intake of iron for T1D prevention** | | | | | | | | | | | | |
| 3 | 1 case-cohort  2 case-control | serious | very serious | serious | very serious | none | 374 | per 10 mg/day/ supplementation yes vs no | childhood/infancy | **RR 1.35** (0.48 to 3.81) | ⨁◯◯◯ VERY LOW | T1D |
| 2 | 1 cohort 1 case-cohort | not serious | not serious | not serious | very serious | none | 611 | iron supplementation yes vs no | fetal | **RR 1.21** (0.97 to 1.52) | ⨁⨁◯◯ LOW | T1D |
| **Question: Higher intake of nitrate compared to lower intake of nitrate for T1D prevention** | | | | | | | | | | | | |
| 2 | case-control | not serious | not serious | serious | very serious | none | 650 | high vs low | childhood | **RR 1.01** (0.64 to 1.59) | ⨁◯◯◯ VERY LOW | T1D |
| **Question: Higher intake of nitrite compared to lower intake of nitrite for T1D prevention** | | | | | | | | | | | | |
| 2 | case-control | not serious | not serious | serious | very serious | strong association | 650 | high vs low | childhood | **RR 2.26** (1.63 to 3.12) | ⨁⨁◯◯ LOW | T1D |
| **Question: Higher intake of nitrosamines compared to lower intake of nitrosamines for T1D prevention** | | | | | | | | | | | | |
| 3 | case-control | serious | not serious | serious | very serious | none | 611 | high vs low | childhood | **RR 1.22** (0.85 to 1.73) | ⨁◯◯◯ VERY LOW | T1D |
| **Question: Higher levels of 25(OH)D compared to lower levels of 25(OH)D in blood for IA and T1D prevention** | | | | | | | | | | | | |
| 3 | 1 case-cohort 2 nested case-control | not serious | serious | not serious | serious | none | 634 | per 10 nmol/L | infancy | **RR 0.94** (0.87 to 1.01) | ⨁⨁◯◯ LOW | IA |
| 4 | 1 case-cohort  1 nested case-control 2 case-control | not serious | not serious | not serious | serious | none | 1099 | per 10 nmol/L | at birth | **RR 0.97** (0.90 to 1.04) | ⨁⨁⨁◯ MODERATE | T1D |
| 3 | 2 cohort 1 nested case-control | not serious | not serious | not serious | serious | none | 252 | per 10 nmol/L | childhood | **RR 0.93** (0.85 to 1.02) | ⨁⨁⨁◯ MODERATE | T1D |
| 3 | 1 cohort  2 nested case-control | serious | not serious | not serious | serious | none | 1315 | per 10 nmol/L | adulthood | **RR 0.92** (0.89 to 0.95) | ⨁⨁◯◯ LOW | T1D |
| 2 | 1 case-cohort 1 nested case-control | not serious | not serious | not serious | serious | none | 539 | per 10 nmol/L | fetal | **RR 1.00** (0.96 to 1.05) | ⨁⨁⨁◯ MODERATE | T1D |
| **Question: Higher levels of omega-3 fatty acids compared to lower levels of omega-3 fatty acids in blood for and IA prevention** | | | | | | | | | | | | |
| 2 | 1 cohort 1 nested case-control | not serious | very serious | serious | very serious | none | 280 | continuous increase | childhood/infancy | **RR 0.80** (0.57 to 1.14) | ⨁◯◯◯ VERY LOW | IA |
| **Question: Higher levels of omega-6 fatty acids compared to lower levels of omega-6 fatty acids in blood for and IA prevention** | | | | | | | | | | | | |
| 2 | 1 cohort 1 nested case-control | not serious | serious | serious | very serious | none | 280 | continuous increase | childhood/infancy | **RR 0.94** (0.74 to 1.19) | ⨁◯◯◯ VERY LOW | IA |

IA, islet autoimmunity; T1D, type 1 diabetes; CI, confidence interval; SD, standard deviation

**Supplementary Figure 1. Any breastfeeding and IA (long vs short)**

Summary of relative risk of islet autoimmunity for long vs short any breastfeeding. 95% CI, 95% confidence interval

**Supplementary Figure 2. Any breastfeeding and IA (per additional month)**

Summary of relative risk of islet autoimmunity per additional month of any breastfeeding. 95% CI, 95% confidence interval

**Supplementary Figure 3. Any breastfeeding and T1D (long vs short)**

Summary of relative risk of type 1 diabetes for long versus short any breastfeeding. 95% CI, 95% confidence interval

**Supplementary Figure 4. Any breastfeeding and T1D (per additional month)**

Summary of relative risk of type 1 diabetes per additional month of any breastfeeding. 95% CI, 95% confidence interval; MoBa, Mother and Child Cohort Study; DNBC, Danish National Birth Cohort

**Supplementary Figure 5. Exclusive breastfeeding and IA (long vs short)**

Summary of relative risk of islet autoimmunity for long versus short exclusive breastfeeding. 95% CI, 95% confidence interval

**Supplementary Figure 6. Exclusive breastfeeding and IA (per additional month)**

Summary of relative risk of islet autoimmunity per additional month of exclusive breastfeeding. 95% CI, 95% confidence interval

**Supplementary Figure 7. Exclusive breastfeeding and T1D (long vs short)**

Summary of relative risk of type 1 diabetes for long versus short exclusive breastfeeding. 95% CI, 95% confidence interval

**Supplementary Figure 8. Exclusive breastfeeding and T1D (per additional month)**

Summary of relative risk of type 1 diabetes per additional month of exclusive breastfeeding. 95% CI, 95% confidence interval; MoBa, Mother and Child Cohort Study; DNBC, Danish National Birth Cohort

**Supplementary Figure 9. Any breastfeeding and T1D (yes vs no)**

Summary of relative risk of type 1 diabetes for any versus no breastfeeding. 95% CI, 95% confidence interval

**Supplementary Figure 10. Exclusive breastfeeding and T1D (yes vs no)**

Summary of relative risk of type 1 diabetes for exclusive versus no exclusive breastfeeding. 95% CI, 95% confidence interval

**Supplementary Figure 11. Cow's milk introduction and IA.**

Summary of relative risk of islet autoimmunity for late versus early introduction to cow’s milk. 95% CI, 95% confidence interval

**Supplementary Figure 12. Cow's milk introduction and T1D**

Summary of relative risk of type 1 diabetes for late versus early introduction to cow’s milk. 95% CI, 95% confidence interval

**Supplementary Figure 13. Infant formula introduction and IA**

Summary of relative risk of islet autoimmunity per one month delay of introduction to infant formula. 95% CI, 95% confidence interval

**Supplementary Figure 14. Infant formula introduction and T1D (late vs early)**

Summary of relative risk of type 1 diabetes for late versus early introduction to infant formula. 95% CI, 95% confidence interval

**Supplementary Figure 15. Infant formula introduction and T1D (per 1 month delay)**

Summary of relative risk of type 1 diabetes per one month delay of introduction to infant formula. 95% CI, 95% confidence interval; MoBa, Mother and Child Cohort Study; DNBC, Danish National Birth Cohort

**Supplementary Figure 16. Solid food introduction and IA**

Summary of relative risk of islet autoimmunity per one month delay of introduction to solid food. 95% CI, 95% confidence interval

**Supplementary Figure 17. Solid food introduction and T1D**

Summary of relative risk of type 1 diabetes for late versus early introduction to solid food. 95% CI, 95% confidence interval; MoBa, Mother and Child Cohort Study; DNBC, Danish National Birth Cohort

**Supplementary Figure 18. Cereal introduction and IA**

Summary of relative risk of islet autoimmunity for late versus early introduction to cereal. 95% CI, 95% confidence interval

**Supplementary Figure 19. Cereal introduction and T1D**

Summary of relative risk of type 1 diabetes for late versus early introduction to cereal. 95% CI, 95% confidence interval

**Supplementary Figure 20. Gluten introduction and IA**

Summary of relative risk of islet autoimmunity for late versus early introduction to gluten. 95% CI, 95% confidence interval

**Supplementary Figure 21. Gluten introduction and T1D**

Summary of relative risk of type 1 diabetes for late versus early introduction to gluten. 95% CI, 95% confidence interval

**Supplementary** **Figure 22**. **Meat introduction and IA**

Summary of relative risk of islet autoimmunity for late versus early introduction to meat. 95% CI, 95% confidence interval

**Supplementary Figure 23. Meat introduction and T1D**

Summary of relative risk of type 1 diabetes for late versus early introduction to meat. 95% CI, 95% confidence interval

**Supplementary Figure 24. Fish introduction and IA**

Summary of relative risk of islet autoimmunity per one month delay of introduction to fish. 95% CI, 95% confidence interval

**Supplementary Figure 25. Fruit introduction and IA**

Summary of relative risk of islet autoimmunity for late versus early introduction to fruit. 95% CI, 95% confidence interval

**Supplementary Figure 26. Fruit introduction and T1D**

Summary of relative risk of type 1 diabetes for late versus early introduction to fruit. 95% CI, 95% confidence interval

**Supplementary Figure 27. Vegetable introduction and IA**

Summary of relative risk of islet autoimmunity per one month delay of introduction to vegetables. 95% CI, 95% confidence interval

**Supplementary Figure 28. Vegetable introduction and T1D**

Summary of relative risk of type 1 diabetes for late versus early introduction to vegetables. 95% CI, 95% confidence interval

**Supplementary Figure 29. Cow's milk intake and IA**

Summary of relative risk of islet autoimmunity per 2-3 portions/day increase of childhood and maternal cow’s milk products intake. 95% CI, 95% confidence interval

**Supplementary Figure 30. Cow's milk intake and T1D**

Summary of relative risk of type 1 diabetes for high versus low cow’s milk intake and per 10 g/day increase of cow’s milk protein intake in childhood. 95% CI, 95% confidence interval

**Supplementary Figure 31. Maternal meat intake and IA**

Summary of relative risk of islet autoimmunity per 100 g/day increase of maternal meat intake. 95% CI, 95% confidence interval

**Supplementary Figure 32. Meat intake and T1D**

Summary of relative risk of type 1 diabetes for high versus low/continuous increase of meat intake in chidhood. 95% CI, 95% confidence interval

**Supplementary Figure 33. Maternal fish intake and IA**

Summary of relative risk of islet autoimmunity per 100 g/day increase of maternal fish intake. 95% CI, 95% confidence interval

**Supplementary Figure 34**. **Fish intake and T1D**

Summary of relative risk of islet autoimmunity for high versus low/continuous increase of fish intake in childhood. 95% CI, 95% confidence interval; SD, standard deviation

**Supplementary Figure 35. Egg intake and IA**

Summary of relative risk of islet autoimmunity per 50 g/day increase of childhood and maternal egg intake. 95% CI, 95% confidence interval

**Supplementary Figure 36. Egg intake and T1D**

Summary of relative risk of type 1 diabetes per 50 g/day increase of egg intake in childhood. 95% CI, 95% confidence interval

**Supplementary Figure 37. Fruit juice intake and T1D**

Summary of relative risk of type 1 diabetes per 250 g/day increase of fruit juice intake in childhood. 95% CI, 95% confidence interval

**Supplementary Figure 38. Sugar sweetened beverage intake and T1D**

Summary of relative risk of type 1 diabetes for high versus low/continuous increase of sugar sweetened beverage intake in childhood. 95% CI, 95% confidence interval

**Supplementary Figure 39. Maternal coffee intake and IA**

Summary of relative risk of islet autoimmunity for high versus low maternal coffee intake. 95% CI, 95% confidence interval

**Supplementary Figure 40. Coffee intake and T1D**

Summary of relative risk of type 1 diabetes for any versus none childhood and maternal coffee intake. 95% CI, 95% confidence interval

**Supplementary Figure 41. Maternal fruit intake and IA**

Summary of relative risk of islet autoimmunity per 100 g/day increase of maternal fruit intake. 95% CI, 95% confidence interval

**Supplementary Figure 42. Maternal vegetable intake and IA**

Summary of relative risk of islet autoimmunity per 100 g/day increase of maternal vegetable intake. 95% CI, 95% confidence interval

**Supplementary Figure 43. Maternal root vegetable intake and IA**

Summary of relative risk of islet autoimmunity per 100 g/day increase of maternal root vegetable intake. 95% CI, 95% confidence interval

**Supplementary Figure 44. Protein intake and T1D**

Summary of relative risk of type 1 diabetes for high versus low protein intake in childhood. 95% CI, 95% confidence interval

**Supplementary Figure 45. Gluten intake and IA**

Summary of relative risk of islet autoimmunity per 10 g/day increase of childhood and maternal gluten intake. 95% CI, 95% confidence interval

**Supplementary Figure 46. Gluten intake and T1D**

Summary of relative risk of type 1 diabetes per 10 g/day increase of childhood and maternal gluten intake. 95% CI, 95% confidence interval

**Supplementary Figure 47. Carbohydrate intake and T1D**

Summary of relative risk of type 1 diabetes for high versus low/continuous increase of carbohydrate intake in childhood. 95% CI, 95% confidence interval

**Supplementary Figure 48. Sugar intake and T1D**

Summary of relative risk of type 1 diabetes for high versus low/continuous increase of sugar intake in childhood. 95% CI, 95% confidence interval

**Supplementary Figure 49. Fiber intake and IA**

Summary of relative risk of islet autoimmunity per 10 g/day increase of fiber intake in childhood. 95% CI, 95% confidence interval

**Supplementary Figure 50. Fiber intake and T1D**

Summary of relative risk of type 1 diabetes per 10 g/day increase of childhood and maternal fiber intake. 95% CI, 95% confidence interval

**Supplementary Figure 51. Omega-3 intake and IA**

Summary of relative risk of islet autoimmunity per one standard deviation increase of infancy or childhood and maternal omega-3 intake. 95% CI, 95% confidence interval; SD, standard deviation

**Supplementary Figure 52. Omega-3 intake and T1D**

Summary of relative risk of type 1 diabetes for continuous increase of infancy or childhood and maternal omega-3 intake. 95% CI, 95% confidence interval; SD, standard deviation

**Supplementary Figure 53. Omega-6 intake and IA**

Summary of relative risk of islet autoimmunity per one standard deviation increase of infancy or childhood and maternal omega-6 intake. 95% CI, 95% confidence interval; SD, standard deviation

**Supplementary Figure 54. Omega-6 intake and T1D**

Summary of relative risk of type 1 diabetes per one standard deviation increase of infancy or childhood and maternal omega-6 intake. 95% CI, 95% confidence interval; SD, standard deviation

**Supplementary Figure 55. Vitamin D supplementation and IA**

Summary of relative risk of islet autoimmunity for maternal vitamin D supplementation (yes versus no). 95% CI, 95% confidence interval

**Supplementary Figure 56. Vitamin D supplementation and T1D**

Summary of relative risk of type 1 diabetes for infancy and maternal vitamin D supplementation (yes versus no). 95% CI, 95% confidence interval

**Supplementary Figure 57. Vitamin A intake and T1D**

Summary of relative risk of type 1 diabetes for high versus low vitamin A intake in infancy or childhood. 95% CI, 95% confidence interval

**Supplementary Figure 58. Vitamin C intake and T1D**

Summary of relative risk of type 1 diabetes for high versus low vitamin C intake in childhood. 95% CI, 95% confidence interval

**Supplementary Figure 59. Iron intake and T1D**

Summary of relative risk of type 1 diabetes per 10 mg/day increase of iron intake or for supplementation (yes versus no) in infancy or childhood and for maternal iron supplementation (yes versus no). 95% CI, 95% confidence interval

**Supplementary Figure 60. Nitrate intake and T1D**

Summary of relative risk of type 1 diabetes for high versus low nitrate intake in childhood and per 10 mg/day increase of maternal nitrate intake. 95% CI, 95% confidence interval

**Supplementary Figure 61. Nitrite intake and T1D**

Summary of relative risk of type 1 diabetes for high versus low nitrite intake in childhood and per 5 mg/day increase of maternal nitrite intake. 95% CI, 95% confidence interval

**Supplementary Figure 62. Nitrosamine intake and T1D**

Summary of relative risk of type 1 diabetes for high versus low childhood and maternal nitrosamine intake. 95% CI, 95% confidence interval

**Supplementary Figure 63. Vitamin D levels in blood and IA**

Summary of relative risk of islet autoimmunity per 10 nmol/L increase of vitamin D levels in blood in infancy. 95% CI, 95% confidence interval

**Supplementary Figure 64. Vitamin D levels in blood and T1D**

Summary of relative risk of type 1 per 10 nmol/L increase of infancy, childhood, adulthood, and maternal vitamin D levels in blood. 95% CI, 95% confidence interval

**Supplementary Figure 65. Vitamin D levels in blood and progression from IA to T1D**

Summary of relative risk of progression to type 1 diabetes per 10 nmol/L increase of plasma vitamin D levels in childhood. 95% CI, 95% confidence interval

**Supplementary Figure 66. Omega-3 levels in blood and IA**

Summary of relative risk of islet autoimmunity for continuous increase of omega-3 levels in blood in infancy or childhood. 95% CI, 95% confidence interval

**Supplementary Figure 67. Omega-6 levels in blood and IA**

Summary of relative risk of islet autoimmunity for continuous increase of omega-6 levels in blood in infancy or childhood. 95% CI, 95% confidence interval

**Supplementary Figure 68. Contour-enhanced funnel plot of included studies on any breastfeeding (long vs short) and risk of T1D.**

Egger’s test for asymmetry: p = 0.1448

**Supplementary Figure 69. Contour-enhanced funnel plot of included studies on any breastfeeding (yes vs no) and risk of T1D.**

Egger’s test for asymmetry: p = 0.0059

**Supplementary Figure 70. Contour-enhanced funnel plot of included studies on cow’s milk introduction and risk of T1D.**

Egger’s test for asymmetry: p = 0.7126

**Supplementary Figure 71. Subgroup analysis for long vs short any breastfeeding and T1D based on risk of bias and study design**

**Supplementary Figure 72. Subgroup analysis for any breastfeeding per additional month and T1D based on risk of bias and study design**

**Supplementary Figure 73. Subgroup analysis for any breastfeeding yes vs no and T1D based on risk of bias and study design**

**Supplementary Figure 74. Subgroup analysis for late vs early introduction to solid food and T1D based on risk of bias and study design**

MoBa, Mother and Child Cohort Study; DNBC, Danish National Birth Cohort

**Supplementary Figure 75. Subgroup analysis for late vs early introduction to solid food and T1D based on genetic risk**

**Supplementary Figure 76. Subgroup analysis for late vs early introduction to gluten and IA based on risk of bias**

**Supplementary Figure 77. Subgroup analysis for late vs early introduction to gluten and IA based on genetic risk**

**Supplementary Figure 78. Subgroup analysis for vitamin D supplementation and T1D based on risk of bias and study design**

**Supplementary Figure 79.** **Sensitivity analysis based on prospective evidence**

Summary relative risks and 95% confidence intervals of type 1 diabetes in relation to diet, based on prospective studies; SD, standard deviation

# **List of Supplementary references**

1. Ahadi M, Tabatabaeiyan M, Moazzami K. Association between environmental factors and risk of type 1 diabetes - a case-control study. *Endokrynol Pol*. 2011;**62**(2):134–7.

2. Antvorskov JC, Halldorsson TI, Josefsen K, Svensson J, Granström C, Roep BO, et al. Association between maternal gluten intake and type 1 diabetes in offspring: National prospective cohort study in Denmark. *BMJ*. 2018;**362**:1–9.

3. Ashraf AP, Eason NB, Kabagambe EK, Haritha J, Meleth S, McCormick KL. Dietary iron intake in the first 4 months of infancy and the development of type 1 diabetes: a pilot study. *Diabetol Metab Syndr*. 2010 Dec 20;**2**(1):58.

4. Awadalla N, Hegazy A, Abd El-Salam M, Elhady M. Environmental Factors Associated with Type 1 Diabetes Development: A Case Control Study in Egypt. *Int J Environ Res Public Health*. 2017 Jun 7;**14**(6):615.

5. Bener A, Alsaied A, Al-Ali M, Al-Kubaisi A, Basha B, Abraham A, et al. High prevalence of vitamin D deficiency in type 1 diabetes mellitus and healthy children. *Acta Diabetol*. 2009 Sep 10;**46**(3):183–9.

6. Benson VS, Vanleeuwen JA, Taylor J, McKinney PA, Van Til L. Food consumption and the risk of type 1 diabetes in children and youth: a population-based, case-control study in Prince Edward Island, Canada. *J Am Coll Nutr*. 2008;**27**(3):414–20.

7. Benson VS, Vanleeuwen JA, Taylor J, Somers GS, McKinney PA, Van Til L. Type 1 diabetes mellitus and components in drinking water and diet: a population-based, case-control study in Prince Edward Island, Canada. *J Am Coll Nutr*. 2010;**29**(6):612–24.

8. Beyerlein A, Liu X, Uusitalo UM, Harsunen M, Norris JM, Foterek K, et al. Dietary intake of soluble fiber and risk of islet autoimmunity by 5 y of age: results from the TEDDY study. *Am J Clin Nutr*. 2015 Aug 1;**102**(2):345–52.

9. Bodington MJ, McNally PG, Burden AC. Cow’s milk and type 1 childhood diabetes: no increase in risk. *Diabet Med*. 1994;**11**(7):663–5.

10. Brekke HK, Ludvigsson J. Vitamin D supplementation and diabetes-related autoimmunity in the ABIS study. *Pediatr Diabetes*. 2007;**8**(1):11–4.

11. Brekke HK, Ludvigsson J. Daily vegetable intake during pregnancy negatively associated to islet autoimmunity in the offspring-The ABIS study. *Pediatr Diabetes*. 2010 Sep 16;**11**(4):244–50.

12. Cadario F, Savastio S, Pagliardini V, Bagnati M, Vidali M, Cerutti F, et al. Vitamin D levels at birth and risk of type 1 diabetes in childhood: a case–control study. *Acta Diabetol*. 2015 Dec 28;**52**(6):1077–81.

13. Chmiel R, Beyerlein A, Knopff A, Hummel S, Ziegler A-G, Winkler C. Early infant feeding and risk of developing islet autoimmunity and type 1 diabetes. *Acta Diabetol*. 2015 Jun 20;**52**(3):621–4.

14. Dahlquist G, Blom L, Lonnberg G. The Swedish Childhood Diabetes Study--a multivariate analysis of risk determinants for diabetes in different age groups. *Diabetologia*. 1991;**34**(10):757–62.

15. Dahlquist G, Savilahti E, Landin-Olsson M. An increased level of antibodies to beta-lactoglobulin is a risk determinant for early-onset type 1 (insulin-dependent) diabetes mellitus independent of islet cell antibodies and early introduction of cow’s milk. *Diabetologia*. 1992;**35**(10):980–4.

16. The EURODIAB Substudy 2 Study Group. Vitamin D supplement in early childhood and risk for Type I (insulin- dependent) diabetes mellitus. *Diabetologia*. 1999;**42**(1):51–4.

17. Frederiksen B, Kroehl M, Lamb MM, Seifert J, Barriga K, Eisenbarth GS, et al. Infant Exposures and Development of Type 1 Diabetes Mellitus. *JAMA Pediatr*. 2013 Sep 1;**167**(9):808.

18. Gimeno SG, de Souza JM. IDDM and milk consumption. A case-control study in Sao Paulo, Brazil. *Diabetes Care*. 1997;**20**(8):1256–60.

19. Glatthaar C, Whittall DE, Welborn TA, Gibson MJ, Brooks BH, Ryan MM, et al. Diabetes in Western Australian children: descriptive epidemiology. *Med J Aust*. 1988;**148**(3):117–23.

20. Gorham ED, Garland CF, Burgi AA, Mohr SB, Zeng K, Hofflich H, et al. Lower prediagnostic serum 25-hydroxyvitamin D concentration is associated with higher risk of insulin-requiring diabetes: a nested case–control study. *Diabetologia*. 2012 Dec 7;**55**(12):3224–7.

21. Granfors M, Augustin H, Ludvigsson J, Brekke HK. No association between use of multivitamin supplement containing vitamin D during pregnancy and risk of Type 1 Diabetes in the child. *Pediatr Diabetes*. 2016;**17**(7):525–30.

22. Hakola L, Miettinen ME, Syrjälä E, Åkerlund M, Takkinen H-M, Korhonen TE, et al. Association of Cereal, Gluten, and Dietary Fiber Intake With Islet Autoimmunity and Type 1 Diabetes. *JAMA Pediatr*. 2019 Oct 1;**173**(10):953.

23. Hall K, Frederiksen B, Rewers M, Norris JM. Daycare Attendance, Breastfeeding, and the Development of Type 1 Diabetes: The Diabetes Autoimmunity Study in the Young. *Biomed Res Int*. 2015;**2015**:1–5.

24. Hummel S, Beyerlein A, Tamura R, Uusitalo U, Andrén Aronsson C, Yang J, et al. First Infant Formula Type and Risk of Islet Autoimmunity in The Environmental Determinants of Diabetes in the Young (TEDDY) Study. *Diabetes Care*. 2017 Mar;**40**(3):398–404.

25. Hyppönen E, Kenward MG, Virtanen SM, Piitulainen A, Virta-Autio P, Tuomilehto J, et al. Infant feeding, early weight gain, and risk of type I diabetes. *Diabetes Care*. 1999;**22**(12):1961–5.

26. Hypponen E, Laara E, Reunanen A, Jarvelin MR, Virtanen SM. Intake of vitamin D and risk of type 1 diabetes: a birth-cohort study. *Lancet (London, England)*. 2001;**358**(9292):1500–3.

27. Jacobsen R, Thorsen SU, Cohen AS, Lundqvist M, Frederiksen P, Pipper CB, et al. Neonatal vitamin D status is not associated with later risk of type 1 diabetes: results from two large Danish population-based studies. *Diabetologia*. 2016 Sep 30;**59**(9):1871–81.

28. Jones ME, Swerdlow AJ, Gill LE, Goldacre MJ. Pre-natal and early life risk factors for childhood onset diabetes mellitus: a record linkage study. *Int J Epidemiol*. 1998;**27**(3):444–9.

29. Koivusaari K, Syrjälä E, Niinistö S, Takkinen H-M, Ahonen S, Åkerlund M, et al. Consumption of differently processed milk products in infancy and early childhood and the risk of islet autoimmunity. *Br J Nutr*. 2020 Jul 28;**124**(2):173–80.

30. Kostraba JN, Dorman JS, LaPorte RE, Scott FW, Steenkiste AR, Gloninger M, et al. Early infant diet and risk of IDDM in blacks and whites. A matched case-control study. *Diabetes Care*. 1992;**15**(5):626–31.

31. Kostraba JN, Cruickshanks KJ, Lawler-Heavner J, Jobim LF, Rewers MJ, Gay EC, et al. Early exposure to cow’s milk and solid foods in infancy, genetic predisposition, and risk of IDDM. *Diabetes*. 1993;**42**(2):288–95.

32. Kyvik KO, Green A, Svendsen A, Mortensen K. Breast feeding and the development of type 1 diabetes mellitus. *Diabet Med*. 1992;**9**(3):233–5.

33. Lamb MM, Myers MA, Barriga K, Zimmet PZ, Rewers M, Norris JM. Maternal diet during pregnancy and islet autoimmunity in offspring. *Pediatr Diabetes*. 2008 Apr;**9**(2):135–41.

34. Lamb MM, Yin X, Barriga K, Hoffman MR, Barón AE, Eisenbarth GS, et al. Dietary Glycemic Index, Development of Islet Autoimmunity, and Subsequent Progression to Type 1 Diabetes in Young Children. *J Clin Endocrinol Metab*. 2008 Oct;**93**(10):3936–42.

35. Lamb MM, Frederiksen B, Seifert JA, Kroehl M, Rewers M, Norris JM. Sugar intake is associated with progression from islet autoimmunity to type 1 diabetes: the Diabetes Autoimmunity Study in the Young. *Diabetologia*. 2015;**58**(9):2027–34.

36. Lamb MM, Miller M, Seifert JA, Frederiksen B, Kroehl M, Rewers M, et al. The effect of childhood cow’s milk intake and HLA-DR genotype on risk of islet autoimmunity and type 1 diabetes: The Diabetes Autoimmunity Study in the Young. *Pediatr Diabetes*. 2015 Feb;**16**(1):31–8.

37. Lund-Blix NA, Stene LC, Rasmussen T, Torjesen PA, Andersen LF, Rønningen KS. Infant Feeding in Relation to Islet Autoimmunity and Type 1 Diabetes in Genetically Susceptible Children: The MIDIA Study. *Diabetes Care*. 2015 Feb;**38**(2):257–63.

38. Lund-Blix NA, Dydensborg Sander S, Størdal K, Nybo Andersen A-M, Rønningen KS, Joner G, et al. Infant Feeding and Risk of Type 1 Diabetes in Two Large Scandinavian Birth Cohorts. *Diabetes Care*. 2017 Jul;**40**(7):920–7.

39. Lund-Blix NA, Dong F, Mårild K, Seifert J, Barón AE, Waugh KC, et al. Gluten Intake and Risk of Islet Autoimmunity and Progression to Type 1 Diabetes in Children at Increased Risk of the Disease: The Diabetes Autoimmunity Study in the Young (DAISY). *Diabetes Care*. 2019 May;**42**(5):789–96.

40. Lund-Blix NA, Tapia G, Mårild K, Brantsaeter AL, Njølstad PR, Joner G, et al. Maternal and child gluten intake and association with type 1 diabetes: The Norwegian Mother and Child Cohort Study. *PLoS Med*. 2020;**17**(3):e1003032.

41. Malcova H, Sumnik Z, Drevinek P, Venhacova J, Lebl J, Cinek O. Absence of breast-feeding is associated with the risk of type 1 diabetes: a case-control study in a population with rapidly increasing incidence. *Eur J Pediatr*. 2006;**165**(2):114–9.

42. Marshall AL, Chetwynd A, Morris JA, Placzek M, Smith C, Olabi A, et al. Type 1 diabetes mellitus in childhood: a matched case control study in Lancashire and Cumbria, UK. *Diabet Med*. 2004;**21**(9):1035–40.

43. Mattila M, Niinistö S, Takkinen H-M, Tapanainen H, Reinivuo H, Åkerlund M, et al. Maternal Nitrate and Nitrite Intakes during Pregnancy and Risk of Islet Autoimmunity and Type 1 Diabetes: The DIPP Cohort Study. *J Nutr*. 2020 Nov 19;**150**(11):2969–76.

44. Mayer EJ, Hamman RF, Gay EC, Lezotte DC, Savitz DA, Klingensmith GJ. Reduced risk of IDDM among breast-fed children. The Colorado IDDM Registry. *Diabetes*. 1988;**37**(12):1625–32.

45. McKinney PA, Parslow R, Gurney KA, Law GR, Bodansky HJ, Williams R. Perinatal and neonatal determinants of childhood type 1 diabetes. A case-control study in Yorkshire, U.K. *Diabetes Care*. 1999;**22**(6):928–32.

46. Meloni T, Marinaro AM, Mannazzu MC, Ogana A, La Vecchia C, Negri E, et al. IDDM and early infant feeding. Sardinian case-control study. *Diabetes Care*. 1997;**20**(3):340–2.

47. Miettinen ME, Niinistö S, Erlund I, Cuthbertson D, Nucci AM, Honkanen J, et al. Serum 25-hydroxyvitamin D concentration in childhood and risk of islet autoimmunity and type 1 diabetes: the TRIGR nested case–control ancillary study. *Diabetologia*. 2020 Apr 7;**63**(4):780–7.

48. Munger KL, Levin LI, Massa J, Horst R, Orban T, Ascherio A. Preclinical Serum 25-Hydroxyvitamin D Levels and Risk of Type 1 Diabetes in a Cohort of US Military Personnel. *Am J Epidemiol*. 2013 Mar 1;**177**(5):411–9.

49. Muntoni S, Mereu R, Atzori L, Mereu A, Galassi S, Corda S, et al. High meat consumption is associated with type 1 diabetes mellitus in a Sardinian case–control study. *Acta Diabetol*. 2013 Oct 4;**50**(5):713–9.

50. Niinistö S, Takkinen H-M, Uusitalo L, Rautanen J, Nevalainen J, Kenward MG, et al. Maternal dietary fatty acid intake during pregnancy and the risk of preclinical and clinical type 1 diabetes in the offspring. *Br J Nutr*. 2014 Mar 14;**111**(5):895–903.

51. Niinistö S, Takkinen H-M, Uusitalo L, Rautanen J, Vainio N, Ahonen S, et al. Maternal intake of fatty acids and their food sources during lactation and the risk of preclinical and clinical type 1 diabetes in the offspring. *Acta Diabetol*. 2015 Aug 7;**52**(4):763–72.

52. Niinistö S, Takkinen H-M, Erlund I, Ahonen S, Toppari J, Ilonen J, et al. Fatty acid status in infancy is associated with the risk of type 1 diabetes-associated autoimmunity. *Diabetologia*. 2017 Jul 4;**60**(7):1223–33.

53. Norris JM, Barriga K, Klingensmith G, Hoffman M, Eisenbarth GS, Erlich HA, et al. Timing of initial cereal exposure in infancy and risk of islet autoimmunity. *JAMA*. 2003;**290**(13):1713–20.

54. Norris JM, Yin X, Lamb MM, Barriga K, Seifert J, Hoffman M, et al. Omega-3 polyunsaturated fatty acid intake and islet autoimmunity in children at increased risk for type 1 diabetes. *JAMA*. 2007;**298**(12):1420–8.

55. Norris JM, Kroehl M, Fingerlin TE, Frederiksen BN, Seifert J, Wong R, et al. Erythrocyte membrane docosapentaenoic acid levels are associated with islet autoimmunity: the Diabetes Autoimmunity Study in the Young. *Diabetologia*. 2014 Feb 16;**57**(2):295–304.

56. Norris JM, Lee H-S, Frederiksen B, Erlund I, Uusitalo U, Yang J, et al. Plasma 25-Hydroxyvitamin D Concentration and Risk of Islet Autoimmunity. *Diabetes*. 2018 Jan;**67**(1):146–54.

57. Patterson CC, Carson DJ, Hadden DR, Waugh NR, Cole SK. A case-control investigation of perinatal risk factors for childhood IDDM in Northern Ireland and Scotland. *Diabetes Care*. 1994;**17**(5):376–81.

58. Pundziute-Lycka A, Persson L-A, Cedermark G, Jansson-Roth A, Nilsson U, Westin V, et al. Diet, growth, and the risk for type 1 diabetes in childhood: a matched case-referent study. *Diabetes Care*. 2014;**27**(12):2784–9.

59. Raab J, Giannopoulou EZ, Schneider S, Warncke K, Krasmann M, Winkler C, et al. Prevalence of vitamin D deficiency in pre-type 1 diabetes and its association with disease progression. *Diabetologia*. 2014 May 16;**57**(5):902–8.

60. Radon K, Windstetter D, Solfrank S, von Mutius E, Nowak D, Schwarz H-P, et al. Exposure to farming environments in early life and type 1 diabetes: a case-control study. *Diabetes*. 2005;**54**(11):3212–6.

61. Robertson L, Harrild K. Maternal and neonatal risk factors for childhood type 1 diabetes: a matched case-control study. *BMC Public Health*. 2010 Dec 27;**10**(1):281.

62. Rosenbauer J, Herzig P, Giani G. Early infant feeding and risk of type 1 diabetes mellitus-a nationwide population-based case-control study in pre-school children. *Diabetes Metab Res Rev*. 2008;**24**(3):211–22.

63. Sadauskaite-Kuehne V, Ludvigsson J, Padaiga Z, Jasinskiene E, Samuelsson U. Longer breastfeeding is an independent protective factor against development of type 1 diabetes mellitus in childhood. *Diabetes Metab Res Rev*. 2004;**20**(2):150–7.

64. Saukkonen T, Virtanen SM, Karppinen M, Reijonen H, Ilonen J, Rasanen L, et al. Significance of cow’s milk protein antibodies as risk factor for childhood IDDM: interactions with dietary cow’s milk intake and HLA-DQB1 genotype. Childhood Diabetes in Finland Study Group. *Diabetologia*. 1998;**41**(1):72–8.

65. Silvis K, Aronsson CA, Liu X, Uusitalo U, Yang J, Tamura R, et al. Maternal dietary supplement use and development of islet autoimmunity in the offspring: TEDDY study. *Pediatr Diabetes*. 2019 Dec 9;**20**(1):pedi.12794.

66. Simpson M, Brady H, Yin X, Seifert J, Barriga K, Hoffman M, et al. No association of vitamin D intake or 25-hydroxyvitamin D levels in childhood with risk of islet autoimmunity and type 1 diabetes: the Diabetes Autoimmunity Study in the Young (DAISY). *Diabetologia*. 2011 Nov 20;**54**(11):2779–88.

67. Sipetic S, Vlajinac H, Kocev N, Saji S. The Belgrade childhood diabetes study: prenatal and social associations for type 1 diabetes. *Paediatr Perinat Epidemiol*. 2004;**18**(1):33–9.

68. Sipetic SB, Vlajinac HD, Kocev NI, Marinkovic JM, Radmanovic SZ, Bjekic MD. The Belgrade childhood diabetes study: a multivariate analysis of risk determinants for diabetes. *Eur J Public Health*. 2005;**15**(2):117–22.

69. Sipetic S, Vlajinac H, Kocev N, Bjekic M, Sajic S. Early infant diet and risk of type 1 diabetes mellitus in Belgrade children. *Nutrition*. 2005;**21**(4):474–9.

70. Skaaby T, Husemoen LLN, Thuesen BH, Linneberg A. Prospective population-based study of the association between vitamin D status and incidence of autoimmune disease. *Endocrine*. 2015 Sep 11;**50**(1):231–8.

71. Skrodeniene E, Marčiulionyte D, Padaiga Z, Jašinskiene E, Sadauskaite-Kuehne V, Sanjeevi CB, et al. Associations between HLA class II haplotypes, environmental factors and type 1 diabetes mellitus in Lithuanian children with type 1 diabetes and controls. *Polish Ann Med*. 2010;**17**(1):7–15.

72. Stene LC, Joner G, Norwegian Childhood Diabetes Study G. Use of cod liver oil during the first year of life is associated with lower risk of childhood-onset type 1 diabetes: a large, population-based, case-control study. *Am J Clin Nutr*. 2003;**78**(6):1128–34.

73. Størdal K, McArdle HJ, Hayes H, Tapia G, Viken MK, Lund-Blix NA, et al. Prenatal iron exposure and childhood type 1 diabetes. *Sci Rep*. 2018 Dec 13;**8**(1):9067.

74. Svensson J, Carstensen B, Mortensen HB, Borch-Johnsen K. Early childhood risk factors associated with type 1 diabetes - Is gender important? *Eur J Epidemiol*. 2005;**20**(5):429–34.

75. Syrjälä E, Nevalainen J, Peltonen J, Takkinen H-M, Hakola L, Åkerlund M, et al. A Joint Modeling Approach for Childhood Meat, Fish and Egg Consumption and the Risk of Advanced Islet Autoimmunity. *Sci Rep*. 2019 Dec 23;**9**(1):7760.

76. Sørensen IM, Joner G, Jenum PA, Eskild A, Brunborg C, Torjesen PA, et al. Vitamin D-binding protein and 25-hydroxyvitamin D during pregnancy in mothers whose children later developed type 1 diabetes. *Diabetes Metab Res Rev*. 2016 Nov;**32**(8):883–90.

77. Tai TY, Wang CY, Lin LL, Lee LT, Tsai ST, Chen CJ. A case-control study on risk factors for Type 1 diabetes in Taipei City. *Diabetes Res Clin Pract*. 1998;**42**(3):197–203.

78. Tenconi MT, Devoti G, Comelli M, Pinon M, Capocchiano A, Calcaterra V, et al. Major childhood infectious diseases and other determinants associated with type 1 diabetes: a case-control study. *Acta Diabetol*. 2007;**44**(1):14–9.

79. Thorsen SU, Mårild K, Olsen SF, Holst KK, Tapia G, Granström C, et al. Lack of Association Between Maternal or Neonatal Vitamin D Status and Risk of Childhood Type 1 Diabetes: A Scandinavian Case-Cohort Study. *Am J Epidemiol*. 2018 Jun 1;**187**(6):1174–81.

80. Thorsen S, Halldorsson T, Bjerregaard A, Olsen S, Svensson J. Maternal and Early Life Iron Intake and Risk of Childhood Type 1 Diabetes: A Danish Case-Cohort Study. *Nutrients*. 2019 Mar 29;**11**(4):734.

81. Uusitalo U, Lee H-S, Andrén Aronsson C, Vehik K, Yang J, Hummel S, et al. Early Infant Diet and Islet Autoimmunity in the TEDDY Study. *Diabetes Care*. 2018 Mar;**41**(3):522–30.

82. Wadsworth EJ, Shield JP, Hunt LP, Baum JD. A case-control study of environmental factors associated with diabetes in the under 5s. *Diabet Med*. 1997;**14**(5):390–6.

83. Verge CF, Howard NJ, Irwig L, Simpson JM, Mackerras D, Silink M. Environmental factors in childhood IDDM. A population-based, case-control study. *Diabetes Care*. 1994;**17**(12):1381–9.

84. Viner RM, Hindmarsh PC, Taylor B, Cole TJ. Childhood body mass index (BMI), breastfeeding and risk of Type 1 diabetes: findings from a longitudinal national birth cohort. *Diabet Med*. 2008 Aug 29;**25**(9):1056–61.

85. Virtanen SM, Rasanen L, Aro A, Lindstrom J, Sippola H, Lounamaa R, et al. Infant feeding in Finnish children less than 7 yr of age with newly diagnosed IDDM. Childhood Diabetes in Finland Study Group. *Diabetes Care*. 1991;**14**(5):415–7.

86. Virtanen SM, Rasanen L, Aro A, Ylonen K, Lounamaa R, Tuomilehto J, et al. Feeding in infancy and the risk of type 1 diabetes mellitus in Finnish children. The “Childhood Diabetes in Finland” Study Group. *Diabet Med*. 1992;**9**(9):815–9.

87. Virtanen SM, Rasanen L, Aro A, Ylonen K, Lounamaa R, Akerblom HK, et al. Is children’s or parents’ coffee or tea consumption associated with the risk for type 1 diabetes mellitus in children? Childhood Diabetes in Finland Study Group. *Eur J Clin Nutr*. 1994;**48**(4):279–85.

88. Virtanen SM, Jaakkola L, Rasanen L, Ylonen K, Aro A, Lounamaa R, et al. Nitrate and nitrite intake and the risk for type 1 diabetes in Finnish children. Childhood Diabetes in Finland Study Group. *Diabet Med*. 1994;**11**(7):656–62.

89. Virtanen SM, Hypponen E, Laara E, Vahasalo P, Kulmala P, Savola K, et al. Cow’s milk consumption, disease-associated autoantibodies and type 1 diabetes mellitus: a follow-up study in siblings of diabetic children. Childhood Diabetes in Finland Study Group. *Diabet Med*. 1998;**15**(9):730–8.

90. Virtanen SM, Laara E, Hypponen E, Reijonen H, Rasanen L, Aro A, et al. Cow’s milk consumption, HLA-DQB1 genotype, and type 1 diabetes: a nested case-control study of siblings of children with diabetes. Childhood diabetes in Finland study group. *Diabetes*. 2000;**49**(6):912–7.

91. Virtanen SM, Kenward MG, Erkkola M, Kautiainen S, Kronberg-Kippila C, Hakulinen T, et al. Age at introduction of new foods and advanced beta cell autoimmunity in young children with HLA-conferred susceptibility to type 1 diabetes. *Diabetologia*. 2006;**49**(7):1512–21.

92. Virtanen SM, Takkinen H-M, Nevalainen J, Kronberg-Kippilä C, Salmenhaara M, Uusitalo L, et al. Early introduction of root vegetables in infancy associated with advanced ß-cell autoimmunity in young children with human leukocyte antigen-conferred susceptibility to Type 1 diabetes. *Diabet Med*. 2011 Aug;**28**(8):965–71.

93. Virtanen S, Uusitalo L, Kenward M, Nevalainen J, Uusitalo U, Kronberg-Kippilä C, et al. Maternal food consumption during pregnancy and risk of advanced β-cell autoimmunity in the offspring. *Pediatr Diabetes*. 2011 Mar;**12**(2):95–9.

94. Visalli N, Sebastiani L, Adorisio E, Conte A, De Cicco AL, D’Elia R, et al. Environmental risk factors for type 1 diabetes in Rome and province. *Arch Dis Child*. 2003;**88**(8):695–8.

95. Ziegler A-G, Schmid S, Huber D, Hummel M, Bonifacio E. Early infant feeding and risk of developing type 1 diabetes-associated autoantibodies. *JAMA*. 2003;**290**(13):1721–8.

96. Akerblom HK, Virtanen SM, Ilonen J, Savilahti E, Vaarala O, Reunanen A, et al. Dietary manipulation of beta cell autoimmunity in infants at increased risk of type 1 diabetes: a pilot study. *Diabetologia*. 2005;**48**(5):829–37.

97. Knip M, Virtanen SM, Seppä K, Ilonen J, Savilahti E, Vaarala O, et al. Dietary Intervention in Infancy and Later Signs of Beta-Cell Autoimmunity. *N Engl J Med*. 2010 Nov 11;**363**(20):1900–8.

98. Knip M, Åkerblom HK, Becker D, Dosch HM, Dupre J, Fraser W, et al. Hydrolyzed infant formula and early β-cell autoimmunity: A randomized clinical trial. *JAMA - J Am Med Assoc*. 2014;**311**(22):2279–87.

99. Knip M, Åkerblom HK, Al Taji E, Becker D, Bruining J, Castano L, et al. Effect of Hydrolyzed Infant Formula vs Conventional Formula on Risk of Type 1 Diabetes. *JAMA*. 2018 Jan 2;**319**(1):38.

100. Lampeter EF, Klinghammer A, Scherbaum WA, Heinze E, Haastert B, Giani G, et al. The Deutsche Nicotinamide Intervention Study: an attempt to prevent type 1 diabetes. DENIS Group. *Diabetes*. 1998;**47**(6):980–4.

101. Hummel S, Pfluger M, Hummel M, Bonifacio E, Ziegler A-G. Primary Dietary Intervention Study to Reduce the Risk of Islet Autoimmunity in Children at Increased Risk for Type 1 Diabetes: The BABYDIET study. *Diabetes Care*. 2011 Jun 1;**34**(6):1301–5.

102. Blom L, Dahlquist G, Nystrom L, Sandstrom A, Wall S. The Swedish childhood diabetes study--social and perinatal determinants for diabetes in childhood. *Diabetologia*. 1989;**32**(1):7–13.

103. Kimpimaki T, Erkkola M, Korhonen S, Kupila A, Virtanen SM, Ilonen J, et al. Short-term exclusive breastfeeding predisposes young children with increased genetic risk of Type I diabetes to progressive beta-cell autoimmunity. *Diabetologia*. 2001;**44**(1):63–9.

104. Miller MR, Yin X, Seifert J, Clare-Salzler M, Eisenbarth GS, Rewers M, et al. Erythrocyte membrane omega-3 fatty acid levels and omega-3 fatty acid intake are not associated with conversion to type 1 diabetes in children with islet autoimmunity: The Diabetes Autoimmunity Study in the Young (DAISY). *Pediatr Diabetes*. 2011 Dec;**12**(8):669–75.

105. Sørensen IM, Joner G, Jenum PA, Eskild A, Torjesen PA, Stene LC. Maternal serum levels of 25-hydroxy-vitamin D during pregnancy and risk of type 1 diabetes in the offspring. *Diabetes*. 2012;**61**(1):175–8.

106. Stene LC, Ulriksen J, Magnus P, Joner G. Use of cod liver oil during pregnancy associated with lower risk of Type I diabetes in the offspring. *Diabetologia*. 2000;**43**(9):1093–8.

107. Tapia G, Mårild K, Dahl SR, Lund-Blix NA, Viken MK, Lie BA, et al. Maternal and Newborn Vitamin D–Binding Protein, Vitamin D Levels, Vitamin D Receptor Genotype, and Childhood Type 1 Diabetes. *Diabetes Care*. 2019 Apr;**42**(4):553–9.

108. Virtanen SM, Niinistö S, Nevalainen J, Salminen I, Takkinen H-M, Kääriä S, et al. Serum fatty acids and risk of advanced β-cell autoimmunity: a nested case–control study among children with HLA-conferred susceptibility to type I diabetes. *Eur J Clin Nutr*. 2010 Aug 26;**64**(8):792–9.

109. Virtanen SM, Rasanen L, Ylonen K, Aro A, Clayton D, Langholz B, et al. Early introduction of dairy products associated with increased risk of IDDM in Finnish children. The Childhood in Diabetes in Finland Study Group. *Diabetes*. 1993;**42**(12):1786–90.

110. Virtanen SM, Saukkonen T, Savilahti E, Ylonen K, Rasanen L, Aro A, et al. Diet, cow’s milk protein antibodies and the risk of IDDM in Finnish children. Childhood Diabetes in Finland Study Group. *Diabetologia*. 1994;**37**(4):381–7.

111. Uusitalo U, Liu X, Yang J, Aronsson CA, Hummel S, Butterworth M, et al. Association of Early Exposure of Probiotics and Islet Autoimmunity in the TEDDY Study. *JAMA Pediatr*. 2016 Jan 1;**170**(1):20.

112. Krischer JP, Lynch KF, Lernmark A, Hagopian WA, Rewers MJ, She J-X, et al. Genetic and Environmental Interactions Modify the Risk of Diabetes-Related Autoimmunity by 6 Years of Age: The TEDDY Study. *Diabetes Care*. **40**(9):1194–202.

113. Uusitalo L, Kenward MG, Virtanen SM, Uusitalo U, Nevalainen J, Niinisto S, et al. Intake of antioxidant vitamins and trace elements during pregnancy and risk of advanced beta cell autoimmunity in the child. *Am J Clin Nutr*. 2008;**88**(2):458–64.

114. Fronczak CM, Baron AE, Chase HP, Ross C, Brady HL, Hoffman M, et al. In utero dietary exposures and risk of islet autoimmunity in children. *Diabetes Care*. 2003;**26**(12):3237–42.

115. Marjamäki L, Niinistö S, Kenward MG, Uusitalo L, Uusitalo U, Ovaskainen ML, et al. Maternal intake of vitamin D during pregnancy and risk of advanced beta cell autoimmunity and type 1 diabetes in offspring. *Diabetologia*. 2010;**53**(8):1599–607.

116. Knekt P, Reunanen A, Marniemi J, Leino A, Aromaa A. Low vitamin E status is a potential risk factor for insulin-dependent diabetes mellitus. *J Intern Med*. 1999;**245**(1):99–102.

117. Uusitalo L, Knip M, Kenward MG, Alfthan G, Sundvall J, Aro A, et al. Serum alpha-tocopherol concentrations and risk of type 1 diabetes mellitus: a cohort study in siblings of affected children. *J Pediatr Endocrinol Metab*. 2005;**18**(12):1409–16.

118. Uusitalo L, Nevalainen J, Niinistö S, Alfthan G, Sundvall J, Korhonen T, et al. Serum α- and γ-tocopherol concentrations and risk of advanced beta cell autoimmunity in children with HLA-conferred susceptibility to type 1 diabetes mellitus. *Diabetologia*. 2008 May 4;**51**(5):773–80.

119. Prasad M, Takkinen H-M, Nevalainen J, Ovaskainen M-L, Alfthan G, Uusitalo L, et al. Are serum α- and β-carotene concentrations associated with the development of advanced beta-cell autoimmunity in children with increased genetic susceptibility to type 1 diabetes? *Diabetes Metab*. 2011 Apr;**37**(2):162–7.

120. Mattila M, Erlund I, Lee H-S, Niinistö S, Uusitalo U, Andrén Aronsson C, et al. Plasma ascorbic acid and the risk of islet autoimmunity and type 1 diabetes: the TEDDY study. *Diabetologia*. 2020 Feb 14;**63**(2):278–86.

121. Kyvsgaard JN, Overgaard AJ, Jacobsen LD, Thorsen SU, Pipper CB, Hansen TH, et al. Low perinatal zinc status is not associated with the risk of type 1 diabetes in children. *Pediatr Diabetes*. 2017 Nov;**18**(7):637–42.

122. Kyvsgaard J, Overgaard A, Thorsen S, Hansen T, Pipper C, Mortensen H, et al. High Neonatal Blood Iron Content Is Associated with the Risk of Childhood Type 1 Diabetes Mellitus. *Nutrients*. 2017 Nov 6;**9**(11):1221.
